# Hierarchical micro/nanostructured silver hollow fiber boosts electroreduction of carbon dioxide

**DOI:** 10.1038/s41467-022-30733-6

**Published:** 2022-06-02

**Authors:** Shoujie Li, Wei Chen, Xiao Dong, Chang Zhu, Aohui Chen, Yanfang Song, Guihua Li, Wei Wei, Yuhan Sun

**Affiliations:** 1 Low-Carbon Conversion Science and Engineering Center, Shanghai Advanced Research Institute, Chinese Academy of Sciences, Shanghai, 201210 PR China; 2 University of Chinese Academy of Sciences, Beijing, 100049 PR China; 3 School of Physical Science and Technology, ShanghaiTech University, Shanghai, 201203 PR China

**Keywords:** Electrocatalysis, Electrochemistry, Electrocatalysis, Sustainability

## Abstract

Efficient conversion of CO_2_ to commodity chemicals by sustainable way is of great significance for achieving carbon neutrality. Although considerable progress has been made in CO_2_ utilization, highly efficient CO_2_ conversion with high space velocity under mild conditions remains a challenge. Here, we report a hierarchical micro/nanostructured silver hollow fiber electrode that reduces CO_2_ to CO with a faradaic efficiency of 93% and a current density of 1.26 A · cm^−2^ at a potential of −0.83 V *vs*. RHE. Exceeding 50% conversions of as high as 31,000 mL · g_cat_^−1^ · h^−1^ CO_2_ are achieved at ambient temperature and pressure. Electrochemical results and time-resolved operando Raman spectra demonstrate that enhanced three-phase interface reactions and oriented mass transfers synergistically boost CO production.

## Abstract (web summary)

An activated silver hollow fiber electrode enables enhanced triphasic interface reactions and oriented mass transfers synergistically, facilitating efficient CO_2_ conversion (> 50%) to boost CO production with a current density of 1.26 A · cm^−2^.

## Introduction

Large-scale CO_2_ utilization, abating carbon emissions while producing commodity chemicals, is a promising strategy for achieving carbon neutrality^1–3^. Thermocatalytic routes such as CO_2_ hydrogenation to methanol or other compounds exhibit industrial potential but suffering from the dilemmas of hydrogen sources and severe reaction conditions^4–6^. Recently, electrocatalytic CO_2_ conversion has emerged as a remarkable technology that benefits from the desirable coupling of renewable electricity transition and CO_2_ utilization^7–10^. However, the efficiency of CO_2_ electroreduction is much inferior to thermocatalytic CO_2_ conversion processes due to the limited CO_2_ solubility in electrolyte solutions and divergent kinetics^9,11^. One tactic for addressing these issues is adopting gas-diffusion electrodes that consist of highly active catalysts decorated with superhydrophobic polytetrafluoroethylene and conductive carbon layers^12–15^. Regarding such gas-diffusion electrodes, these multiple components are assembled via subtle procedures to build complicated configurations, which could hinder their practical scale-up. Although large current densities and high faradaic efficiencies of various products have been realized over these gas-diffusion electrodes^12,16^, their CO_2_ conversion rates especially at high flow rates are still lower than 20% (Supplementary Table 1). Furthermore, three-dimensional hollow fiber electrodes with a compact structure exhibit promising potentials in efficient and high-rate CO_2_ electroreduction by virtue of improved mass transport^17–21^. To date, the hollow fiber electrodes still deliver too limited current densities (≤200 mA ∙ cm^−2^) to afford an economically viable CO_2_ electrochemical conversion^19,20^.

**Fig. 1:**
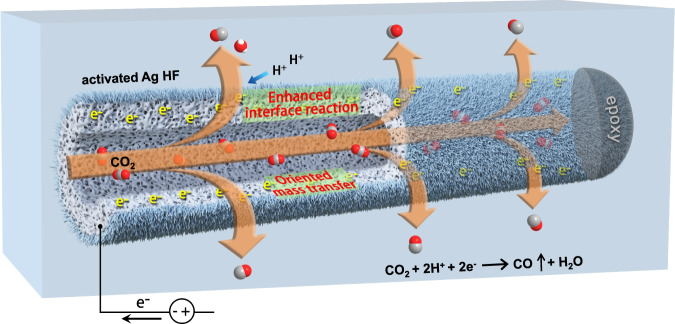
Function outline of hollow-fiber silver electrode. Schematic illustration of hierarchical micro/nanostructured silver hollow fiber for boosting CO_2_ electroreduction to CO.

Herein, we report a hollow fiber electrode with hierarchical micro/nanostructures composed of only metallic silver (Ag) for electroreducing CO_2_ to CO. Such a porous hollow-fiber Ag electrode acting as a CO_2_ disperser can not only enhance three-phase interface reactions but also guide mass transfers during electrolysis (Fig. 1). As a result, CO_2_ conversions exceed 50% at a high space velocity of 31,000 mL ∙ g_cat_^−1^ ∙ h^−1^ corresponding to a flow rate of 60 mL ∙ min^−1^ under ambient conditions, maintaining stable large current densities (~1.26 A ∙ cm^−2^) and high CO faradaic efficiencies (~93%) in a continuous test for a long lifespan, and this represents an encouraging headway in sustainable CO_2_ utilization.

## Results and discussion

### Structure and composition

The silver hollow fiber (Ag HF) was first fabricated by a combined phase-inversion/sintering process from commercial Ag powder (Supplementary Fig. 1), followed by electrochemical redox activation treatments to obtain an activated Ag HF (Supplementary Figs. 2–4). The fused silver particles from the outer surface of Ag HF rather than spherical ones in pristine Ag powder (Supplementary Figs. 5, 6) implied that a well-integrated substrate was formed by sintering during Ag HF fabrication.

**Fig. 2:**
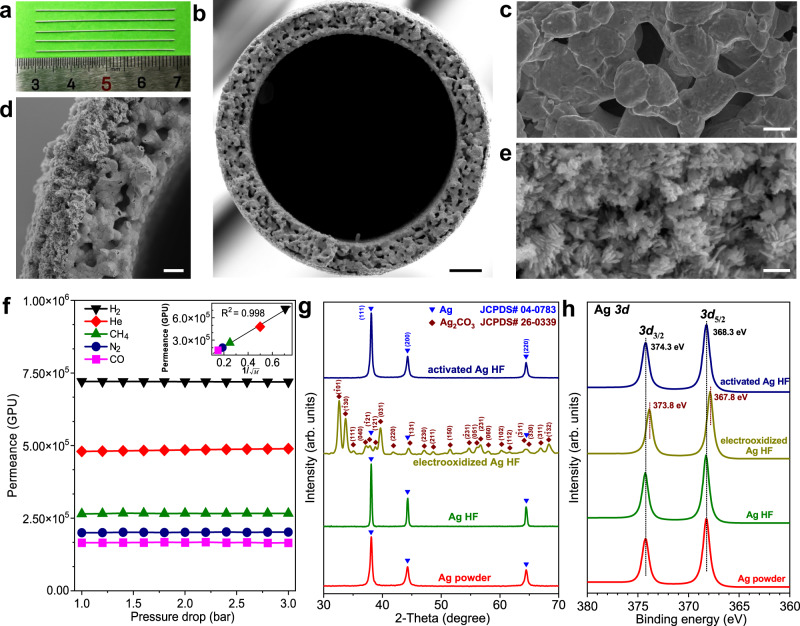
Structural and compositional characterization. **a** Optical image of the as-fabricated Ag HF tubes. SEM images of **b**, **d**, cross sections and **c**, **e**, outer surfaces of **b**, **c**, Ag HF and **d**, **e**, activated Ag HF. Scale bars in **b**, **c**, **d** and **e** are 50 µm, 5 µm, 10 µm and 500 nm, respectively. **f** Gas permeances of activated Ag HF. GPU denotes gas permeation unit. Inset: the permeances (at 1.0 bar) are proportional to the inverse square root of gas molecular weight. **g** XRD patterns, and **h**, XPS spectra of Ag HF and activated Ag HF.

The surfaces of the slender Ag HF tubes exhibited metallic luster (Fig. 2a), and their scanning electron microscopy (SEM) images showed abundant micrometer-sized pores on the outer/inner surfaces and interconnected pores inside the wall of Ag HF (Fig. 2b, c and Supplementary Figs. 5b, 7a). By the electrochemical activation treatments to reconstruct the outer surface of Ag HF, partly ordered nanorods gathered at the outer region, configuring hierarchical micro/nanostructures in activated Ag HF (Fig. 2d, e and Supplementary Fig. 7b). The effective porosities of Ag HF and activated Ag HF, as determined by gas permeation tests, were 38% and 32%, respectively (Supplementary Fig. 8 and Fig. 2f). Both X-ray diffraction (XRD) (Fig. 2g) and X-ray photoelectron spectroscopy (XPS) (Fig. 2h) verified that the bulk and surface compositions of Ag HF and activated Ag HF were identical with metallic silver (Supplementary Figs. 9, 10).

### Electrocatalytic performance

The Ag HF array comprising ten tubes was used as the working electrode (Supplementary Fig. 11) and subjected to the potentiostatic electrolysis of CO_2_ after the electrochemical activation treatments. Over a single tube of the activated Ag HF electrode, CO_2_ molecules were highly dispersed via penetrating through the porous wall of activated Ag HF, and CO was produced at the gas-liquid-solid three-phase interface sites (Fig. 1). The CO partial current density (*j*_*CO*_), i.e., the total current density × CO faradaic efficiency, showed superior in the relatively concentrated solutions with the best performance in 1.5 M KHCO_3_ (Supplementary Fig. 12). On basis of the intrinsic structure characteristics of activated Ag HF, the CO_2_ flow rate was fixed at 60 mL ∙ min^−1^ during CO_2_ electroreduction to obtain the optimal electrocatalytic performance (Supplementary Fig. 13).

**Fig. 3:**
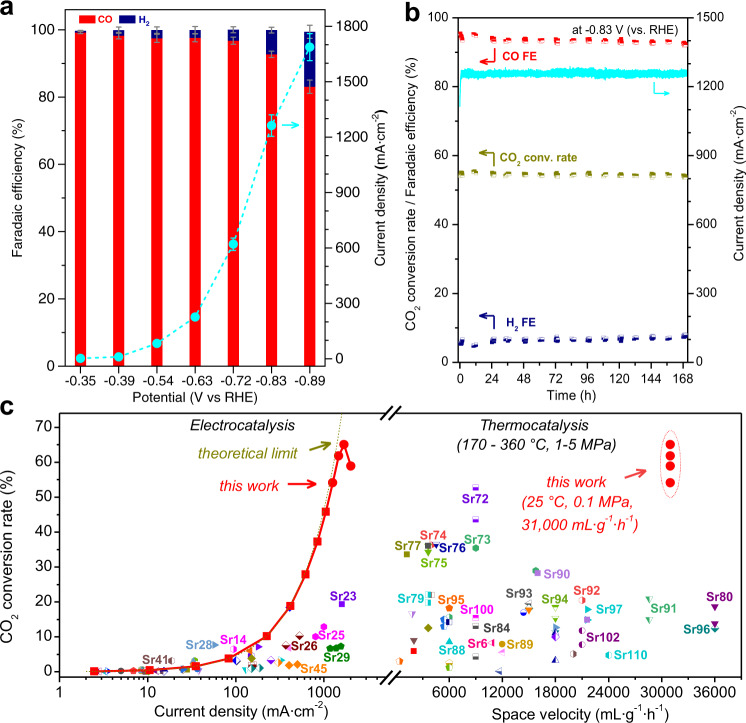
Performance of activated Ag HF. **a** CO and H_2_ faradaic efficiencies, and total current densities in the potential range of −0.35 to −0.89 V. Error bars in **a** were obtained from the average of six individual tests. **b** Long-term performance at −0.83 V. **c** CO_2_ conversion rates with a space velocity of 31,000 mL · g_cat_^−1^ · h^−1^ at different current densities, and their overall comparison with other electrocatalytic and thermocatalytic CO_2_ conversions. All comparison data points in **c** are from the references summarized in Supplementary Table S1, and the key data points are referred to the corresponding Supplementary references Srx, where x represents the reference number in Supplementary Information. CO_2_-saturated 1.5 M KHCO_3_ as the electrolyte solution, and the CO_2_ flow rate of 60 mL · min^−1^.

As shown in Fig. 3a, only CO and H_2_ were detected over the activated Ag HF electrode (Supplementary Fig. 14) with their faradaic efficiency sum almost equaling 100% in the potential range of −0.35 to −0.89 V. The H_2_ faradaic efficiency was always less than 3% up till −0.72 V while the total current density kept growing rapidly. At −0.83 V, the CO faradaic efficiency was 93% with the total current density of 1.26 A ∙ cm^−2^. Then, the CO faradaic efficiency dropped down to 83% at −0.89 V, although the total current density increased to 1.69 A ∙ cm^−2^, implying a rising hydrogen evolution reaction (HER) at more negative potentials. Further evaluation of the durability of the activated Ag HF electrode was performed in a continuous CO_2_ electrolysis test operated at −0.83 V. The CO faradaic efficiency remained between 93 and 92% and the total current density fluctuated between 1.26 and 1.24 A ∙ cm^−2^, manifesting no declining sign for 170 h (Fig. 3b). The postreaction XRD (Supplementary Fig. 9) and XPS (Supplementary Fig. 10) revealed the unchanged compositions of activated Ag HF after the electrolysis, and the corresponding structural features were also highly similar to those before the electrolysis (Supplementary Fig. 8), which were responsible for the stable CO_2_ electroreduction performance.

CO_2_ conversion rate is an important common criterion for both thermocatalytic and electrocatalytic processes, and their overall comparisons are displayed in Fig. 3c. In the current density range from 2 to 400 mA ∙ cm^−2^, the CO_2_ conversion rates of activated Ag HF were comparable to those over other prominent catalysts reported in electrocatalysis. Note that the CO_2_ conversion rates further increased rapidly with negative-shifting potentials (Supplementary Fig. 15) and increasing current densities, which approached the theoretical predictions, far outperforming the previously reported electrocatalysts (Fig. 3c and Supplementary Table 1). At −0.83 V, the CO_2_ conversion rate of activated Ag HF was 54% with sustained performance during the long-term test (Fig. 3b). Furthermore, the thermocatalytic catalysts run at high temperature (170–360 °C) and pressure (1–5 MPa) delivered relatively low CO_2_ conversion rates (less than 22%) at high space velocities (≥18,000 mL ∙ g_cat_^−1^ ∙ h^−1^) (Fig. 3c and Supplementary Table 1). In contrast, the CO_2_ conversion rates over the activated Ag HF electrode at CO_2_ space velocity of 31,000 mL ∙ g_cat_^−1^ ∙ h^−1^ exceeded 28% just from −0.72 V and 54% from −0.83 V under ambient conditions, even higher than those over low-space-velocity-run catalysts of CO_2_ hydrogenation (Supplementary Fig. 15 and Supplementary Table 1).

Previous studies^22–25^ reported Ag electrocatalysts possessing the capability to selectively reduce CO_2_ to CO, but their current densities of long-term tests remained below 200 mA∙cm^−2^ (Supplementary Table 1), far from the ≥400 mA ∙ cm^−2^ regime for industrial applications^26,27^. In sharp contrast, our activated Ag HF electrode manifested the sustained large current density (~1.26 A ∙ cm^−2^) with high CO faradaic efficiency (~93%), implying a striking promotion in the intrinsic activity of Ag.

### Electrochemical characterization

**Fig. 4:**
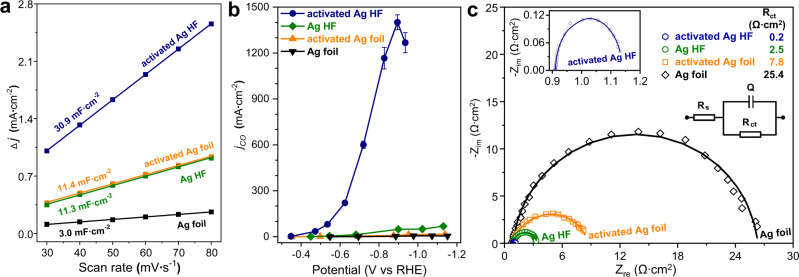
Electrochemical characterization. **a** Plot of *Δj* (the difference of cathodic and anodic current densities, *j*_*c*_–*j*_*a*_) against the scan rates from cyclic voltammetry curves (Supplementary Fig. 16), **b** CO partial current density comparison, and error bars were obtained from the average of six individual tests. **c** EIS Nyquist plots of activated Ag HF, Ag HF, activated Ag foil and Ag foil. The inset in **c** shows the equivalent circuit.

The electrochemically active surface areas (ECSAs) of activated Ag HF, Ag HF, activated Ag foil and Ag foil were determined by measuring their double-layer capacitance (*C*_*dl*_) values via their cyclic voltammetry curves (Supplementary Fig. 16). Although the ECSA of activated Ag HF was only nearly 3 times that of activated Ag foil (Fig. 4a), the *j*_*CO*_ of activated Ag HF with the lower overpotential was almost two orders of magnitude larger than that of activated Ag foil (Fig. 4b and Supplementary Fig. 17). This implied that the high ECSA only played a secondary role in efficiently producing CO over activated Ag HF.

Furthermore, the electrochemical impedance spectroscopy (EIS) measurements were performed to study the electron transfer behaviors of the activated Ag HF, Ag HF, activated Ag foil and Ag foil electrodes, and their impedance spectra in the complex plane (Nyquist plot) are presented in Fig. 4c. The activated Ag HF electrode exhibited the smallest *R*_*ct*_ (0.2 Ω ∙ cm^2^) compared with those of Ag HF (2.5 Ω ∙ cm^2^), activated Ag foil (7.8 Ω ∙ cm^2^) and Ag foil (25.4 Ω ∙ cm^2^), indicating the most favorable CO_2_ reduction kinetics over activated Ag HF.

### CO_2_ dispersion effects

**Fig. 5:**
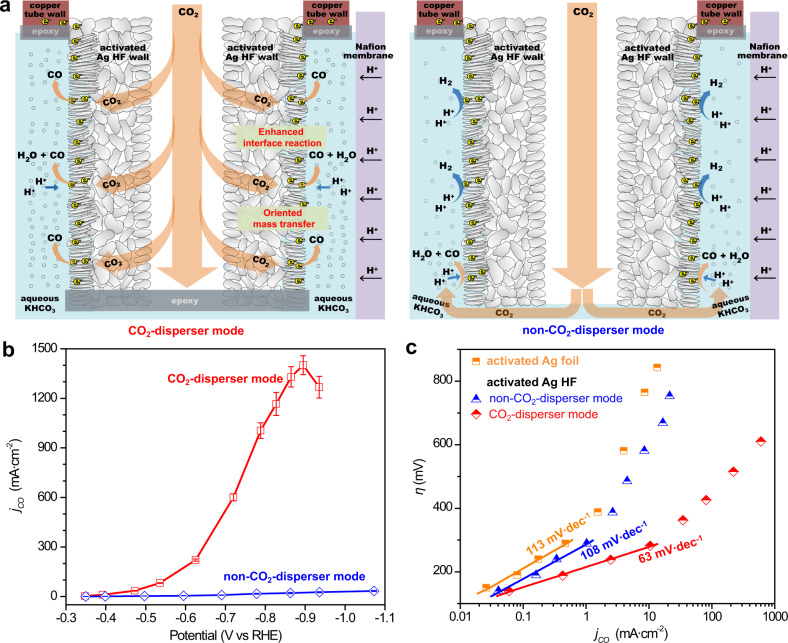
CO_2_ dispersion effect studies. **a** Schematic illustrations showing the processes of CO_2_ electroreduction over activated Ag HF with the CO_2_-disperser mode and the non-CO_2_-disperser mode, respectively, and **b**, their CO partial current densities. Error bars in **b** were obtained from the average of six individual tests. **c** Tafel slopes of activated Ag foil and activated Ag HF with different dispersion modes.

In comparison with other counterparts, activated Ag HF was distinguished in that it possessed the unique CO_2_ diffusion manner acting as a CO_2_ disperser. That is the restrained environment of activated Ag HF offered a scenario that CO_2_ were compulsively interacted with active sites when penetrating through the porous wall (Fig. 5a), resulting in enhanced three-phase interface reactions and optimized kinetics to produce CO efficiently. Obviously, such CO_2_ dispersion effects of activated Ag HF vanished just switching to non-CO_2_-disperser mode (Fig. 5a, b). The comparison of CO partial current densities over the activated Ag HF electrode with the CO_2_-disperser and non-CO_2_-disperser modes is shown in Fig. 5b. As the potential negatively shifted from −0.35 to −0.94 V, the *j*_*CO*_ of activated Ag HF using the CO_2_-disperser mode increased quickly and reached the maximum value of 1402 mA ∙ cm^−2^ at −0.89 V, then slightly decreased to 1268 mA ∙ cm^−2^ at −0.94 V. In sharp contrast, activated Ag HF using the non-CO_2_-disperser mode always delivered rather low *j*_*CO*_ values and showed a maximum value of only 33 mA ∙ cm^−2^ at −1.07 V. These results indicated that the CO_2_-disperser mode was uniquely superior to the non-CO_2_-disperser mode.

It is generally known that large current densities in water-based electrolytes will lead to the high local pH near the electrode surface, which inhibits the HER and increases CO_2_ reduction due to the proton depletion^28–30^. Besides such CO_2_ reduction promotion from local pH variation, our activated Ag HF electrode could deliver large and stable current density lying in the enhanced three-phase interface reactions and mass transfers. That is the high-flow-rate CO_2_ rushing out from the porous hollow fiber wall to react on the abundant active Ag nanorods at the outer region, thereby maintaining extremely high local CO_2_ concentration at three-phase interface sites for significant CO_2_ electroreduction rather than HER^31,32^. Consequently, such a hollow fiber dispersion design maximizes the efficiency of the three-phase reaction interfaces, showing a promising potential compared with the conventional membrane electrode assembly.

Furthermore, a comparison of the kinetic data extracted from the Tafel plots, namely, the overpotential (*η*) versus log(*j*_*CO*_), was made for activated Ag foil and activated Ag HF with the non-CO_2_-disperser and CO_2_-disperser modes, as shown in Fig. 5c. In the low overpotential regime (i.e., Tafel linearity) of −140 to −290 mV, a Tafel slope of 113 mV ∙ dec^−1^ was obtained for activated Ag foil, close to the value of 118 mV ∙ dec^−1^ expected for a rate-determining single-electron transfer at the electrode surface^33^. This result indicated that the initial one-electron transfer for CO_2_ activation over activated Ag foil to form an adsorbed *COO^−^ intermediate (Supplementary Fig. 18) was the rate-determining step, in consistence with previous reports^33,34^. Furthermore, a dramatically increased Tafel slope for activated Ag foil was observed at relatively high overpotentials, implying that CO_2_ electroreduction likely reached a mass transfer limitation^34,35^. In contrast, activated Ag HF with the CO_2_-disperser mode showed the smallest Tafel slope (63 mV ∙ dec^−1^), close to the theoretical value of 59 mV ∙ dec^−1^ ^35,36^, suggesting a fast initial one-electron transfer step to form *COO^−^ and a subsequent slower chemical reaction as the rate-determining step (Supplementary Fig. 18). The Tafel slope of activated Ag HF with the CO_2_-disperser mode at high overpotential regime was also much smaller than that of activated Ag foil, implying the favorable mass transfer. Interestingly, the Tafel slope for activated Ag HF with the non-CO_2_-disperser was 108 mV ∙ dec^−1^, which was close to the value of 113 mV ∙ dec^−1^ for activated Ag foil. This result indicated that both had the same rate-determining step, namely, the initial one-electron transfer, which also confirmed the superiority of the CO_2_-disperser mode for CO_2_ reduction. In addition, the high-overpotential Tafel slope for activated Ag HF with the non-CO_2_-disperser mode was closer to that for activated Ag foil. These results indicated that the improved initial one-electron transfer and mass transfer jointly enhanced the intrinsic CO_2_ reduction activity of activated Ag HF with the CO_2_-disperser mode, resulting in high selectivity and activity for the electrocatalytic reduction of CO_2_ to CO.

### Mechanistic studies

**Fig. 6:**
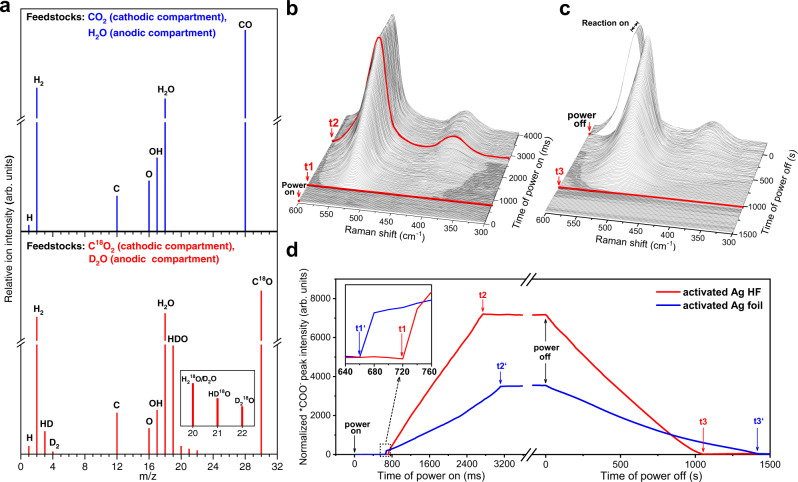
Isotopic trace and time-resolved operando Raman results. **a** Mass spectrometric detections of the cathodic products over activated Ag HF using unlabeled feedstocks (upper) and isotopically labeled C^18^O_2_ and D_2_O feedstocks (lower), respectively. **b**, **c** Time-resolved operando Raman spectra showing the formation, evolution and dissipation of intermediates over activated Ag HF. **d** Comparison of the normalized *COO^−^ peak intensities of activated Ag HF and activated Ag foil during the power-on and power-off stages.

Isotope-labelling experiments were conducted using C^18^O_2_ and D_2_O as feedstocks to reveal the mass migrations involved in CO_2_ electroreduction over activated Ag HF. The results in Fig. 6a and Supplementary Fig. 19 suggested that CO production was derived from the CO_2_ dissociation to form one O atom, which coupled with two protons (H^+^) to generate H_2_O (Fig. 5a), while anodic water oxidation released and supplied protons to participate in cathodic CO_2_ reduction. This indicated that the product CO originated from the reduction of CO_2_ and the anodic reaction maintained the proton and charge balances of the overall CO_2_ electroreduction reaction.

Time-resolved operando Raman spectroscopy was resorted to further reveal the formation and evolution of key intermediates during CO_2_ electroreduction. The typical operando Raman spectra of both activated Ag HF and activated Ag foil (Supplementary Fig. 20) showed two Raman bands at 532 and 390–410 cm^−1^, corresponding to adsorbed *COO^−^ and *COOH intermediates, respectively^37,38^, besides bicarbonate ion related peaks above 1000 cm^−1^ ^39–41^. The chronological appearances of *COO^−^ and *COOH (Fig. 6b and Supplementary Fig. 21c) implied the step-by-step reduction of CO_2_, i.e., the initial step to form *COO^−^ and the second step to form *COOH, in agreement with the proposed mechanism (Supplementary Fig. 18).

After power-on for 720 ms (**t1**), *COO^−^ Raman peak over activated Ag HF appeared, and then its intensity increased quickly and reached the maximum at 2720 ms (**t2**) (Fig. 6b and d). Regarding activated Ag foil, the *COO^−^ peak appeared at 660 ms (**t1’**), and the peak intensity reached a maximum at 3080 ms (**t2’**) (Supplementary Fig. 21c and Fig. 6d). Compared to activated Ag foil, more *COO^−^ intermediates were formed and adsorbed over activated Ag HF in a shorter time (Fig. 6d and Supplementary Figs. 21a, c), implying the superior capability of CO_2_ activation over activated Ag HF, which probably profited from the reduced CO_2_ diffusion distance in the CO_2_-disperser mode.

Subsequently, we investigated the variation of adsorbed *COO^−^ over activated Ag HF and activated Ag foil during the power-off stages (Fig. 6c and Supplementary Fig. 21d). As soon as the power was off, the 532 cm^−1^ ν_*COO_- redshifted abruptly for both electrodes due to the Stark effect^41–44^, indicating the distinct impact of electric field on the adsorption of intermediates (Supplementary Fig. 22). The *COO^−^ peak vanished over activated Ag HF after power-off for 1050 s (**t3**) (Fig. 6c, d), whereas over activated Ag foil after power-off for 1400 s (**t3’**) (Supplementary Fig. 21d and Fig. 6d). Moreover, the dissipation time of *COO^−^ over activated Ag HF with non-CO_2_-disperser mode was 1380 s (**t3”**) (Supplementary Fig. 23), which was close to that (1400 s, **t3’**) over activated Ag foil. The faster dissipation of adsorbed *COO^−^ benefited from the one-way CO_2_ flow manner of activated Ag HF with the CO_2_-disperser mode (Fig. 6d). While direct Raman observations on the formation and desorption of *CO species were not available at present, to be explored in further study.

Consequently, these Raman results suggested that the oriented mass transfers induced by the CO_2_-disperser mode of activated Ag HF could not only favor the diffusion of CO_2_ to active sites but also facilitate the desorption of adsorbed species from the electrode surface, resulting in the improved overall kinetics of CO_2_ reduction.

Our results demonstrate that the electrocatalytic performance of CO_2_ reduction could be greatly improved by adopting the micro/nanostructured hollow fiber configuration of silver, which provides new opportunities for heightening three-phase interface reactions and mass transfer kinetics simultaneously. In addition, the single composition and tough framework with simple fabrication procedures enable activated Ag HF to become an ideal industrial electrode with excellent durability. This work represents an encouraging headway in CO_2_ electroreduction that may lead to scalable applications.

## Methods

See the Supplementary Information for detailed description of the methods employed in this study.

### Chemicals and materials

Ag powder (99.9%, 50 nm) was purchased from Ningbo Jinlei Nano Materials Co., Ltd. Ag foil (99.9%, 1 mm thick) was purchased from Shanghai Macklin Biochemical Technology Co., Ltd. Polyetherimide (PEI) was purchased from Saudi Basic Industries Corporation (SABIC). N-Methyl-2-pyrrolidone (NMP) and potassium bicarbonate (KHCO_3_) were purchased from Sinopharm Chemical Reagent Co., Ltd. Nafion 117 proton exchange membranes (PEM) with an average thickness of 183 µm were purchased from DuPont. 3-Trimethylsilyl-1-propane sulfonic acid sodium salt (DSS) was purchased from Sigma-Aldrich. Isotope-labeled C^18^O_2_ (purity: 97 at.%) was purchased from Sigma-Aldrich. Deuterium oxide (D_2_O) was purchased from Sigma-Aldrich. All chemicals were used as received without further purification. Electrolyte solutions were prepared using 18.2 MΩ H_2_O (ultrapure water, from Master-S30UVF water purification system).

### Catalyst preparation

Ag HF was first fabricated by a combined phase-inversion/sintering process, and then was treated by electrochemical redox activation to obtain activated Ag HF. More details can be found in Supplementary Preparations section as well as Supplementary Figs. 1 and 2.

### Physical characterization

The cross-section and surface morphologies were observed by scanning electron microscopy (SEM) with a SUPRRATM 55 microscope using an accelerating voltage of 5.0 kV. Transmission electron microscopy (TEM) investigations were conducted with a JEM-ARM300F microscope operated at 300 kV. X-ray diffraction (XRD) measurements were performed on a Rigaku Ultima 4 X-ray diffractometer using a Cu *Kα* radiation source (λ = 1.54056 Ǻ) at 40 kV and 40 mA. X-ray photoelectron spectroscopy (XPS) was conducted using a Quantum 2000 Scanning ESCA Microprobe instrument with a monochromatic Al *Kα* source (1486.6 eV). The binding energies in all XPS spectra were calibrated according to the C 1 s peak (284.8 eV).

### Electrochemical characterization

Electrochemical characterization was performed on a Biologic VMP3 potentiostat in a gas-tight two-compartment electrolysis cell equipped with a KCl-saturated Ag/AgCl reference electrode and a platinum mesh (3 cm × 3 cm) counter electrode. The electrochemically active surface area (ECSA) of the electrode was evaluated by the double-layer capacitance (*C*_*dl*_). The *C*_*dl*_ was determined by performing cyclic voltammetry (CV) in the potential range of 0.4 to 0.5 V (*vs*. RHE) at different scan rates in CO_2_-saturated 1.5 M KHCO_3_. The electrochemical impedance spectroscopy (EIS) measurements were performed in CO_2_-saturated 1.5 M KHCO_3_ at −0.83 V (*vs*. RHE), and the frequency limits were typically set in the range of 0.1 Hz to 100 kHz with a voltage amplitude of 10 mV. Prior to the experiments, the electrolysis cell was vacuumized and then purged with CO_2_ for 30 min, after which CO_2_ was continuously delivered into the cathodic compartment at a constant rate of 10 mL · min^−1^. All the applied potentials were recorded against the KCl-saturated Ag/AgCl reference electrode and then converted to those versus the reversible hydrogen electrode (RHE) with *iR* compensation by the following equation: $E\left({vs}.{RHE}\right)=E\left({vs}.{Ag}/{AgCl}\right)+0.197V+0.0591V\times {pH}+0.85i{R}_{s}$, where *E (vs. Ag/AgCl)* is the applied potential, *pH* is the *pondus hydrogenii* value of the electrolyte solutions with different concentrations (Supplementary Table 2), *i* is the current density at each applied potential, and *R*_*s*_ is the solution resistance obtained by EIS measurements (Supplementary Table 2), and 85% *iR* compensation was applied to correct the potential manually. All applied potentials in the main text and Supplementary Information referred to the RHE unless otherwise stated.

### CO_2_ electroreduction tests

The potentiostatic electroreductions of CO_2_ over all electrodes were performed at ambient temperature and pressure on the Biologic VMP3 potentiostat using the gas-tight electrolysis cell. The cathodic and anodic compartments were separated by a Nafion 117 membrane, and the electrolysis cell was equipped with a KCl-saturated Ag/AgCl reference electrode in the cathodic compartment and a platinum mesh counter electrode in the anodic compartment. More details can be found in Supplementary CO_2_ Electroreduction and Product Quantifications section as well as Supplementary Fig. 14.

### Isotopic trace

The isotope-labelling experiments were conducted under the same electrolysis reaction conditions that used C^18^O_2_ and D_2_O as feedstocks. The feedstocks were supplied into the cathodic and anodic compartments of the electrolysis cell according to the following four situations: (**I**) nonisotope-labeled CO_2_ was fed to the cathodic compartment, and nonisotope-labeled H_2_O was used as the solvent in the anodic compartment; (**II**) nonisotope-labeled CO_2_ was fed to the cathodic compartment, and D-labeled D_2_O was used as the solvent in the anodic compartment; (**III**) ^18^O-labeled C^18^O_2_ was fed to the cathodic compartment, and nonisotope-labeled H_2_O was used as the solvent in the anodic compartment; (**IV**) ^18^O-labeled C^18^O_2_ was fed to the cathodic compartment, and D-labeled D_2_O was used as the solvent in the anodic compartment. The gas-phase exhausts from the cathodic compartment were first vented into 10 M NaOH solution to absorb unreacted CO_2_, and then the residual gases were detected by mass spectrometry (MS, GSD320-OmniStar, Pfeiffer Vacuum Corp., Germany). The offline catholyte sampled after 30-min electrolysis was placed into a water bath of 80 °C and then introduced into the mass spectrometer by a CO_2_ flow of 10 mL ∙ min^−1^. The MS data were analyzed and identified using QUADERA Version 4.60 software.

### Time-resolved operando Raman spectroscopy

Time-resolved operando Raman measurements were carried out on a Raman spectrometer (i-Raman Pro BWS475-532H, B&W Tek Corp.) using a 532 nm excitation laser with a laser power of 25 mW. The Raman shift was calibrated to 520 cm^−1^ using a Si wafer. Activated Ag HF and activated Ag foil were used as the working electrodes in the electrolysis cell for the operando Raman measurements, respectively. The electrolyte solution was CO_2_-saturated 1.5 M KHCO_3_ and the CO_2_ flow rate was kept at 60 mL∙min^−1^ for all Raman measurements. In order to decrease the disturbance of bubbles, the focus points of all Raman tests were generally close to the bottom region of the hollow fiber electrodes, where the bubbles were relatively sparse (Supplementary Fig. 11d). Raman spectra at the stable states of CO_2_ electroreduction over a wider range of 300 to 2000 cm^−1^ were also obtained for the purpose of identifying the peaks from adsorbed bicarbonate ions or other possible species (Supplementary Fig. 20). Operando Raman spectra were recorded continuously within a range of 300 to 600 cm^−1^ without any time intervals (Supplementary Fig. 21), and in the meantime the potentials of CO_2_ electroreduction were fixed at −0.83 and −0.69 V for activated Ag HF and activated Ag foil, respectively, which corresponded to their favorable electrocatalytic performance, i.e., ~1.26 A · cm^−2^ for activated Ag HF and 12 mA · cm^−2^ for activated Ag foil. Each Raman curve was obtained using a commercially available data-processing program (BWSpec 4.11 software), which was further subjected to smoothing treatment using commercial Origin 9.2 software before assembly of the operando Raman spectra. In addition, frequent power-on and power-off switching was applied during CO_2_ electroreduction, which was monitored by Raman spectra in a range of 300 to 600 cm^−1^ to confirm the reproducibility of the Stark effect in our tests (Supplementary Fig. 22).

## Supplementary information

Supplementary information

## Data Availability

All data supporting the findings of the study within this paper and Supplementary Information are available in the Source data file. Source data are provided with this paper.

## Source data

Source Data

## References

[1] Chu S, Majumdar A (2012). Opportunities and challenges for a sustainable energy future. Nature.

[2] Mallapaty S (2020). How China could be carbon neutral by mid-century. Nature.

[3] Jordaan SM, Wang C (2021). Electrocatalytic conversion of carbon dioxide for the Paris goals. Nat. Catal..

[4] Graciani J (2014). Highly active copper-ceria and copper-ceria-titania catalysts for methanol synthesis from CO_2_. Science.

[5] Gao P (2017). Direct conversion of CO_2_ into liquid fuels with high selectivity over a bifunctional catalyst. Nat. Chem..

[6] Hu J (2021). Sulfur vacancy-rich MoS_2_ as a catalyst for the hydrogenation of CO_2_ to methanol. Nat. Catal..

[7] Rabinowitz JA, Kanan MW (2020). The future of low-temperature carbon dioxide electrolysis depends on solving one basic problem. Nat. Commun..

[8] Lin S (2015). Covalent organic frameworks comprising cobalt porphyrins for catalytic CO_2_ reduction in water. Science.

[9] Wu Y, Jiang Z, Lu X, Liang Y, Wang H (2019). Domino electroreduction of CO_2_ to methanol on a molecular catalyst. Nature.

[10] Li F (2020). Molecular tuning of CO_2_-to-ethylene conversion. Nature.

[11] Liu M (2016). Enhanced electrocatalytic CO_2_ reduction via field-induced reagent concentration. Nature.

[12] Lv JJ (2018). A highly porous copper electrocatalyst for carbon dioxide reduction. Adv. Mater..

[13] Dinh C-T (2018). CO_2_ electroreduction to ethylene via hydroxide-mediated copper catalysis at an abrupt interface. Science.

[14] Ma W (2020). Electrocatalytic reduction of CO_2_ to ethylene and ethanol through hydrogen-assisted C–C coupling over fluorine-modified copper. Nat. Catal..

[15] Dinh C-T, García de Arquer FP, Sinton D, Sargent EH (2018). High rate, selective, and stable electroreduction of CO_2_ to CO in basic and neutral media. ACS Energy Lett..

[16] García de Arquer FP (2020). CO_2_ electrolysis to multicarbon products at activities greater than 1 A cm^−2^. Science.

[17] Kas R (2016). Three-dimensional porous hollow fibre copper electrodes for efficient and high-rate electrochemical carbon dioxide reduction. Nat. Commun..

[18] Zhu C (2021). Copper hollow fiber electrode for efficient CO_2_ electroreduction. J. Power Sources.

[19] Rabiee H (2020). Tuning the product selectivity of the Cu hollow Fiber gas diffusion electrode for efficient CO_2_ reduction to formate by controlled surface Sn electrodeposition. ACS Appl. Mater. Interfaces.

[20] Hummadi KK, Sustronk A, Kas R, Benes N, Mul G (2021). Optimizing temperature treatment of copper hollow fibers for the electrochemical reduction of CO_2_ to CO. Catalysts.

[21] Bell D (2020). Tubular hollow fibre electrodes for CO_2_ reduction made from copper aluminum alloy with drastically increased intrinsic porosity. Electrochem. Commun..

[22] Lu Q (2014). A selective and efficient electrocatalyst for carbon dioxide reduction. Nat. Commun..

[23] Ma M, Trześniewski BJ, Xie J, Smith WA (2016). Selective and efficient reduction of carbon dioxide to carbon monoxide on oxide-derived nanostructured silver electrocatalysts. Angew. Chem. Int. Ed..

[24] Liu SB (2017). Shape-dependent electrocatalytic reduction of CO_2_ to CO on triangular sliver nanoplates. J. Am. Chem. Soc..

[25] Kim D (2020). Selective CO_2_ electrocatalysis at the pseudocapacitive nanoparticle/ordered-ligand interlayer. Nat. Energy.

[26] Moussallem I, Joerissen J, Kunz U, Pinnow S, Turek T (2008). Chlor-alkali electrolysis with oxygen depolarized cathodes: history, present status and future prospects. J. Appl. Electrochem..

[27] Karlsson RKB, Cornell A (2016). Selectivity between oxygen and chlorine evolution in the chlor-alkali and chlorate processes. Chem. Rev..

[28] Gupta N, Gattrell M, MacDougall B (2006). Calculation for the cathode surface concentrations in the electrochemical reduction of CO_2_ in KHCO_3_ solutions. J. Appl. Electrochem..

[29] Kim B, Ma S, Jhong HRM, Kenis PJ (2015). Influence of dilute feed and pH on electrochemical reduction of CO_2_ to CO on Ag in a continuous flow electrolyzer. Electrochim. Acta.

[30] Burdyny T, Smith WA (2019). CO_2_ reduction on gas-diffusion electrodes and why catalytic performance must be assessed at commercially-relevant conditions. Energy Environ. Sci..

[31] Li J (2018). Efficient electrocatalytic CO_2_ reduction on a three-phase interface. Nat. Catal..

[32] Tian Y, Wang ZY, Wang LQ (2021). Hollow fibers: from fabrication to applications. Chem. Commun..

[33] Rosen J (2015). Mechanistic insights into the electrochemical reduction of CO_2_ to CO on nanostructured Ag surfaces. ACS Catal..

[34] Dunwell M, Luc W, Yan Y, Jiao F, Xu B (2018). Understanding surface-mediated electrochemical reactions: CO_2_ reduction and beyond. ACS Catal..

[35] Gu J, Hsu CS, Bai L, Chen HM, Hu X (2019). Atomically dispersed Fe(^3+^) sites catalyze efficient CO_2_ electroreduction to CO. Science.

[36] Ma M, Liu K, Shen J, Kas R, Smith WA (2018). In situ fabrication and reactivation of highly selective and stable Ag catalysts for electrochemical CO_2_ conversion. ACS Energy Lett..

[37] Shan WY (2020). In situ surface-enhanced Raman spectroscopic evidence on the origin of selectivity in CO_2_ electrocatalytic reduction. ACS Nano.

[38] Yan XP (2021). Efficient electroreduction of CO_2_ to C_2+_ products on CeO_2_ modified CuO. Chem. Sci..

[39] Rudolph WW, Irmer G, Königsberger E (2008). Speciation studies in aqueous HCO_3_^−^–CO_3_^2−^ solutions. A combined Raman spectroscopic and thermodynamic study. Dalton Trans..

[40] Chen XY (2021). Electrochemical CO_2_-to-ethylene conversion on polyamine-incorporated Cu electrodes. Nat. Catal..

[41] An H (2021). Sub-second time-resolved surface-enhanced Raman spectroscopy reveals dynamic CO intermediates during electrochemical CO_2_ reduction on copper. Angew. Chem. Int. Ed..

[42] Iwasita T, Rodes A, Pastor E (1995). Vibrational spectroscopy of carbonate adsorbed on Pt (111) and Pt (110) single-crystal electrodes. J. Electroanal. Chem..

[43] Chernyshova IV, Somasundaran P, Ponnurangam S (2018). On the origin of the elusive first intermediate of CO_2_ electroreduction. Proc. Nat. Acad. Sci. USA.

[44] Moradzaman M, Mul G (2021). In situ Raman study of potential-dependent surface adsorbed carbonate, CO, OH, and C species on Cu electrodes during electrochemical reduction of CO_2_. ChemElectroChem.

