# Supplementary material for: Hierarchical micro/nanostructured silver hollow fiber boosts electroreduction of carbon dioxide
Source: Nat Commun. 2022 Jun 2;13:3080. doi: 10.1038/s41467-022-30733-6 (PMC9163090; doi:10.1038/s41467-022-30733-6)
Supplement: Supplementary file 1 — Supplementary information [file 41467_2022_30733_MOESM1_ESM.pdf]

Supplementary Information for

Hierarchical micro/nanostructured silver hollow fiber boosts electroreduction of carbon dioxide

Shoujie Li, Wei Chen*, Xiao Dong, Chang Zhu, Aohui Chen, Yanfang Song, Guihua Li, Wei Wei*, Yuhan Sun*

Correspondence to: chenw@sari.ac.cn; weiwei@sari.ac.cn; sunyh@sari.ac.cn

[1]Low-Carbon Conversion Science and Engineering Center, Shanghai Advanced Research Institute, Chinese Academy of Sciences, Shanghai 201210, PR China.

[2]University of Chinese Academy of Sciences, Beijing 100049, PR China.

[3]School of Physical Science and Technology, ShanghaiTech University, Shanghai 201203, PR China.

Contents

Supplementary Methods……………………………………………………………………….....S2
Preparations ……………………………………………………………………………….......S2
Gas Permeation Tests………………………………………………………………….......S3
CO_2 Electroreduction and Product Quantifications…………………………………….......S3
Supplementary Figure 1…………………………………………………………………………S6
Supplementary Figure 2…………………………………………………………………………S7
Supplementary Figure 3…………………………………………………………………………S9
Supplementary Figure 4………………………………………………………………………S11
Supplementary Figure 5……………………..…………………………………………………S13
Supplementary Figure 6……………………..…………………………………………………S14
Supplementary Figure 7…………………..……………………………………………………S15
Supplementary Figure 8…………………..……………………………………………………S16
Supplementary Figure 9…………………..…………………………………………………....S17
Supplementary Figure 10………………..………………………………………………….....S18
Supplementary Figure 11………………..………………………………………………….....S19
Supplementary Figure 12………………..………………………………………………….....S20
Supplementary Figure 13……………..………………………………………………….........S21
Supplementary Figure 14……………..…………………………………………………….....S23
Supplementary Figure 15………………..………………………………………………….....S24
Supplementary Figure 16……………..……………………………………………………....S25
Supplementary Figure 17……………..……………………………………………………....S27
Supplementary Figure 18……………..……………………………………………………....S28
Supplementary Figure 19……………..……………………………………………………....S29
Supplementary Figure 20………………..………………………………………………….....S31
Supplementary Figure 21………………..………………………………………………….....S32
Supplementary Figure 22………………..………………………………………………….....S34
Supplementary Figure 23………………..………………………………………………….....S35
Supplementary Table 1 ……………………………………………………………………........S36
Supplementary Table 2 …………………………………………………………………...….S41
Supplementary References……………………………………………………………...….............S42

Supplementary Methods

Preparations

Synthesis of Ag hollow fiber (Ag HF)

Ag HF was fabricated by a combined phase-inversion/sintering process (Supplementary Fig. 1). Briefly, commercially available polyetherimide (PEI, 24 g) was added to N-Methyl-2-pyrrolidone (NMP, 96 g), followed by ultrasonic treatment for 1 h to obtain a homogeneous and transparent solution. Then Ag powder (80 g) was added to the above solution. The as-obtained mixture was further treated by the planetary ball-milling (using 250 mL zirconia jar and ϕ5 mm zirconia balls) with 300 rpm for 24 h to form a uniform slurry. After cooling to room temperature, the slurry was vacuumized (1 mbar) for 5 h to remove bubbles and then to obtain a casting solution. Next, the casting solution was extruded through a spinning machine and shaped in a water bath via the phase-inversion process. After spinning, the as-formed tubes were kept in a water bath for 24 h to eliminate the solvent completely, followed by stretching and drying in the ambient conditions with a humidity of ~28 % for 48 h to obtain a green body. The green body was cut into appropriate lengths and then calcinated in an air flow (100 mL·min^{-1}) at 600 °C (heating rate: 1 °C·min^{-1}) for 6 h to remove PEI. After being naturally cooled to room temperature, the calcined green body was then reduced in a 5% H_2 (argon balance) flow (100 mL·min^{-1}) at 300 °C (heating rate: 1 °C·min^{-1}) for 3 h to obtain Ag HF.

The Ag HF array comprising ten Ag HF tubes was used as the working electrode (Supplementary Fig. 11a). Each Ag HF tube, with an exposed length of 3 cm (average outer diameter: 425 µm, *vide infra*), was stuck into a copper tube using conductive silver adhesive for electrical contact, while the ends of the Ag HF tubes as well as the joints between the Ag HF and copper tube were sealed and covered with gas-tight and nonconductive epoxy. After drying at room temperature for 12 h, a working Ag HF electrode was obtained with an exposed geometric area of 4 cm^2 ($S=n\pi D_{out}L=10\times3.14159\times425\times0.0001\times3=4$ cm^2, where S is the electrode area, n is the number of hollow fiber tubes, D_{out} is the outer diameter of hollow fiber, and L is the length of hollow fiber) and a silver loading of 29 ± 1 mg·cm^{-2}.

Syntheses of electrooxidized Ag HF and activated Ag HF

Activated Ag HF was synthesized from Ag HF by electrochemical redox activation treatments. Typically, the Ag HF electrode was subjected to oxidation and reduction treatments on a Biologic VMP3 potentiostat using a three-electrode system in a gas-tight two-compartment electrolysis cell containing a Nafion 117 membrane as the separator, a KCl-saturated Ag/AgCl reference electrode and a platinum mesh (3 cm × 3 cm) counter electrode. The electrolyte solution was CO_2-saturated 0.5 M $KHCO_3$, and the CO_2 flow rate was kept at 10 mL·min^{-1}. Prior to the experiments, the electrolysis cell was vacuumized and then purged with CO_2 for 30 min. The Ag HF electrode was electrochemically oxidized at a fixed potential of 2.0 V (*vs.* Ag/AgCl) for 240 s to obtain electrooxidized Ag HF. Subsequently, the electrooxidized Ag HF was reduced at a fixed potential of -0.50 V (*vs.* Ag/AgCl) for 600 s to obtain activated Ag HF. The activated Ag HF electrode possessed the same exposed geometric area of 4 cm^2 ($S=n\pi D_{out}L=10\times3.14159\times425\times0.0001\times3=4$ cm^2, where S is the electrode area, n is the number of hollow fiber tubes, D_{out} is the outer diameter of hollow fiber, and L is the length of hollow fiber) and a silver loading of 29 ± 1 mg·cm^{-2}. The electrochemical oxidation reaction and reduction reaction obeyed **Equation**s (**1**) and (**2**), respectively.

$$2Ag + 2H_2O + HCO_3^- \rightarrow Ag_2CO_3 + 6e^- + O_2\uparrow + 5H^+ \qquad \text{Eq. (1)}$$

$$Ag_2CO_3 + 5H^+ + 6e^- \rightarrow 2Ag + HCO_3^- + 2H_2\uparrow \qquad \text{Eq. (2)}$$

In addition, the Ag HF electrode was also treated with other different electrochemical oxidation times (30 s, 60 s, 120 s, 180 s, 300 s) at the fixed potential of 2.0 V (*vs.* Ag/AgCl), followed by the same electrochemical reduction at the fixed potential of -0.5 V (*vs.* Ag/AgCl) with the fixed reduction time (600 s) to obtain a series of activated Ag HF electrodes. After an overall comparison of the CO_2 electroreduction performances, the activated Ag HF electrode obtained from Ag HF-redox-240s had the best electrocatalytic activity (Supplementary Figs. 2–4). Therefore, the activated Ag HF electrode in the main text and Supplementary Information refers to the electrode that underwent 240 s of oxidation and 600 s of reduction unless otherwise stated.

Synthesis of activated Ag foil

Ag foil and activated Ag foil working electrodes were used as references. A piece of Ag foil was ultrasonically cleaned in acetone and ethanol, and after drying in air, the side and back of the Ag foil were sealed with epoxy to obtain a Ag foil electrode with an exposure geometric area of 2 cm × 2 cm. And the synthesis procedure for activated Ag foil was the same as that of activated Ag HF. Thus, the activated Ag foil electrode also possessed the same exposed geometric area of 4 cm^2.

<u>Gas Permeation Tests</u>

Gas permeation tests were performed with a custom gas permeability device that could record the permeability of H_2, He, CH_4, N_2 and CO_2 through the hollow fiber under different transmembrane pressure drops. According to the Yasuda-Tsai equations[1,2], the permeability coefficient K of porous hollow fiber can be expressed as follows:

$$\boldsymbol{K = K_0 + B_0 P \eta^{-1}} \qquad \text{Eq. (3)}$$

where K_0 is the Knudsen permeability coefficient, B_0 is the geometric factor of hollow fiber wall, P is the mean pressure on both sides of the fiber, and η is the viscosity of N_2 gas. The values of K_0 and B_0 can be calculated from the slope and intercept of the plot of K to P. The effective porosity (ε/q^2) can also be estimated by the Knudsen permeability coefficient K_0 in **Equation (4)**:

$$\boldsymbol{{K_0}^2 = \left(\frac{3.2}{3}\right)\left(\frac{\varepsilon}{q^2}\right)\frac{16B_0}{3}\frac{4RT}{\pi M}} \qquad \text{Eq. (4)}$$

where ε is the porosity, q is the tortuosity factor, R is the gas constant, T is the temperature, and M is the molecular weight of the gas.

<u>CO_2 Electroreduction and Product Quantifications</u>

The potentiostatic electroreductions of CO_2 over all electrodes were performed at ambient temperature and pressure on the Biologic VMP3 potentiostat using the gas-tight electrolysis cell, which comprised two symmetrical compartments made of quartz glass with an inner height of 5.0 cm, an inner length of 5.0 cm and an inner width of 1.5 cm (Supplementary Figs. 11b–d). The cathodic and anodic compartments were separated by a Nafion 117 membrane, and the electrolysis cell was equipped with a KCl-saturated Ag/AgCl reference electrode in the cathodic compartment and a platinum mesh counter electrode in the anodic compartment.

CO_2-saturated $KHCO_3$ aqueous solutions with different concentrations were used as the electrolyte solutions, which were cycled in both the cathodic and anodic compartments at a fixed flow rate of 20 $mL \cdot min^{-1}$ by using two identical peristaltic pumps (Jihpump BT-50EA 153YX). Prior to the experiments, the electrolysis cell was vacuumized and then purged with CO_2 for 30 min.

Under the similar electrolysis conditions, CO_2 flow rate of lower than 10 mL·min^{-1} resulted in very low CO faradaic efficiencies and CO_2 conversion rates. While both the CO faradaic efficiency and CO_2 conversion rate increased rapidly when CO_2 flow rate was larger than 10 mL·min^{-1}, and up to 60 mL·min^{-1}. Further increasing the CO_2 flow rate to more than 60 mL·min^{-1} led to the slow increase of CO faradaic efficiencies and the rapid decrease of CO_2 conversion rate. In order to obtain both appropriate CO faradaic efficiency and CO_2 conversion rate, the CO_2 flow rate was fixed at 60 mL·min^{-1} during CO_2 electroreduction unless otherwise stated (Supplementary Fig. 13). In the situations with very large currents (>400 mA), the Biologic VMP3 potentiostat was connected to a VMP3 booster chassis with an option of 10 A current.

The retention time values of CO_2 through the different electrodes have been estimated obeying the equations below based on their structure and porosity. That is the CO_2 retention times through Ag HF and activated Ag HF are 31.6 and 30.5 ms, respectively. The retention time of the electrodes was calculated as follows:

$$\tau = (\boldsymbol{V}_{\mathbf{wall}} + \boldsymbol{V}_{\mathbf{in}})/\boldsymbol{v}_{\boldsymbol{CO_2}} \qquad \text{Eq. } (\mathbf{5})$$

$$\boldsymbol{V}_{\mathbf{wall}} = \boldsymbol{n}\left(\frac{\boldsymbol{\varepsilon}}{\boldsymbol{q}^2}\right)\pi\left(\left(\frac{\boldsymbol{D}_{\mathbf{out}}}{\mathbf{2}}\right)^{\mathbf{2}} - \left(\frac{\boldsymbol{D}_{\mathbf{in}}}{\mathbf{2}}\right)^{\mathbf{2}}\right)\boldsymbol{L} \qquad \text{Eq. } (\mathbf{6})$$

$$\boldsymbol{V}_{\mathbf{in}} = \boldsymbol{n}\pi\left(\frac{\boldsymbol{D}_{\mathbf{in}}}{\mathbf{2}}\right)^{\mathbf{2}}\boldsymbol{L} \qquad \text{Eq. } (\mathbf{7})$$

where τ is the retention time, V_{wall} is the pore volume of hollow fiber wall, V_{in} is the volume of hollow fiber inner channel, v_{CO2} is the flow rate of CO_2, n is the number of hollow fiber tubes, ε is the porosity, q is the tortuosity factor, D_{out} is the outer diameter of hollow fiber, D_{in} is the inner diameter of hollow fiber, and L is the length of hollow fiber.

The theoretical limits of CO partial current density, i.e., $\boldsymbol{j}_{\mathbf{CO,lim(gas)}}$ and $\boldsymbol{j}_{\mathbf{CO,lim(sol)}}$ were calculated by the following two **Equations** (**8**) and (**9**), respectively. The former $\boldsymbol{j}_{\mathbf{CO,lim(gas)}}$ is the theoretical limit of CO partial current density with all gas-phase CO_2 molecules input into the electrolysis cell were electroreduced to CO. The latter $\boldsymbol{j}_{\mathbf{CO,lim(sol)}}$ is the theoretical limit of CO partial current density with all CO_2 molecules dissolved in the electrolyte solution were electroreduced to CO[3–5].

$$\boldsymbol{j}_{\boldsymbol{CO,lim(gas)}} = \frac{\boldsymbol{\alpha F}}{\mathbf{S}}\boldsymbol{k}_{\mathbf{m}}\frac{\boldsymbol{v}_{\boldsymbol{CO_2}}}{\boldsymbol{V}_{\mathbf{m}}} \qquad \text{Eq. } (\mathbf{8})$$

$$\boldsymbol{j}_{\boldsymbol{CO,lim(sol)}} = \boldsymbol{\alpha F D}\frac{\boldsymbol{C}}{\boldsymbol{\delta}} \qquad \text{Eq. } (\mathbf{9})$$

where α is the number of transferred electrons for producing CO, F is the Faraday constant (96485 C·mol^{-1}), S is the electrode area (4 cm^2), k_m is the mass transfer coefficient (k_m =1 to obtain the value of $\boldsymbol{j}_{\mathbf{CO,lim(gas)}}$), v_{CO2} is the flow rate of CO_2, V_m is the gas mole volume (24.5 L·mol^{-1} at 25 °C, 101.325 kPa), D is the diffusion coefficient of CO_2 (2.02 × 10^{-9} m^2·s^{-1}), c is the saturated bulk concentration of CO_2 (34 mol·m^{-3} at 25 °C, 101.325 kPa), δ is the diffusion layer thickness, which is estimated to be 14.0 μm using the rotating disk electrode model with the Levich equation[3].

The experimental CO_2 conversion rate was determined in accordance with the following equation:

$$\boldsymbol{CO_2\ conversion\,rate} = \frac{\boldsymbol{CO\ produced}}{\boldsymbol{CO_2\ inputted}} \times \mathbf{100\%} \qquad \text{Eq. } (\mathbf{10})$$

The theoretical limit of CO_2 conversion rate was calculated using **Equation** (**11**) below:

$$\boldsymbol{Con}_{\boldsymbol{CO2,lim}} = \frac{\boldsymbol{equivalent\ CO\ amount\ based\ on\ j_{CO,lim(gas)}}}{\boldsymbol{CO_2\ inputted}} \qquad \text{Eq. } (\mathbf{11})$$

For the long-term performance test of CO_2 electroreduction, the fixed potential of -0.83 V (*vs.* RHE) was applied to the activated Ag HF electrode. The electrolyte was CO_2-saturated 1.5

M $KHCO_3$ and the CO_2 flow rate was kept at 60 mL·min^{-1}. The catholyte and anolyte were cycled at a flow rate of 20 mL·min^{-1}, accompanied by the supplement of ultrapure water to maintain a constant concentration of 1.5 M $KHCO_3$. The exhaust from the cathodic compartment was measured by the online gas chromatography (GC) during the whole 170-hour test.

All the current densities in the main text and Supplementary Information were based on the electrode geometric area.

Gas-phase products from the cathodic compartment were directly vented into a gas chromatograph (GC-2014, Shimadzu) equipped with a Shincarbon ST80/100 column and a Porapak-Q80/100 column using a flame ionization detector (FID) and a thermal conductivity detector (TCD) during the electroreduction tests and online analysis. A GC run was initiated every 15 min. To ensure the accuracy of the gas-phase products, when the CO concentration in the exhaust was lower than 10%, the FID detector was used for CO quantification; when the CO concentration in the exhaust was higher than 10%, the TCD was used as the main detector of CO, and the FID was used as the auxiliary detector. The TCD quantification was used for H_2 quantification. All faradaic efficiencies reported were based on at least five different runs. High purity argon (99.999%) was used as the carrier gas of GC. In all the potentiostat electrolysis tests, H_2 and CO were the only gas-phase products, and their faradaic efficiencies were calculated as follows:

$$\boldsymbol{FE} = \frac{\boldsymbol{C_{product}} \times \mathbf{10^{-6}} \times \boldsymbol{v_{CO_2}} \times \mathbf{10^{-3}} \times \boldsymbol{t} \times \boldsymbol{\alpha} \times \boldsymbol{F}}{\boldsymbol{V_m} \times \boldsymbol{Q}} \times \mathbf{100\%} \qquad \text{Eq. } (\mathbf{12})$$

where C_{product} is the concentration of the gas-phase products (ppm), v_{CO_2} is the flow rate of CO_2 (60 mL·min^{-1}), t is the reaction time, α is the number of transferred electrons for producing CO or H_2, F is the Faraday constant, V_m is the gas mole volume, and Q is the total quantity of electric charge.

The possible liquid-phase products from the cathodic compartment after potentiostatic electrolysis for 1 h were analyzed using an offline GC-2014 (Shimadzu) equipped with a headspace injector and an OVI-G43 capillary column (Supelco, USA). There were no liquid-phase products detected by offline GC. The postreaction catholyte solution was also further analyzed by using a 600 MHz nuclear magnetic resonance (NMR) spectrometer (Bruker). After an hour of electrolysis, an aliquot of catholyte solution (0.5 mL) was mixed with 0.1 mL of DSS (6 mM) and 0.1 mL of D_2O, which were used as internal standards. No liquid-phase product was detected by NMR.

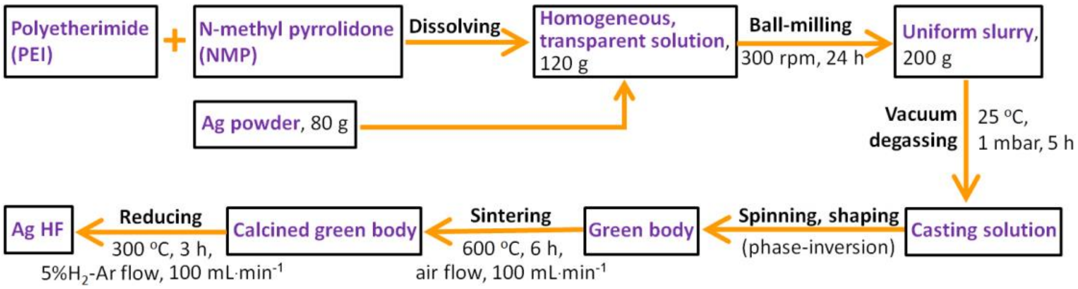

Supplementary Figure 1 | Diagram of the detailed fabrication procedures of Ag HF.

The diagram for the detailed fabrication procedures of Ag HF is shown in Supplementary Fig. 1, and the related experimental descriptions can be found in the Materials and Methods section. The whole fabrication of Ag HF involved only basic laboratory apparatuses under relatively mild conditions. Notably, the above fabrication process produced one batch of Ag HF with a total length of more than 180 meters, demonstrating its high potential for scalable applications.

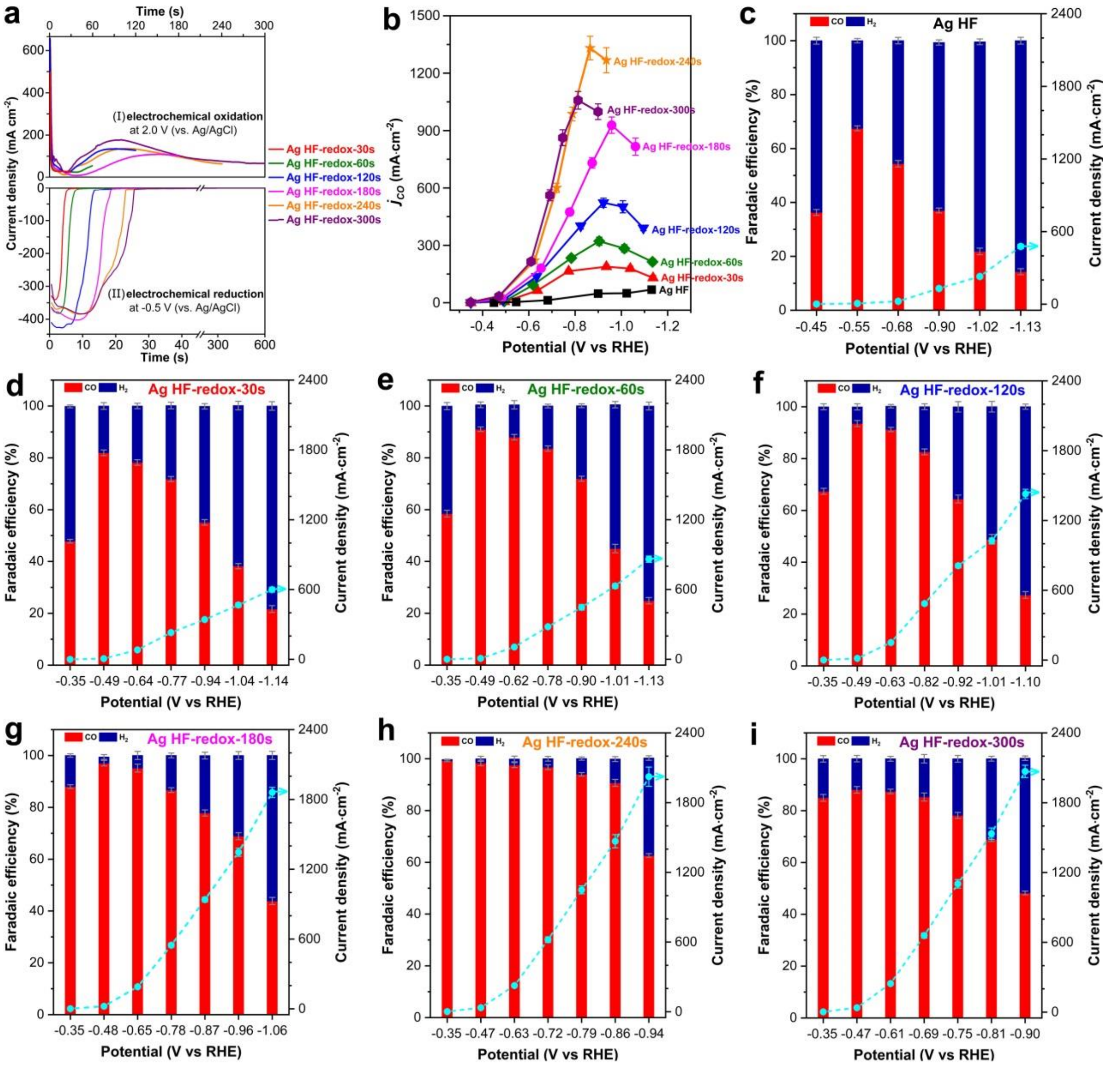

Supplementary Figure 2 | Electrochemical redox activation treatments of the Ag HF electrode to obtain different activated Ag HF electrodes, and their CO_2 electroreduction performance. a, Electrochemical oxidation and reduction current density curves during different oxidation treatments (from 30 s to 300 s) at 2.0 V (*vs.* Ag/AgCl) in 0.5 M $KHCO_3$, and the subsequent respective reduction treatments for 600 s at -0.5 V (*vs.* Ag/AgCl) in the same electrolyte solution. **b,** Comparison of the CO partial current densities of the Ag HF electrode and the different activated Ag HF electrodes. The CO and H_2 faradaic efficiencies and total current densities over **c,** the Ag HF electrode and **d**–**i,** the different activated Ag HF electrodes in CO_2-saturated 1.5 M $KHCO_3$. Error bars in **b-i** were obtained from the average of six individual tests.

The Ag HF electrode was subjected to different oxidation treatments (30 s, 60 s, 120 s, 180 s, 240 s and 300 s) and subsequent respective reduction treatments for 600 s to obtain a series of activated Ag HF electrodes (see the aforementioned Preparations section for details), denoted as Ag HF-redox-30s, Ag HF-redox-60s, Ag HF-redox-120s, Ag HF-redox-180s, Ag HF-redox-240s,

and Ag HF-redox-300s, respectively. From the oxidation and reduction current density-time curves of these activated Ag HF electrodes (Supplementary Fig. 2a), the amounts of accumulated charge at different oxidation times were proportional to those in the corresponding reduction stages, implying the redox reactions highly obeyed aforementioned **Equation**s (**1**) and (**2**), respectively.

The comparison of CO_2 electroreduction performance over the Ag HF electrode and all the activated Ag HF electrodes is shown in Supplementary Fig. 2b, and their detailed CO and H_2 faradaic efficiencies as well as the total current densities are also presented in Supplementary Figs. 2c–i. One can see that the CO partial current density showed obvious superiority at the more negative potentials with increasing oxidation time. And the Ag HF-redox-240s electrode delivered the highest j_{CO} among all the activated Ag HF electrodes. Therefore, the activated Ag HF electrode in the main text and Supplementary Information referred to the Ag HF-redox-240s electrode unless otherwise stated.

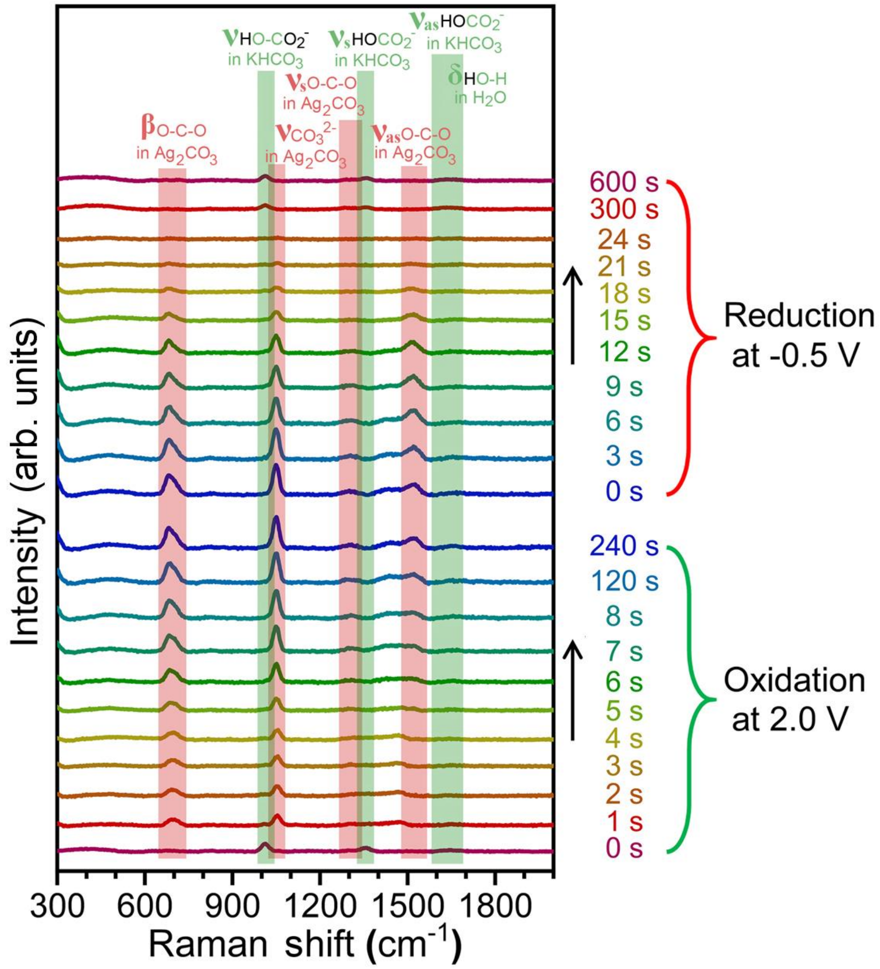

Supplementary Figure 3 | Time-resolved Raman spectra during the electrochemical oxidation and reduction treatments to obtain the activated Ag HF electrode, i.e., Ag HF-redox-240s.

The electrochemical oxidation and reduction treatments of Ag HF electrode to obtain the activated Ag HF electrode (Ag HF-redox-240s) were monitored by the time-resolved operando Raman spectroscopy. As shown in Supplementary Fig. 3, the Raman spectrum of Ag HF (at 0 s of the oxidation stage) showed the peaks at approximately 1012, 1360, 1603 and 1660 cm^{-1}, which were assigned to bicarbonate ions (HCO_3^-) adsorbed at the electrode surface as the ν_{HO-COO^-}, ν_{sHOCOO^-}, $\nu_{asHOCOO^-}$ and δ_{HO-H} (in H_2O) modes, respectively, according to previous reports [6,7]. Once the oxidation reaction occurred (as short as 1 s of the oxidation stage), new Raman peaks appeared at 682, 1047, 1296 and 1517 cm^{-1}, which could be assigned to the as-formed Ag_2CO_3 species as β_{O-C-O}, ν_{CO3}^{2-}, ν_{sO-C-O} and $\nu_{asO-C-O}$, respectively,[8,9] besides bicarbonate ion related peaks. With increasing oxidation time (2 s to 7 s), the intensities of the Ag_2CO_3-ralted peaks increased rapidly, and reached the maximum at 8 s during the oxidation stage. Further increasing the oxidation time (8 s to 240 s), the intensities of the Ag_2CO_3-related peaks remained constant. Combining the electrochemical oxidation current density curve (Supplementary Fig. 2) and the Raman observations, it was found that the oxidation reaction of Ag to Ag_2CO_3 occurred very quickly on Ag HF surface at the initial stage, and then expanded to the subsurface or

substrate to some degree, which was responsible for the constant peak intensities while keeping the oxidation current densities of 40–120 mA·cm^{-2} after 8 s.

As for the subsequent electrochemical reduction process, the intensities of the characteristic Ag_2CO_3 peaks faded rapidly, and became very weak at 21 s of the reduction stage. The Ag_2CO_3-related peaks were almost negligible at 24 s of the reduction stage and disappeared in the following reduction stage. Interestingly, the Raman observations on the electrochemical reduction were in consistence with the variation of the reduction current density curve (Supplementary Fig. 2). That is the reduction current density of Ag HF-redox-240s decreased to zero after 24 s at the potential of -0.50 V (vs. Ag/AgCl) (Supplementary Fig. 2). These time-resolved operando Raman results confirmed the transitions between Ag and Ag_2CO_3 compositions obeying **Equation**s (**1**) and (**2**) during the electrochemical redox activation treatments of Ag HF to obtain activated Ag HF.

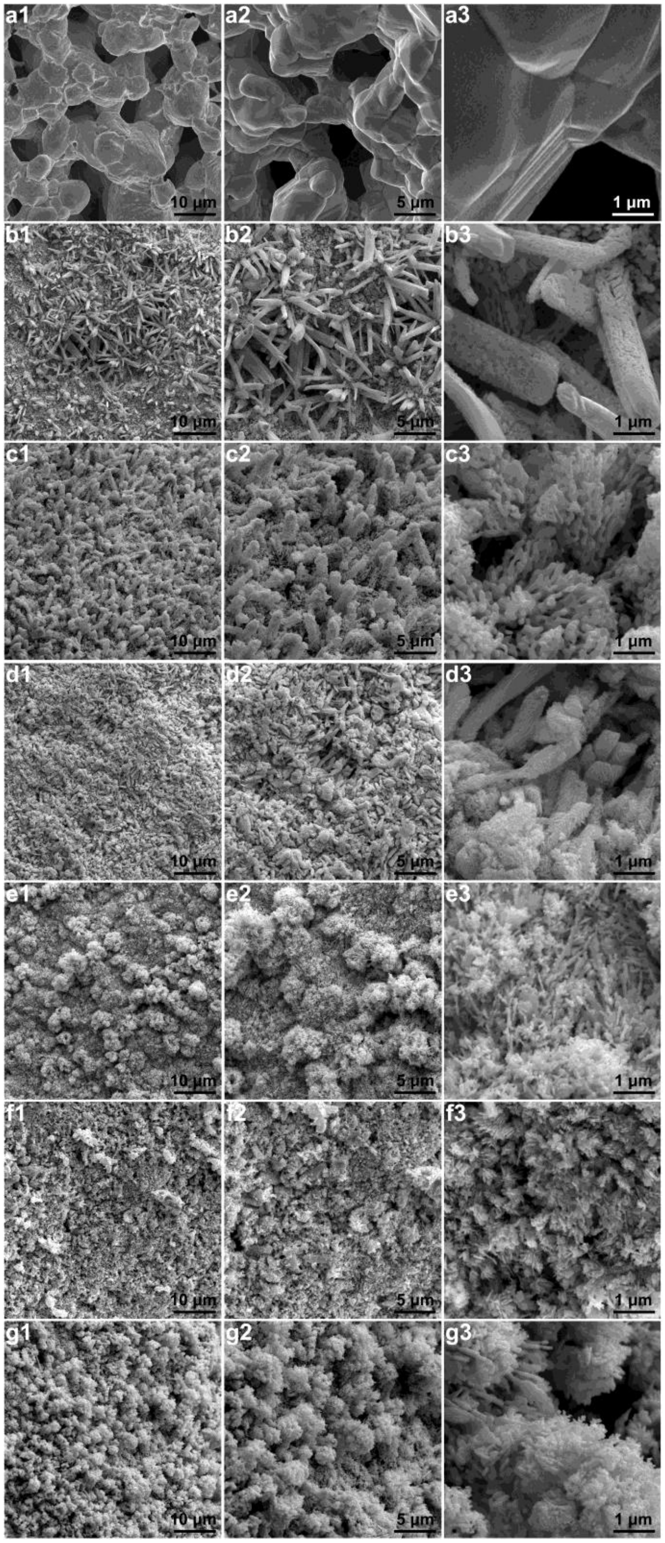

Supplementary Figure 4 | SEM images of the outer surface of a, Ag HF and b–g, different activated Ag HF electrodes with (a1-g1) low, (a2-g2) medium and (a3-g3) high magnifications, respectively. a, Ag HF, **b,** Ag HF-redox-30s, **c,** Ag HF-redox-60s, **d,** Ag HF-redox-120s, **e,** Ag HF-redox-180s, **f,** Ag HF-redox-240s, and **g,** Ag HF-redox-300s.

The outer surface morphologies of Ag HF and activated Ag HF with different redox pretreatments were investigated by SEM observations. As shown in the Supplementary Fig. 4a, the outer surface of Ag HF exhibited the abundant micrometer-sized pores with relatively smooth substrate. Once the electrochemical redox activation treatment even a slight oxidation as short as 30 s was applied, the outer surface morphology of the Ag HF-redox-30s changed greatly (Supplementary Fig. 4b). Numerous nanorods covered the outer surface of Ag HF-redox-30s, making the pores indistinct. With increasing oxidation time, the outer surfaces of activated Ag HF electrodes exhibited increasing surface coarseness and decreasing diameter of the as-formed nanorods (Supplementary Figs. 4c-g). Note that these nanorods partly ordered and gathered at the outer surface of Ag HF-redox-240s (Supplementary Fig. 4f). These hierarchical micro/nanostructures comprising partly ordered nanorods on the surface and micrometer-sized pores beneath the surface may maximized the three-phase reaction interfaces, resulting in the best electrocatalytic activity (Supplementary Fig. 2).

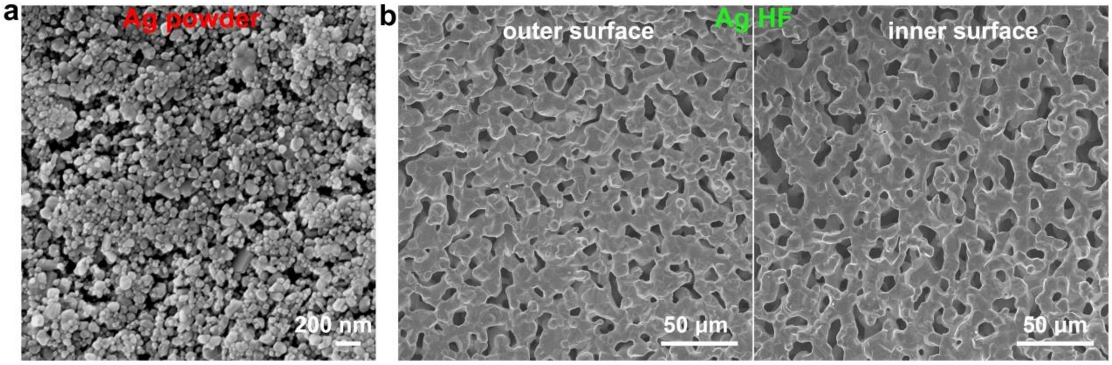

Supplementary Figure 5 | SEM images. **a,** Ag powder and **b,** the outer and inner surfaces of Ag HF.

The morphologies of pristine Ag powder and as-prepared Ag HF were investigated by SEM observations, as shown in Supplementary Fig. 5. The particles in the Ag powder were spherical with a relatively even particle size (~ 60 nm), but they appeared to aggregate (Supplementary Fig. 5a). In contrast, both the inner and outer surfaces of Ag HF showed a well-integrated substrate without spherical or granular particles (Supplementary Fig. 5b), implying that the silver particles were completely sintered and fused to form an integral hollow-fiber base during the fabrication process, thereby benefiting mechanical strength reinforcement and electron transfer. Both the outer and inner surfaces of Ag HF possessed abundant irregular micrometer-sized pores with a pore size of 5–20 μm.

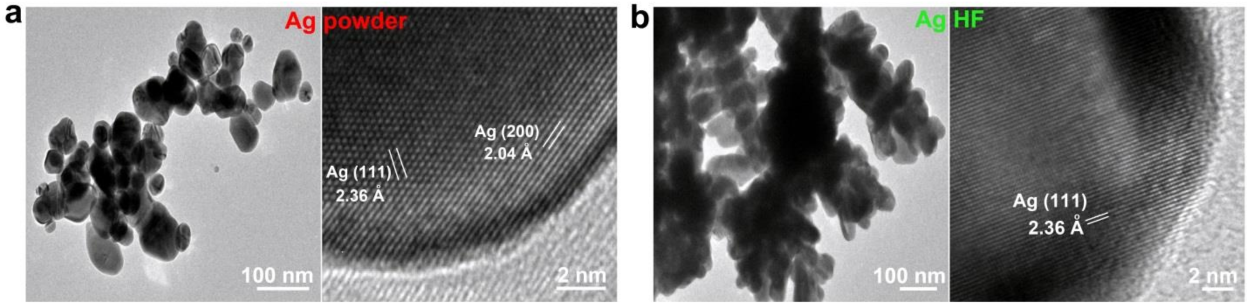

Supplementary Figure 6 | TEM images. a, Ag powder and **b,** Ag HF.

TEM was used to further investigate the morphologies of Ag powder and Ag HF, as shown in Supplementary Fig. 6. From the low-magnification TEM image, the particles in the Ag powder were spherical with a particle size range of 20–120 nm (Supplementary Fig. 6a), in agreement with the SEM observation (Supplementary Fig. 5a), while the fused nanorod-like particles obtained by scraping off the outer surface of Ag HF were presented (Supplementary Fig. 6b). Furthermore, the high-magnification TEM image showed that the lattice spacing of the Ag powder were 2.36 and 2.04 Å, corresponding to the (111) and (200) planes of metallic Ag, respectively (Supplementary Fig. 6a). Ag HF also presented a lattice spacing of 2.36 Å, corresponding to the Ag (111) plane (Supplementary Fig. 6b). These results indicate that Ag HF had the same metallic Ag phase as the pristine Ag powder.

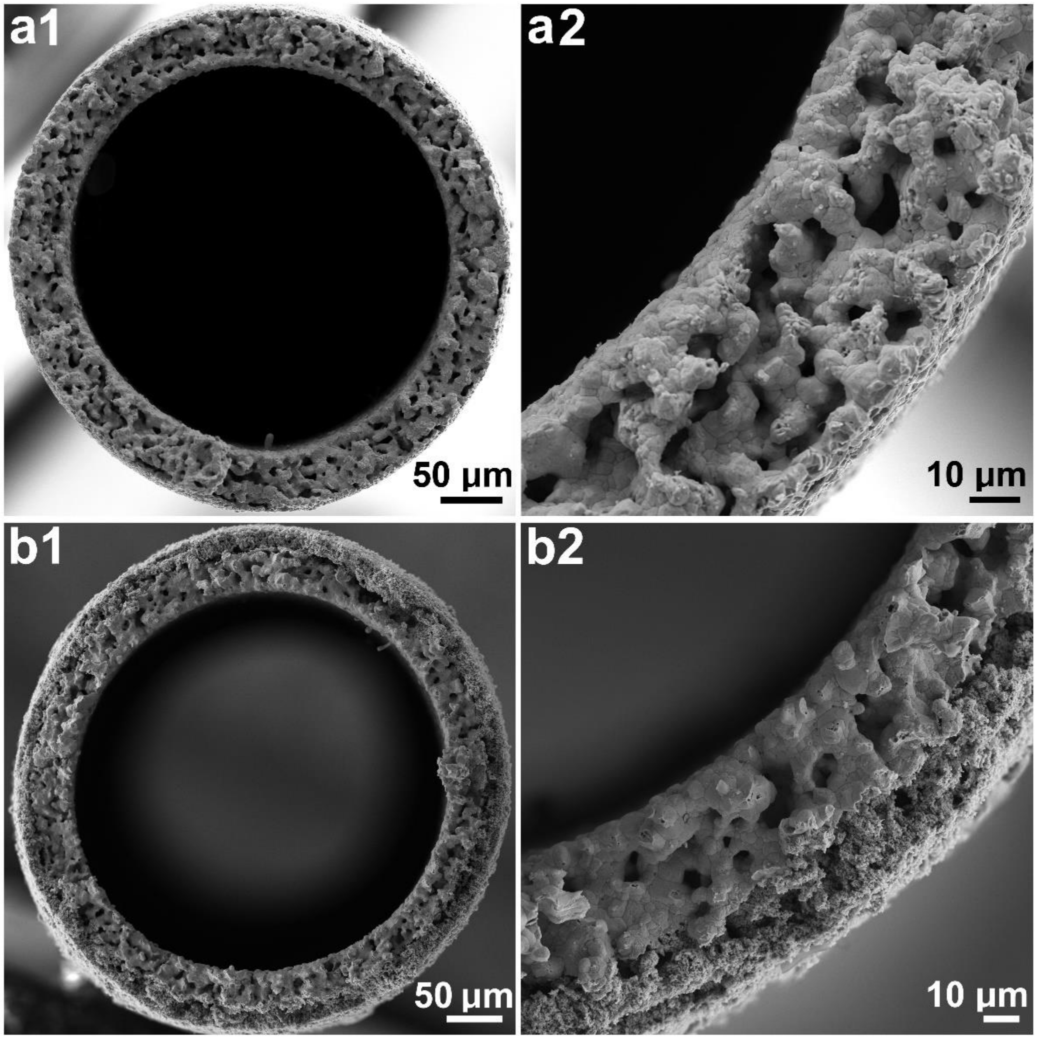

Supplementary Figure 7 | SEM images of the cross sections. a, Ag HF and **b,** activated Ag HF. **a1**, **b1**, Whole cross-section images. **a2**, **b2**, Magnified images of the local cross-section region. The cross-section SEM image of Ag HF in Supplementary Fig. 7a1, same as Fig. 2b in the main text.

The cross-section morphologies and pore structures of Ag HF and activated Ag HF were studied by SEM, as shown in Supplementary Fig. 7. Ag HF and activated Ag HF possessed similar wall thicknesses of ~50 µm and outer diameters of ~425 µm; additionally, their pores in the wall were interconnected (Supplementary Figs. 7a1, b1). Different from the symmetrical outer and inner regions of Ag HF (Supplementary Fig. 7a2), partly ordered nanorods gathered at the outer region of activated Ag HF, presenting a distinct configuration of hierarchical micro/nanostructures (Supplementary Fig. 7b2) derived from the electrochemical redox activation treatments. That is the CO_2 flow rate was kept at 10 mL·min^{-1} during the activation treatments and the redox reactions (referring to the aforementioned **Equation**s (**1**) and (**2**)) occurred only at the outer region of the hollow fiber wall.

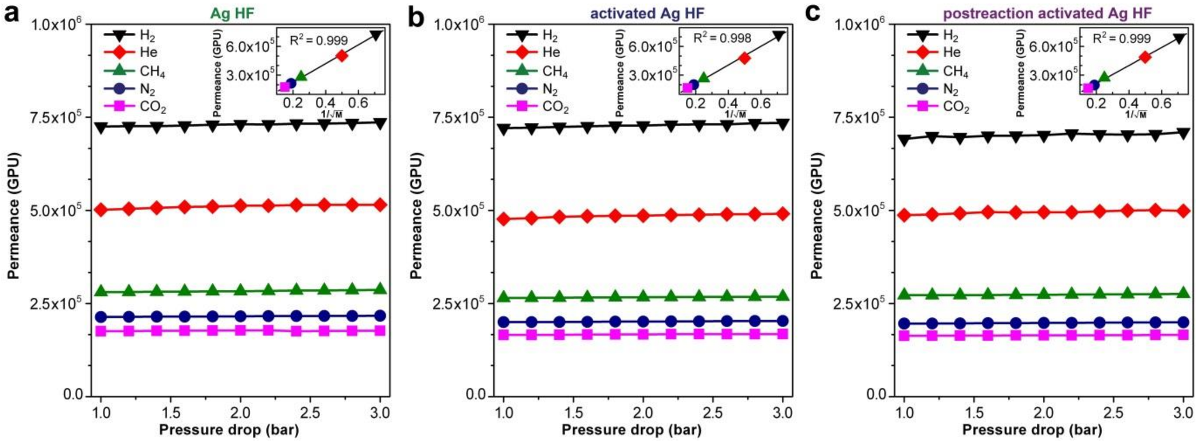

Supplementary Figure 8 | Gas permeances. a, Ag HF, **b,** activated Ag HF, and **c,** postreaction activated Ag HF at different transmembrane pressure drops. The insets show gas permeances at 1.0 bar plotted as a function of the inverse square root of the gas molecular weight. The permeances of activated Ag HF in Supplementary Fig. 8b, same as Fig. 2f in the main text.

Gas permeation was used to study the structural features of Ag HF and activated Ag HF before and after the reaction, as shown in Supplementary Fig. 8. All the gas permeances of H_2, He, CH_4, N_2 and CO_2 remained almost constant at different pressure drops, and the large permeance values indicated the high permeabilities of Ag HF and activated Ag HF before and after reaction. Moreover, the gas permeances were inversely proportional to the square roots of their molecular weight (the insets in Supplementary Fig. 8), implying that the gas transport mechanisms through all the hollow fibers were dominated by Knudsen diffusion[1,2]. Furthermore, the effective porosities of Ag HF and activated Ag HF, calculated by using the nitrogen permeance data according to **Equation** (**4**), were 38% and 32%, respectively. On the basis of the porosity and structure, the CO_2 retention times through Ag HF and activated Ag HF were 31.6 and 30.5 ms, respectively. In addition, the postreaction activated Ag HF possessed an effective porosity of 29% and a CO_2 retention time of 30.0 ms, close to those of activated Ag HF before the reaction, implying the structural stability due to the tough framework of activated Ag HF.

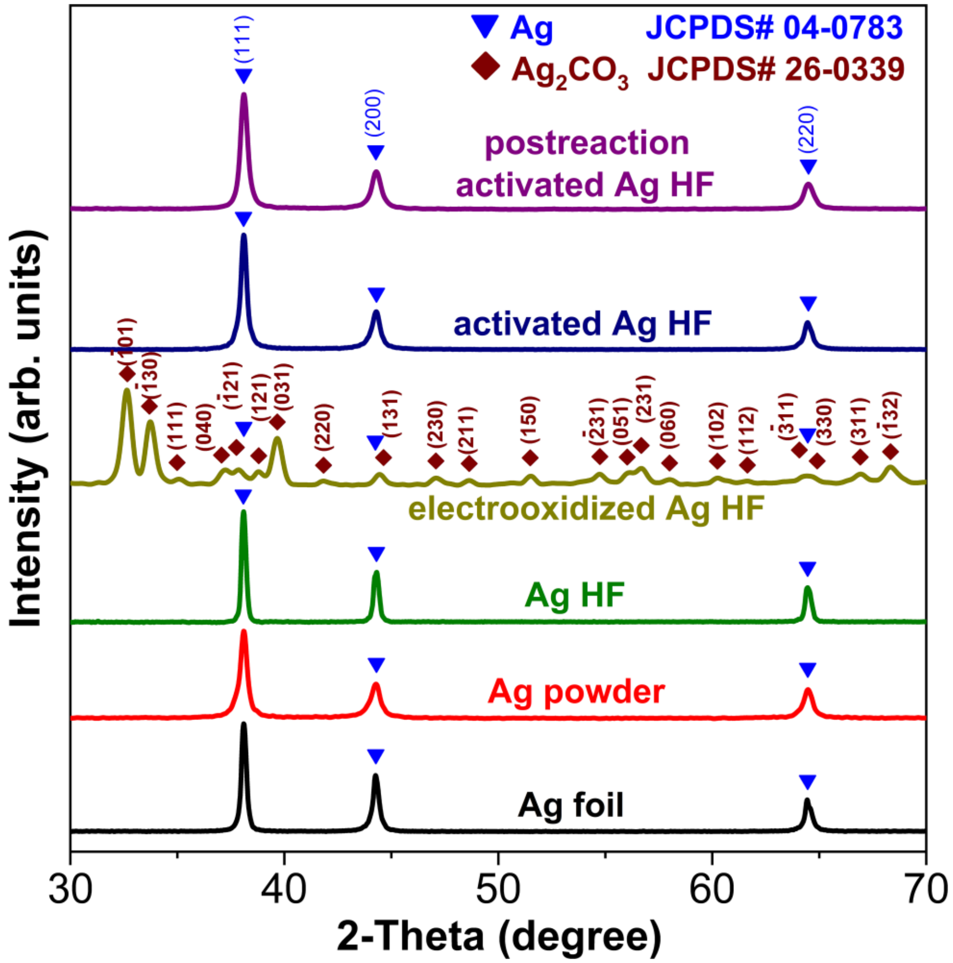

Supplementary Figure 9 | XRD patterns. Ag foil (black), Ag powder (red), Ag HF (olive), electrooxidized Ag HF (dark yellow), activated Ag HF (navy), and postreaction activated Ag HF (purple).

The phase compositions of all involved silver samples were studied by XRD. As shown in Supplementary Fig. 9, Ag foil, Ag powder and Ag HF showed three diffraction peaks at 38.1°, 44.3° and 64.4°, corresponding to the (111), (200), and (220) planes of metallic Ag (JCPDS no.04-0783), respectively. After electrochemical oxidation treatment, in addition to metallic Ag peaks, many new peaks appeared in electrooxidized Ag HF, which were assigned to the various planes of Ag_2CO_3 (JCPDS no. 26-0339). This result indicated that the electrochemical oxidation reaction obeyed **Equation** (**1**). By the subsequent electrochemical reduction treatment, all Ag_2CO_3 peaks converted back to metallic Ag peaks in activated Ag HF. In addition, the postreaction activated Ag HF also presented the same phase compositions as the activated Ag HF before the reaction. These XRD results indicated that all involved silver samples had only a metallic Ag phase with the same crystal form except for the electrooxidized Ag HF intermediate.

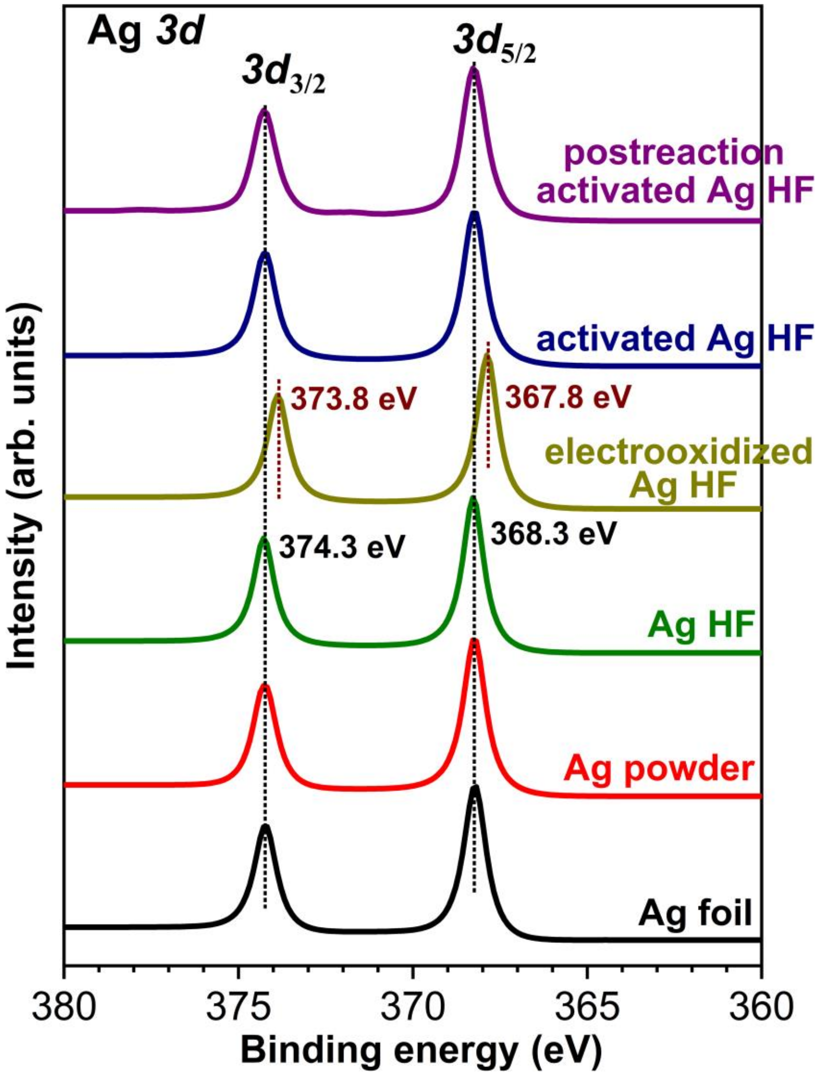

Supplementary Figure 10 | XPS spectra. The Ag *3d* level of Ag foil (black), Ag powder (red), Ag HF (olive), electrooxidized Ag HF (dark yellow), activated Ag HF (navy), and postreaction activated Ag HF (purple).

The surface compositions of all involved silver samples were studied by XPS. As shown in Supplementary Fig. 10, the Ag *3d* spectra in Ag foil, Ag powder and Ag HF showed the main Ag $3d_{5/2}$ and Ag $3d_{3/2}$ core peaks at binding energies of 368.3 and 374.3 eV, respectively, indicating metallic Ag^0 characteristics. Regarding electrooxidized Ag HF, the Ag $3d_{5/2}$ and Ag $3d_{3/2}$ peaks were at 367.8 and 373.8 eV, respectively, corresponding to the characteristic peaks of Ag_2CO_3 (referring to the standard spectrum of silver carbonate). This result implied that the surface of electrooxidized Ag HF was covered with Ag_2CO_3. Furthermore, the XPS spectra of activated Ag HF before and after the reaction suggested the same metallic Ag^0 surfaces, indicating the stable metallic Ag^0 active component during CO_2 electroreduction.

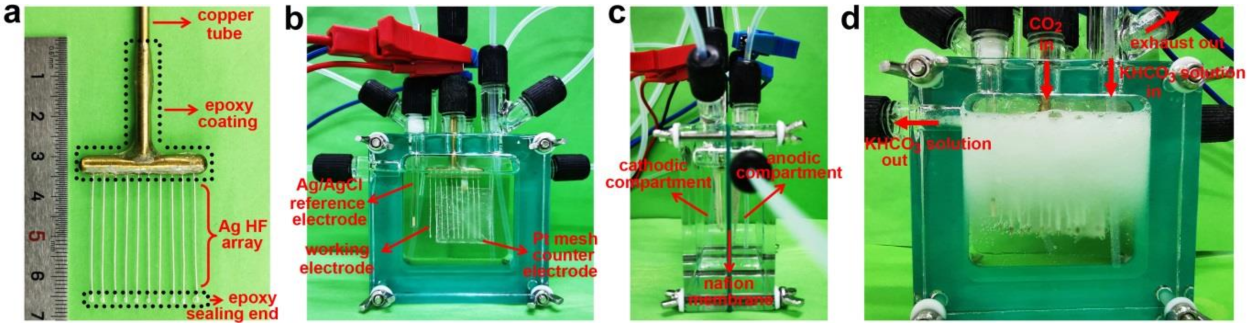

Supplementary Figure 11 | Optical images of the electrode and electrolysis cell. **a,** the working electrode of Ag HF, **b,** side view of the gas-tight two-compartment electrolysis cell, **c,** cross-section view of the electrolysis cell, and **d,** the electrolysis cell during CO_2 electroreduction. The arrows in **d** show the directions of the CO_2 flow, exhaust flow, and $KHCO_3$ solution flow.

Supplementary Figure 11 shows optical images of the Ag HF electrode and electrolysis cell in different views and states. The electrolysis cell comprised two symmetrical compartments made of quartz glass with an inner height of 5.0 cm, an inner length of 5.0 cm and an inner width of 1.5 cm. The Ag HF working electrode consisted of ten Ag HF tubes (i.e., Ag HF array), and each tube had an exposed length of 3 cm (Supplementary Fig. 11a). The working electrode and the Ag/AgCl reference electrode were in the cathodic compartment, and the Pt mesh counter electrode was in the anodic compartment (Supplementary Fig. 11b). The cathodic and anodic compartments were separated by a Nafion 117 membrane (Supplementary Fig. 11c). During CO_2 electroreduction, CO_2 penetrated through the wall of the activated Ag HF tubes via the copper tube, forming a large amount of bubbles (Supplementary Fig. 11d).

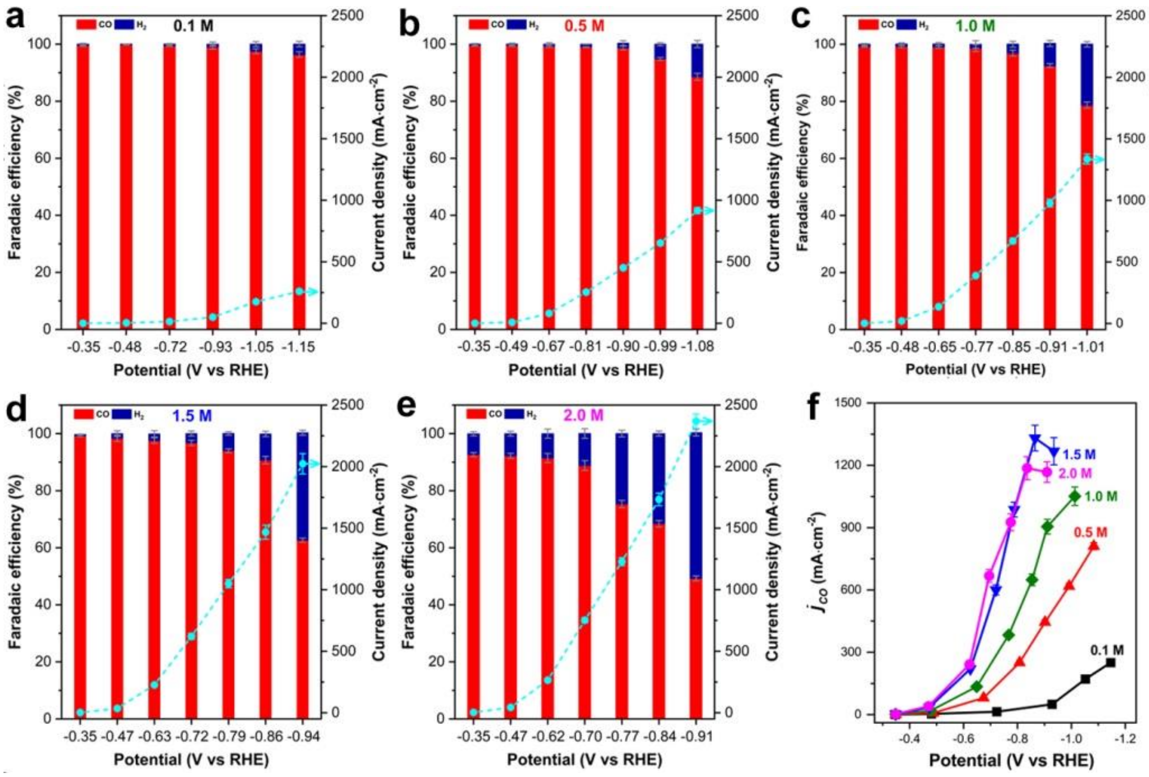

Supplementary Figure 12 | Electrocatalytic performance in different concentration solutions. CO and H_2 faradaic efficiencies, and total current densities over the activated Ag HF electrode in the potential range of -0.35 to -1.15 V in CO_2-saturated $KHCO_3$ solutions with different $KHCO_3$ concentrations of **a,** 0.1 M, **b,** 0.5 M, **c,** 1.0 M, **d,** 1.5 M, and **e,** 2.0 M. **f,** Comparison of the CO partial current densities in the different concentration solutions. Error bars in **a-f** were obtained from the average of six individual tests.

The detailed faradaic efficiencies of CO and H_2 as well as the total current densities of activated Ag HF in different $KHCO_3$ solutions are presented in Supplementary Figs. 12a–e. As the applied potential negatively shifted, the CO faradaic efficiencies decreased, while the H_2 faradaic efficiencies and the total current densities rapidly increased, especially at more negative potentials. Moreover, CO faradaic efficiencies in low concentration $KHCO_3$ solutions were higher than those in high concentration solutions at similar potentials. Furthermore, the CO partial current density showed superior in the relatively concentrated solutions with the best performance in 1.5 M $KHCO_3$ (Supplementary Fig. 12f). Therefore, CO_2-saturated 1.5 M $KHCO_3$ aqueous solution was chosen as the electrolyte solution for CO_2 electroreduction unless otherwise stated.

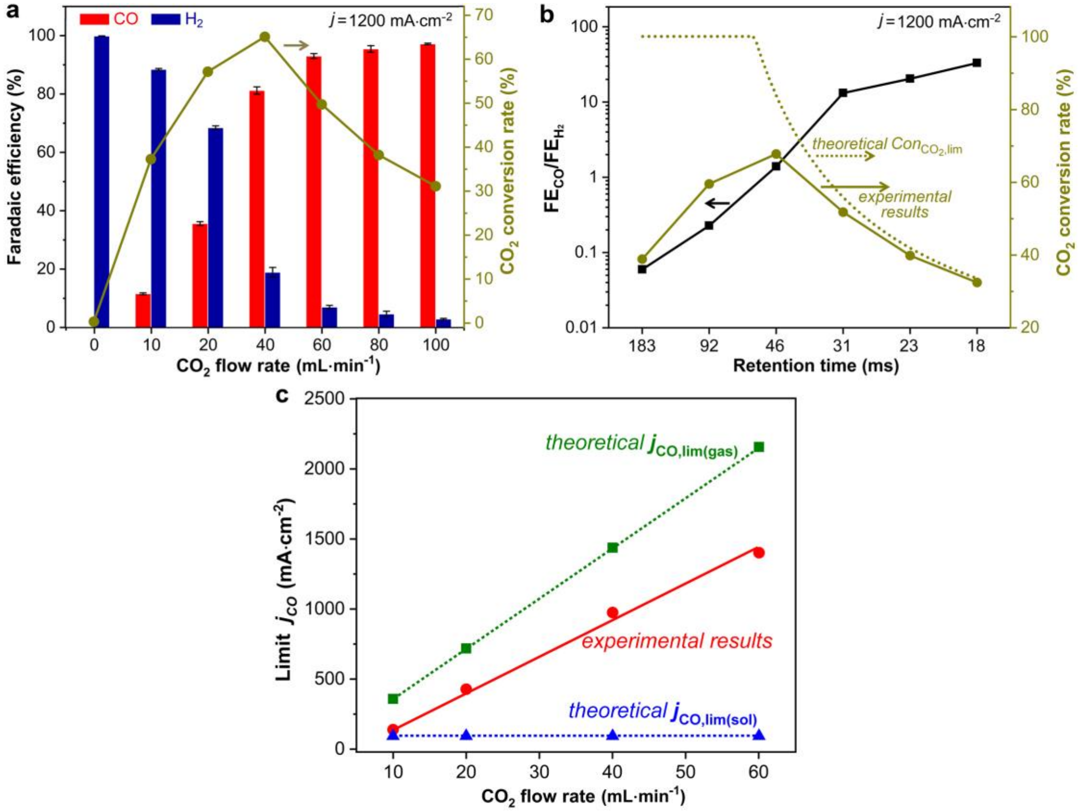

Supplementary Figure 13 | Electrocatalytic performance over the activated Ag HF electrode in CO_2-saturated 1.5 $KHCO_3$ solution under different CO_2 flow rates. Product faradaic efficiency and CO_2 conversion rate versus **a,** CO_2 flow rate and **b,** different retention time under the constant current density of 1200 mA·cm^{-2}. Error bars in **a** were obtained from the average of three individual tests. **c,** Comparison of experimental j_{CO} values with different theoretical limits ($\boldsymbol{j}_{\mathbf{CO,lim(gas)}}$ and $\boldsymbol{j}_{\mathbf{CO,lim(sol)}}$) at different CO_2 flow rates.

By varying the CO_2 flow rates, which further affect the retention time and mass transfer that are related to the main structural factors, the variations of electrocatalytic performance over the activated Ag HF electrode including CO_2 reduction and HER processes have been clearly presented. As shown in Supplementary Fig. 13a below, the CO_2 flow rate significantly influenced the product faradaic efficiency and CO_2 conversion rate over activated Ag HF electrode under the constant current density of 1.2 A·cm^{-2}. Only H_2 was detected when no CO_2 flowed through the porous electrode, indicating the dominant HER. With increasing CO_2 flow rates, the H_2 faradaic efficiencies monotonically decreased, and the CO faradaic efficiencies correspondingly increased resulting in a total faradaic efficiency of 100%. This implies that high local CO_2 concentration generated by the sufficient CO_2 flow suppressing HER while facilitating CO_2 reduction. However, compared to the CO faradaic efficiency, the CO_2 conversion rate exhibited different variations with respect to CO_2 flow rate. That is the CO_2 conversion rate increased rapidly at first with the gradually increasing CO_2 flow rates, and a maximum conversion of 68% was yielded at 40 mL·min^{-1} with a CO faradaic efficiency of 81%. Interestingly, the CO_2 conversion rate faded with further increasing CO_2 flow rates, even down to 32% at 100 mL·min^{-1}. These results imply that CO_2 reduction kinetics may also be affected by the electrode intrinsic structures besides the competitive HER.

Furthermore, variations on the faradaic efficiency ratio of FE_{CO}/FE_{H2} and the CO_2 conversion rate with respect to the retention time (obtained from **Equations** (**5**), (**6**) and (**7**) on basis of the electrode intrinsic structure characteristics) under the constant current density of 1.2 $A \cdot cm^{-2}$ can be clearly seen in Supplementary Fig. 13b. The FE_{CO}/FE_{H2} ratio increased when the retention time decreased. Regarding the theoretical limit of CO_2 conversion rate, i.e., $\boldsymbol{Con}_{\mathbf{CO_2,lim}}$ (referring to **Equation** (**11**)), it remained at 100% in the retention time range from 183 to 46 ms (corresponding to CO_2 flow rates from 10 to 40 $mL \cdot min^{-1}$), and then decreased rapidly with the further decreasing retention time. In fact, the experimental results of CO_2 conversion rates were quite low at long retention time situations and then close to the theoretical values at short retention time situations. In order to obtain both appropriate CO faradaic efficiency and CO_2 conversion rate, the CO_2 flow rate was fixed at 60 $mL \cdot min^{-1}$ during CO_2 electroreduction unless otherwise stated.

In addition, the theoretical limit and experimental values of CO partial current density under different CO_2 flow rates were further studied. Actually, there are two kinds of theoretical limits of CO partial current density: (1) all gas-phase CO_2 molecules input into the electrolysis cell are reduced to CO with a 100% conversion rate, i.e., $\boldsymbol{j}_{\mathbf{CO,lim(gas)}}$, which is calculated using above **Equation** (**8**); (2) all CO_2 molecules dissolved in the electrolyte solution are reduced to CO, i.e., $\boldsymbol{j}_{\mathbf{CO,lim(sol)}}$, which is calculated using above **Equation** (**9**). As shown in Supplementary Fig. 13c, the experimental $\boldsymbol{j}_{\mathbf{CO}}$ values over activated Ag HF were far larger than those of $\boldsymbol{j}_{\mathbf{CO,lim(sol)}}$. Although the experiment results were still lower than those of $\boldsymbol{j}_{\mathbf{CO,lim(gas)}}$ as an extremely ideal case, the activated Ag HF delivered a maximum $\boldsymbol{j}_{\mathbf{CO}}$ of 1.40 $A \cdot cm^{-2}$ at 60 $mL \cdot min^{-1}$, superior to the previous reports (Supplementary Table 1). In contrast, all $\boldsymbol{j}_{\mathbf{CO}}$ values over activated Ag foil (Fig. 4b) were lower than those of the theoretical $\boldsymbol{j}_{\mathbf{CO,lim(sol)}}$. Moreover, on basis of **Equation** (**8**), the mass transfer coefficients were 0.4, 0.6, 0.7 and 0.7 over activated Ag HF, corresponding to the CO_2 flow rates of 10, 20, 40 and 60 $mL \cdot min^{-1}$, respectively, which were much larger than those over activated Ag foil.

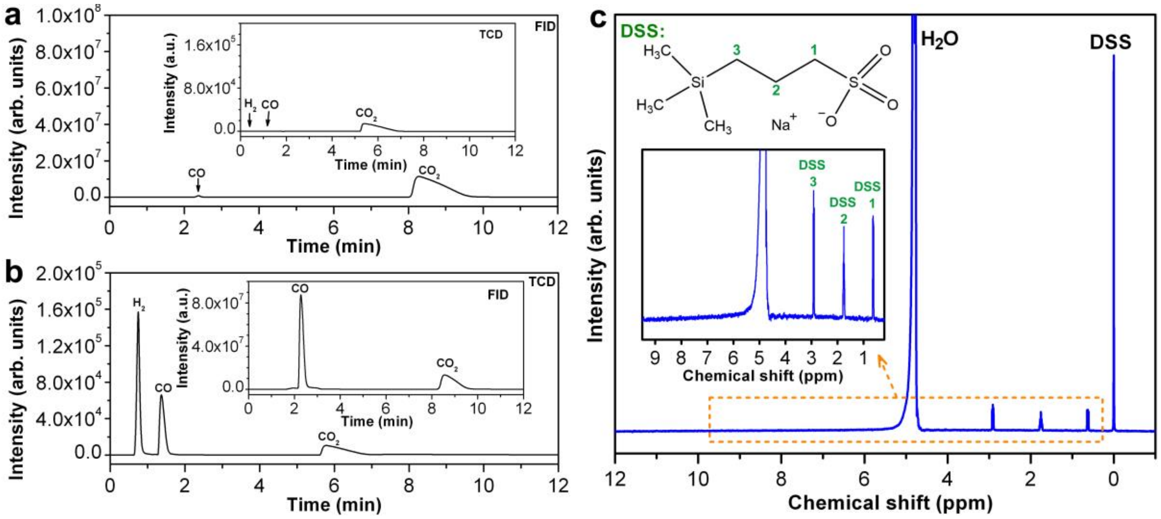

Supplementary Figure 14 | Product measurement curves. Typical GC curves from the FID and TCD over activated Ag HF at **a,** -0.35 V and **b,** -0.83 V. **c,** ^{1}H-NMR spectra of the postreaction catholyte solution after 1 h of CO_2 electroreduction over activated Ag HF at -0.83 V. CO_2-saturated 1.5 M $KHCO_3$ as the electrolyte solution, and the CO_2 flow rate of 60 mL·min^{-1}.

Typical GC curves from the FID and TCD over activated Ag HF are shown in Supplementary Fig. 14. When the CO concentration in the exhaust was lower than 10%, the FID was used for CO quantification (Supplementary Fig. 14a). When the CO concentration in the exhaust was higher than 10%, the TCD was used as the main detector of CO, and the FID was used as the auxiliary detector (Supplementary Fig. 14b). The TCD was always used for H_2 quantification. Moreover, H_2 and CO were confirmed to be the only gas-phase products. The ^{1}H-NMR spectrum of the postreaction catholyte solution after 1 h of CO_2 electroreduction over activated Ag HF at -0.83 V further verified that no liquid-phase product could be detected (Supplementary Fig. 14c).

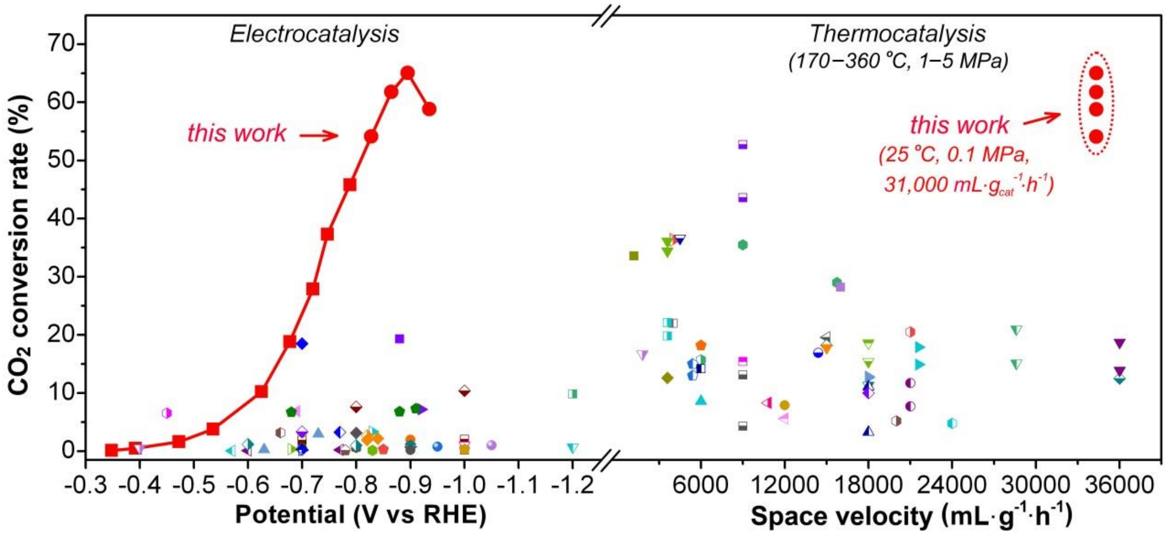

Supplementary Figure 15 | CO_2 conversion rates at different potentials. CO_2 conversion rates with a space velocity of 31,000 mL·g_{cat}^{-1}·h^{-1} at different potentials, and their overall comparison with other electrocatalytic and thermocatalytic CO_2 conversions. CO_2-saturated 1.5 M $KHCO_3$ as the electrolyte solution, and the CO_2 flow rate of 60 mL·min^{-1}.

As shown in Supplementary Fig. 15, the CO_2 conversion rates of activated Ag HF were comparable to those over prominent catalysts reported in electrocatalysis in the potential range of -0.35 to -0.70 V. With negative-shifting potentials, the CO_2 conversion rates over the activated Ag HF electrode further increased rapidly and reached 28%, 37%, 54% and 65% at -0.72 V, -0.75 V, -0.83 V and -0.89 V, respectively (Supplementary Fig. 15), far outperforming the previously reported electrocatalysts (Supplementary Table 1).

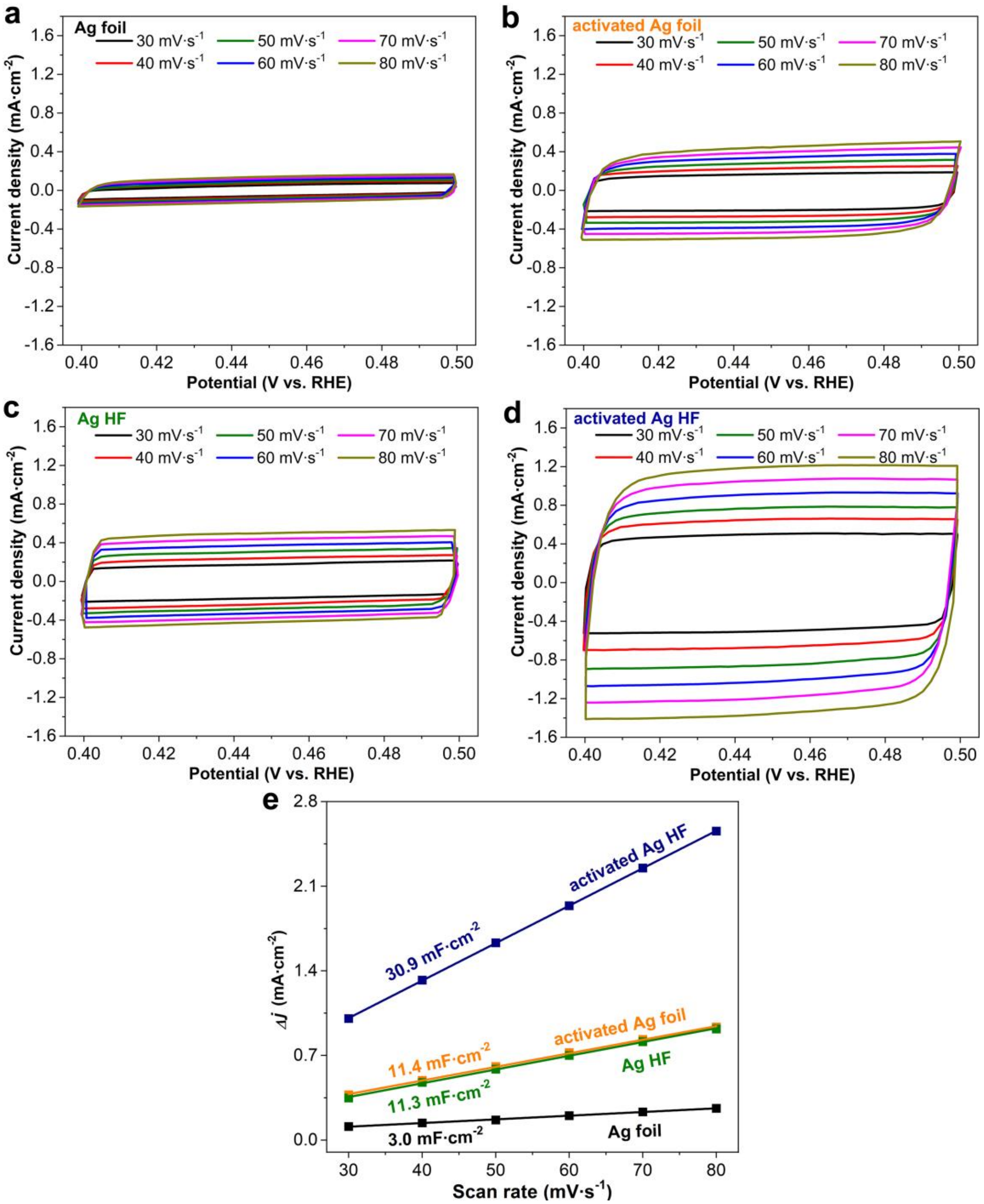

Supplementary Figure 16 | ECSA measurement results. Cyclic voltammetry curves of **a,** Ag foil, **b,** activated Ag foil, **c,** Ag HF, and **d,** activated Ag HF in CO_2-saturated 1.5 M $KHCO_3$. **e,** Plot of Δj (the difference of cathodic and anodic current densities, j_c–j_a) against the scan rates from cyclic voltammetry curves. The plots in Supplementary Fig. 16e, same as Fig. 4a in the main text. All the current densities in the main text and Supplementary Information were based on the electrode geometric area.

The ECSAs of Ag foil, activated Ag foil, Ag HF and activated Ag HF were determined by measuring their double-layer capacitance (C_{dl}) values via their cyclic voltammetry curves, as

shown in Supplementary Figs. 16a–d. The C_{dl}, which was proportional to the ECSA, was obtained by linearly fitting the absolute value of the slope of Δj (the difference of cathodic and anodic current densities of the cyclic voltammetry curves) against the scan rates. Activated Ag HF possessed the largest ECSA with a C_{dl} value of 30.9 mF·cm^{-2}, and this value was 2.7, 2.7 and 10.3 times those of activated Ag foil (11.4 mF·cm^{-2}), Ag HF (11.3 mF·cm^{-2}) and Ag foil (3.0 mF·cm^{-2}), respectively (Supplementary Fig. 16e).

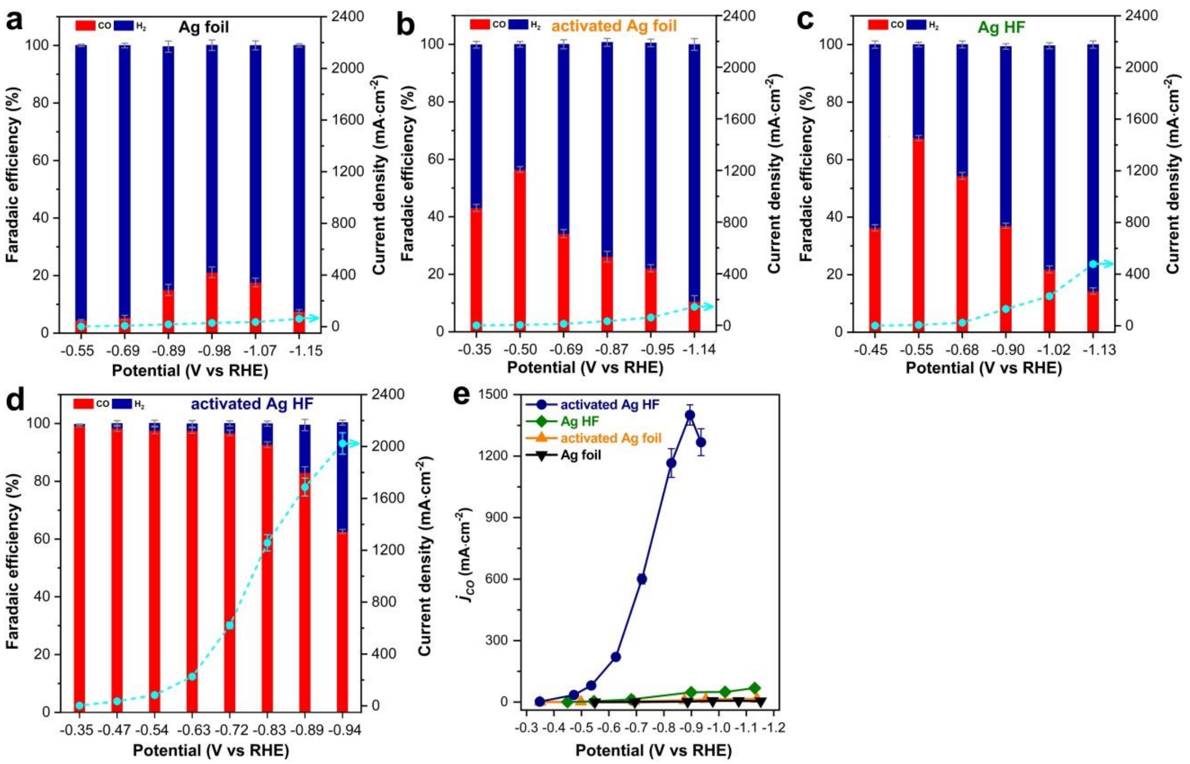

Supplementary Figure 17 | Electrocatalytic performance of activated Ag HF and other counterparts. CO and H_2 faradaic efficiencies, and total current densities over the electrodes **a,** Ag foil, **b,** activated Ag foil, **c,** Ag HF, and **d,** activated Ag HF in the potential range of -0.35 to -1.15 V in CO_2-saturated 1.5 $KHCO_3$. **e,** Comparison of the CO partial current densities over these electrodes. The plots in Supplementary Fig. 17e, same as Fig. 4b in the main text. Error bars in **a-e** were obtained from the average of six individual tests.

Ag foil showed very low CO_2 electroreduction activity, and the CO faradaic efficiencies at all potentials were less than 22% (Supplementary Fig. 17a). After electrochemical redox treatments, the CO faradaic efficiencies of activated Ag foil improved to some degree (Supplementary Fig. 17b). At -0.50 V, the CO faradaic efficiency of activated Ag foil reached a maximum of 57%. While activated Ag foil also delivered CO faradaic efficiencies that were far less than 50% at other potentials (Supplementary Fig. 17b). The results implied that the hydrogen evolution reaction was still dominant with the activated Ag foil. As shown in Supplementary Fig. 17c, Ag HF showed slightly better CO_2 electroreduction activity than activated Ag foil, i.e., higher faradaic efficiencies and total current densities at similar potentials. With respect to activated Ag HF, all of the CO faradaic efficiencies and total current densities increased greatly (Supplementary Fig. 17d). The comparison of the CO partial current densities indicated the obvious superiority over the activated Ag HF electrode compared with the other electrodes (Supplementary Fig. 17e).

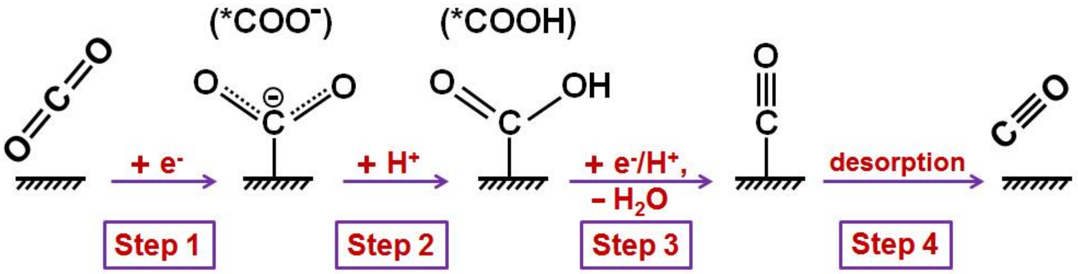

Supplementary Figure 18 | CO_2 electroreduction path. Reaction steps for the electroreduction of CO_2 to CO on silver catalysts.

Supplementary Figure 18 shows a possible mechanism for CO_2 electroreduction on silver catalysts, in which the reaction paths proposed were consistent with previous reports[10,11]. The initial step (**Step 1**) in the overall two-electron reduction of CO_2 to CO on the silver surface was a one-electron transfer step forming adsorbed $*COO^-$. Then, **Step 2** was a chemical step involving the protonation of $*COO^-$ to form a *COOH intermediate. Subsequently, **Step 3** was an electrochemical path coupled to a chemical reaction involving the proton-electron transfer and instantaneous dehydration to form an adsorbed *CO intermediate. Finally, **Step 4** was the desorption of *CO from the silver surface to obtain the CO product. The Tafel slope of activated Ag foil was 113 $mV·dec^{-1}$, close to that (108 $mV·dec^{-1}$) of activated Ag HF with the non-CO_2-disperser mode (Fig. 5c), implying that **Step 1** with the theoretical value of 118 $mV·dec^{-1}$ [12] was the rate-determining step for both electrodes. In contrast, activated Ag HF with the CO_2-disperser mode showed a Tafel slope as low as 63 $mV·dec^{-1}$, suggesting **Step 1** was not the rate-determining step. In principle, each one of **Step 2**, **Step 3** and **Step 4** was probably to be the rate-determining step of activated Ag HF with the CO_2-disperser mode. According to the previous report[12,13], if **Step 3** was the rate-determining step, the Tafel slope will be generally less than 40 $mV·dec^{-1}$. In addition, if **Step 4** was the rate-determining step, the Tafel slope will be ∞ (infinity)[13,14]. Thus, the Tafel slope value (63 $mV·dec^{-1}$) of activated Ag HF with the CO_2-disperser mode ruled out the situations of **Step 3** and **Step 4** as the possible rate-determining steps. Consequently, **Step 2** with the theoretical value of 59 $mV·dec^{-1}$ was the rate-determining step[13,14] for activated Ag HF with the CO_2-disperser mode, in agreement with many reports[15-17]. The result meant that the CO_2-disperser mode of activated Ag HF played a crucial role in CO_2 electroreduction, which might induce the synergistic effects to alter the route of CO_2 reduction.

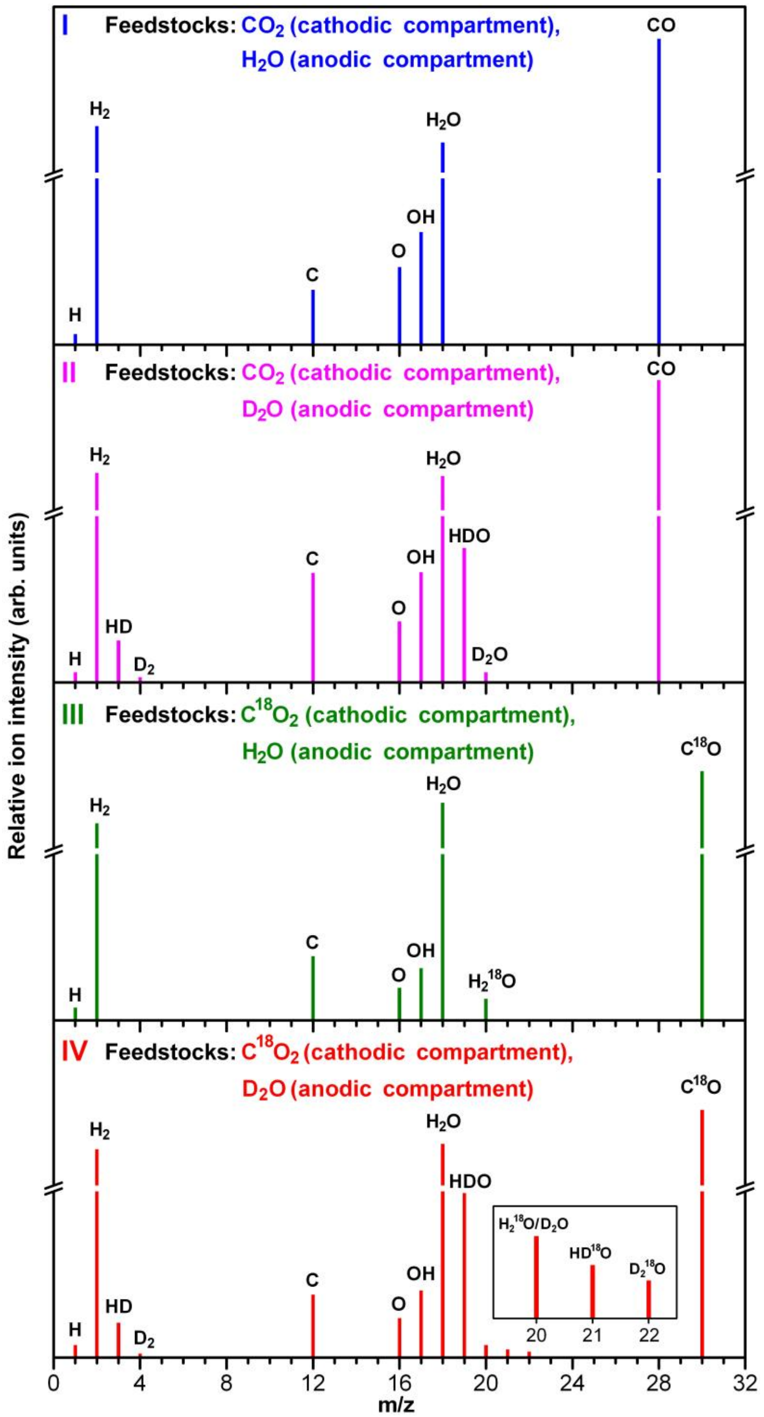

Supplementary Figure 19 | Mass spectrometric detection of the cathodic products over activated Ag HF. Using different feedstock supplies: (**I**) CO_2 (cathodic compartment), H_2O (anodic compartment); (**II**) CO_2 (cathodic compartment), D_2O (anodic compartment); (**III**) $C^{18}O_2$ (cathodic compartment), H_2O (anodic compartment); (**IV**) $C^{18}O_2$ (cathodic compartment), D_2O (anodic compartment).

Isotopic trace experiments were conducted to study the mass migrations involved in CO_2 electroreduction over activated Ag HF. For comparison, the feedstocks were supplied into the

cathodic and anodic compartments of the electrolysis cell according to the below four situations, respectively, which were subjected to the potentiostatic electrolysis under the same reaction conditions (see the Materials and Methods section for details).

In situation (**I**) CO_2 (cathodic compartment), H_2O (anodic compartment) were used as the feedstocks, and the mass spectrum in Supplementary Fig. 19-**I** shows the signals of CO (*m*/*z*=28), H_2O (*m*/*z*=18, 17) and H_2 (*m*/*z*=2, 1), implying the occurrence of the reactions below:

cathodic reactions:

$$CO_2 + 2H^+ + 2e^- \rightarrow CO\uparrow + H_2O$$
$$2H^+ + 2e^- \rightarrow H_2\uparrow$$

anodic reaction:

$$H_2O \rightarrow 1/2O_2\uparrow + 2H^+ + 2e^-$$

In situation (**II**) CO_2 (cathodic compartment), D_2O (anodic compartment) were used as the feedstocks, and the mass spectrum in Supplementary Fig. 19-**II** shows the signals of CO (*m*/*z*=28), H_2O (*m*/*z*=18, 17), HDO (*m*/*z*=19), D_2O (*m*/*z*=20), H_2 (*m*/*z*=2, 1), HD (*m*/*z*=3) and D_2 (*m*/*z*=4), implying the occurrence of the reactions below:

cathodic reactions:

$$CO_2 + 2H^+ + 2e^- \rightarrow CO\uparrow + H_2O$$
$$CO_2 + H^+ + D^+ + 2e^- \rightarrow CO\uparrow + HDO$$
$$CO_2 + 2D^+ + 2e^- \rightarrow CO\uparrow + D_2O$$
$$2H^+ + 2e^- \rightarrow H_2\uparrow$$
$$H^+ + D^+ + 2e^- \rightarrow HD\uparrow$$
$$2D^+ + 2e^- \rightarrow D_2\uparrow$$

anodic reaction:

$$D_2O \rightarrow 1/2O_2\uparrow + 2D^+ + 2e^-$$

In situation (**III**) $C^{18}O_2$ (cathodic compartment), H_2O (anodic compartment) were used as the feedstock, and the mass spectrum in Supplementary Fig. 19-**III** shows the signals of $C^{18}O$ (*m*/*z*=30), H_2O (*m*/*z*=18, 17), $H_2{}^{18}O$ (*m*/*z*=20) and H_2 (*m*/*z*=2, 1), implying the occurrence of the reactions below:

cathodic reactions:

$$C^{18}O_2 + 2H^+ + 2e^- \rightarrow C^{18}O\uparrow + H_2^{18}O$$
$$2H^+ + 2e^- \rightarrow H_2\uparrow$$

anodic reaction:

$$H_2O \rightarrow 1/2O_2\uparrow + 2H^+ + 2e^-$$

In situation (**IV**) $C^{18}O_2$ (cathodic compartment), D_2O (anodic compartment) were used as the feedstocks, and the mass spectrum in Supplementary Fig. 19-**IV** shows the signals of $C^{18}O$ (*m*/*z*=30), H_2O (*m*/*z*=18, 17), HDO (*m*/*z*=19), D_2O (*m*/*z*=20), $H_2{}^{18}O$ (*m*/*z*=20), $HD^{18}O$ (*m*/*z*=21), $D_2{}^{18}O$, (*m*/*z*=22), H_2 (*m*/*z*=2, 1), HD (*m*/*z*=3) and D_2(*m*/*z*=4), implying the occurrence of the reactions below:

cathodic reactions:

$$C^{18}O_2 + 2H^+ + 2e^- \rightarrow C^{18}O\uparrow + H_2^{18}O$$
$$C^{18}O_2 + H^+ + D^+ + 2e^- \rightarrow C^{18}O\uparrow + HD^{18}O$$
$$C^{18}O_2 + 2D^+ + 2e^- \rightarrow C^{18}O\uparrow + D_2^{18}O$$
$$2H^+ + 2e^- \rightarrow H_2\uparrow$$
$$H^+ + D^+ + 2e^- \rightarrow HD\uparrow$$
$$2D^+ + 2e^- \rightarrow D_2\uparrow$$

anodic reaction:

$$D_2O \rightarrow 1/2O_2\uparrow + 2D^+ + 2e^-$$

These isotopic trace results confirmed that the product CO originated from the reduction of CO_2 and the anodic reaction maintained the proton and charge balances of the overall CO_2 electroreduction reaction.

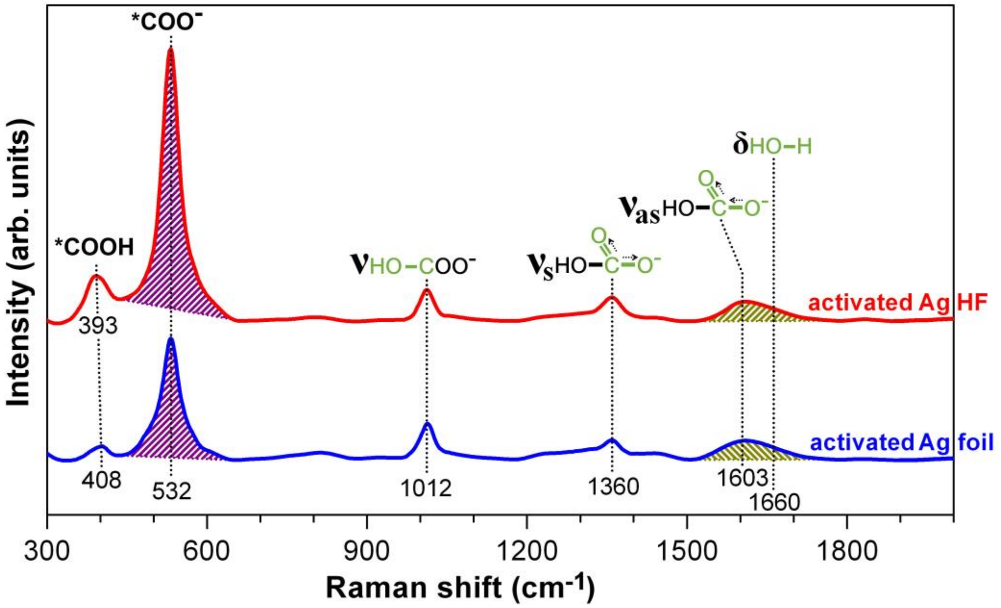

Supplementary Figure 20 | Typical Raman spectra in the range of 300 to 2000 cm^{-1}. Operando Raman spectra at the stable states over activated Ag HF and activated Ag foil during CO_2 electroreduction. v: stretching, v_s: symmetric stretching, v_{as}: antisymmetric stretching, and δ: deformation.

For both activated Ag HF and activated Ag foil, typical operando Raman spectra in a wide range of 300–2000 cm^{-1} showed two kinds of Raman peaks above and below 1000 cm^{-1}, respectively, as shown in Supplementary Fig. 20. The Raman peaks above 1000 cm^{-1} included four peaks centered approximately at 1012, 1360, 1603 and 1660 cm^{-1}, which were assigned to bicarbonate ions (HCO_3^-) adsorbed on the electrode surface as $\boldsymbol{v}_{\text{HO–COO}}$-, $\boldsymbol{v_s}_{\text{HOCOO}}$-, $\boldsymbol{v_{as}}_{\text{HOCOO}}$- and $\boldsymbol{\delta}_{\text{HO–H}}$ (in H_2O) modes, respectively, according to previous reports[6,7]. The other Raman peaks below 1000 cm^{-1} showed only two Raman bands at 532 and 390–410 cm^{-1}, which could be assigned to the adsorbed intermediate vibrations, i.e., $\boldsymbol{v}_{\text{*COO}}$- and $\boldsymbol{v}_{\text{Ag–*COOH}}$, in consistence with previous reports[18,19]. The lower $\boldsymbol{v}_{\text{Ag–*COOH}}$ frequency (393 cm^{-1}) of activated Ag HF compared with that (408 cm^{-1}) of activated Ag foil suggested a weaker bonding strength between *COOH and the activated Ag HF surface. Furthermore, the *COO$^-$ and *COOH intermediates appeared in chronological order in the time-resolved operando continuous Raman spectra (Fig. 6b and Supplementary Fig. 21c), implying the step-by-step reduction of CO_2, i.e., the initial step to form *COO$^-$ and the second step to form *COOH, in agreement with the proposed mechanism (Supplementary Fig. 18). Considering its higher sensitivity and intensity, the *COO$^-$ intermediate was given more attention in the following.

For comparison of the relative intensity of *COO$^-$ in these two electrodes, namely, activated Ag HF and activated Ag foil, we estimated the relative ratio of the integral peak areas of adsorbed *COO$^-$ and aqueous HCO_3^- (i.e., $\boldsymbol{v_{as}}_{\text{HOCOO}}^-$ + $\boldsymbol{\delta}_{\text{HO–H}}$), which are marked with shadows in Supplementary Fig. 20. The *COO$^-$/($\boldsymbol{v_{as}}_{\text{HOCOO}}$- + $\boldsymbol{\delta}_{\text{HO–H}}$) ratio was 6.7 for activated Ag HF after power-on for 2720 ms, and this ratio value did not change in the following stable state. In contrast, this ratio in the stable state was 3.3 for activated Ag foil, which was only half of that of activated Ag HF. This result implied that more *COO$^-$ intermediates were formed and adsorbed on the surface of activated Ag HF.

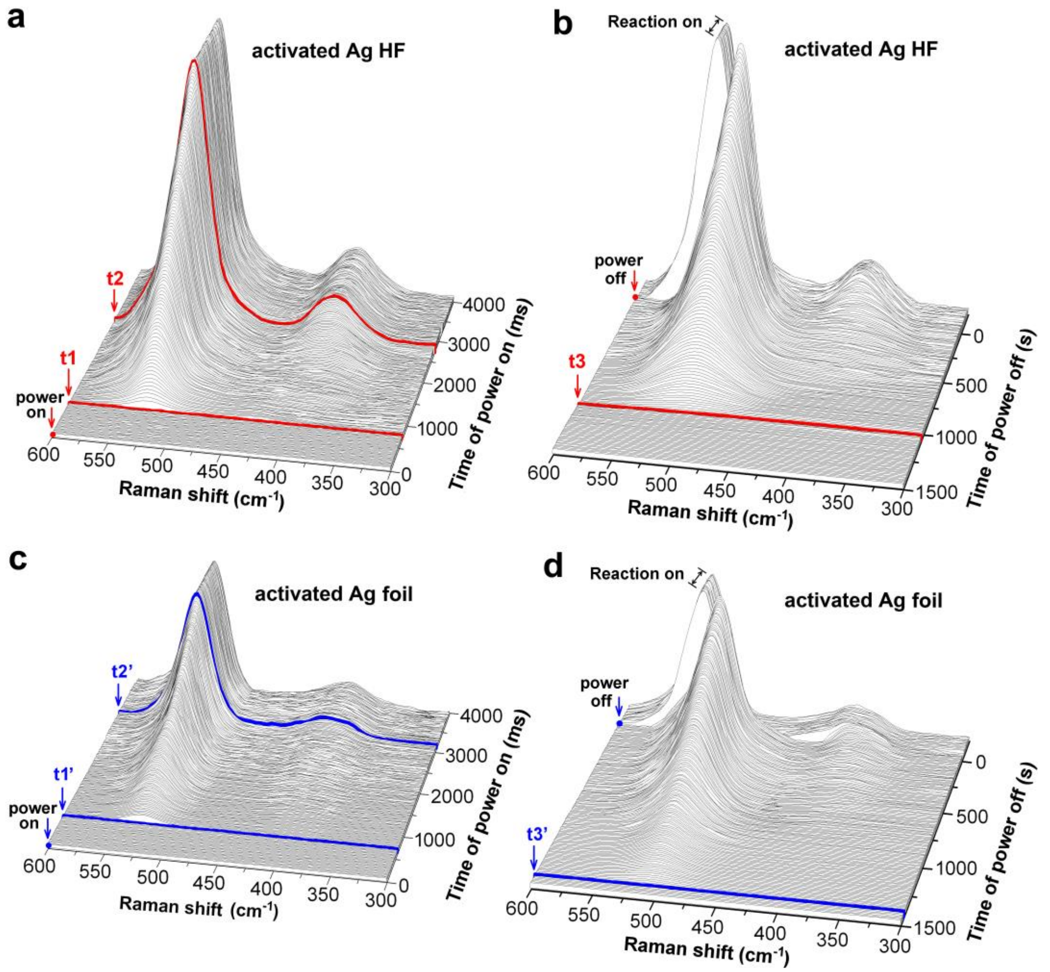

Supplementary Figure 21 | Time-resolved operando Raman spectra in the range of 300 to 600 cm^{-1}. a, b, activated Ag HF and **c, d,** activated Ag foil during **a, c,** power-on and **b, d,** power-off stages, respectively. The operando Raman spectra of activated Ag HF in Supplementary Figs. 21a and b, same as Fig. 6b and c in the main text.

The formation and evolution of key intermediates over activated Ag HF and activated Ag foil were monitored by time-resolved operando Raman spectroscopy during the power-on and power-off stages, respectively (Supplementary Fig. 21). After power-on for 720 ms (**t1**), a new Raman peak appeared at 532 cm^{-1} over activated Ag HF, corresponding to the adsorbed $*COO^-$ intermediate (Supplementary Fig. 20). Then, the peak intensity increased quickly and reached the maximum at 2720 ms (**t2**) (Supplementary Fig. 21a and Fig. 6d). Regarding activated Ag foil, the $*COO^-$ Raman peak appeared at 660 ms (**t1'**), and the peak intensity reached a maximum at 3080 ms (**t2'**) (Supplementary Fig. 21c and Fig. 6d). Notably, the normalized $*COO^-$ peak intensity of activated Ag HF was almost double that of activated Ag foil in the stable state. These results indicated that more $*COO^-$ intermediates were formed and adsorbed over activated Ag HF in a shorter time, implying the superior capability of CO_2 activation, which probably profited from the reduced CO_2 diffusion distance in the CO_2-disperser mode.

Subsequently, we investigated the variation of adsorbed $*COO^-$ over activated Ag HF and activated Ag foil during the power-off stages (Supplementary Figs. 21b, d. As soon as the power was turned off, the 532 cm^{-1} $\boldsymbol{\nu}_{*COO^-}$ peak quickly redshifted for both electrodes due to the Stark

effect[20-22], indicating the distinct impact of electric field on the adsorption of intermediates (Supplementary Fig. 22). Then, the intensity of the $*COO^-$ Raman peak decreased gradually. The $*COO^-$ peak vanished over activated Ag HF after power-off for 1050 s (**t3**) (Supplementary Fig. 21b and Fig. 6d), whereas over activated Ag foil after power-off for 1400 s (**t3'**) (Supplementary Fig. 21d and Fig. 6d), indicating a faster dissipation of adsorbed $*COO^-$ over activated Ag HF. This result implied that the one-way CO_2 flow manner of activated Ag HF facilitated the desorption of adsorbed intermediates or species on its surface (vide infra).

The above time-resolved operando Raman results suggested that the oriented mass transfers induced by the CO_2-disperser mode of activated Ag HF could not only favor the diffusion of CO_2 to active sites but also facilitate the desorption of adsorbed species from the electrode surface, thereby resulting in the improved overall kinetics of CO_2 reduction. Consequently, activated Ag HF also demonstrated the promotion in mass transfers of CO_2 electroreduction in addition to enhanced three-phase interface reactions.

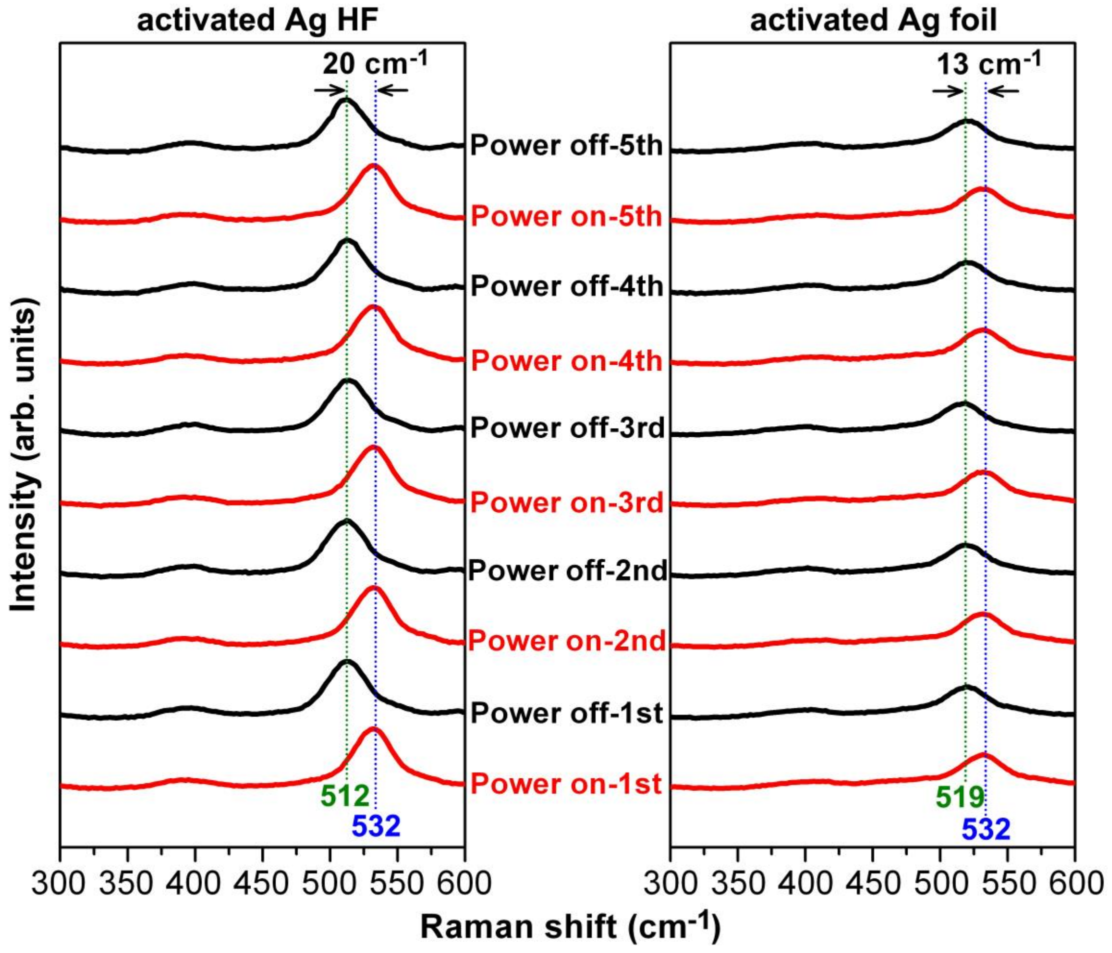

Supplementary Figure 22 | Electric field impact on Raman spectra. Raman spectra (300−600 cm^{-1}) of activated Ag HF and activated Ag foil during frequent switching between power-on and power-off.

To study the Stark effect of electric field on the adsorbed intermediates, Raman spectra over activated Ag HF and activated Ag foil were recorded during frequent switching between power-on and power-off, as shown in Supplementary Fig. 22. In power-on situations from power on-1st to power on-5th, all $*COO^-$ Raman peaks were located at 532 cm^{-1} for both activated Ag HF and activated Ag foil, while the $*COO^-$ peak quickly and consistently redshifted for both electrodes when switched to power-off, indicating the reproducible occurrences of the Stark effect[20-22]. This result indicated that electric field could play a crucial impact on the adsorption of surface intermediates. In detail, $\boldsymbol{\nu}_{*COO}$- redshifted to 512 cm^{-1} over activated Ag HF, whereas to 519 cm^{-1} over activated Ag foil. Note that there was a 7 cm^{-1} shift in $*COO^-$ vibration peak between activated Ag HF and activated Ag foil during all power-off situations. The lower frequency suggested the weaker interaction of $*COO^-$ with the surface of activated Ag HF, which was another sign of its easier desorption of adsorbed intermediates or species when compared to activated Ag foil. These results implied that activated Ag HF was intrinsically favorable for the desorption of adsorbed surface species.

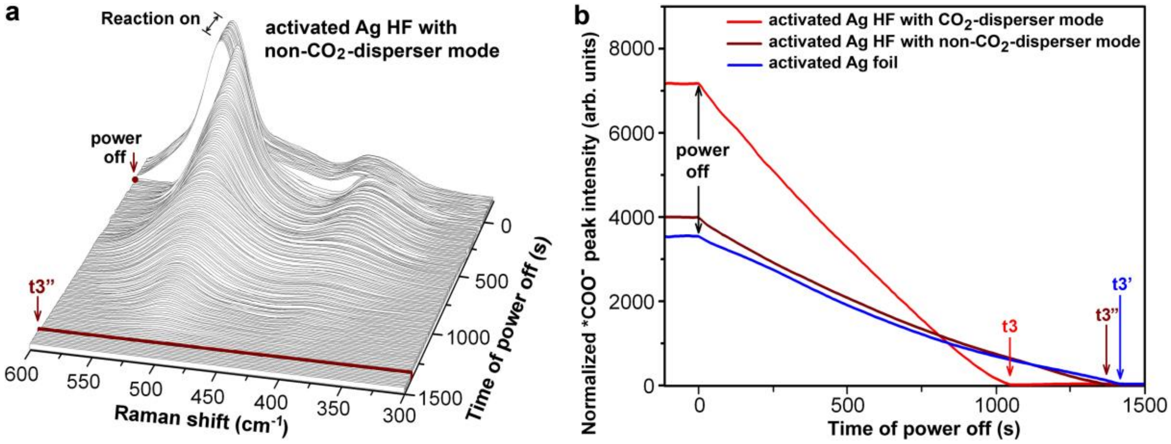

Supplementary Figure 23 | Time-resolved operando Raman results. a, Time-resolved operando Raman spectra showing the dissipation of *COO^- over activated Ag HF with non-CO_2-disperser mode. **b,** Comparison of the normalized *COO^- peak intensities of activated Ag foil and activated Ag HF with CO_2-disperser/non-CO_2-disperser modes during the power-off stage.

The desorption of *COO^- over activated Ag HF with non-CO_2-disperser mode was also monitored by time-resolved Raman spectra. As shown in Supplementary Fig. 23, the *COO^- peak vanished over activated Ag HF with non-CO_2-disperser mode after power-off for 1380 s (**t3’’**), which was close to the dissipation time of 1400 s (**t3’**) over activated Ag foil (Supplementary Fig. 21d). This result also demonstrate that the CO_2-disperser mode played a key role for the desorption of adsorbed species.

Supplementary Table 1 | CO_2 reduction performances over prominent catalysts reported recently.

Electrocatalysis								
**Catalysts**	**Electrolyte**	**Potential (V *vs.* RHE)**	**j_{total} ($mA{\cdot}cm^{-2}$)**	**FE (%)**	**CO_2 flow rate ($mL{\cdot}min^{-1}$)**	**CO_2 conv. (%)**	**Stability**	**Notes**
activated Ag HF	1.5 M $KHCO_3$	-0.83	1262.4	92.7 (CO)	60	54.12	170 h (-0.83 V, 1.5 M $KHCO_3$)	This work
	1.5 M $KHCO_3$	-0.86	1467.6	90.6 (CO)	60	61.76		
	1.5 M $KHCO_3$	-0.89	1687.2	83.1 (CO)	60	65.07		
	1.5 M $KHCO_3$	-0.94	2023.2	62.6 (CO)	60	58.84		
F-Cu	0.75 M KOH	-0.89	1600.0	80.0 (C_{2+})	20	19.30	40 h (400 $mA{\cdot}cm^{-2}$, 1 M KOH)	Sr[23]
$Sn_{2.7}Cu$	1 M KOH	-0.70	406.7	98.0 (C_1)	30	18.51	40 h (-0.55 V, 1 M KOH)	Sr[24]
InP CQDs	3 M KOH	-2.60	1000.0	93.1 ($HCOO^-$)	50	12.97	4 h (400 $mA{\cdot}cm^{-2}$, 1 M KOH)	Sr[25]
InP CQDs	3 M KOH	-2.30	800.0	91.1 ($HCOO^-$)	50	10.15		Sr[25]
Ni–N_4/C–NH_2	1 M KOH	-1.00	526.6	85.0 (CO)	30	10.39	6 h (-1.00 V)	Sr[26]
Ni–N_4/C–NH_2	1 M KOH	-0.80	368.3	89.0 (CO)	30	7.61	/	Sr[26]
CdS NNs	5 M KOH	-1.20	222.0	95.5 (CO)	60	9.84	4 h (-1.20 V)	Sr[27]
F-γ-In_2Se_3/CP	[Bmim]PF_6/ MeCN	-2.00 V *vs.* SCE	57.3	96.5 (CO)	5	7.70	25 h (-2.00 V *vs.* SCE)	Sr[28]
Cu/Ionomer	7 M KOH	-0.91	1550.0	78.2 (C_{2+})	50	7.32	60 h (-3.90 V cell voltage, 0.1 M $KHCO_3$)	Sr[29]
Cu/Ionomer	7 M KOH	-0.88	1370.0	79.5 (C_{2+})	50	6.81		Sr[29]
Cu/Ionomer	7 M KOH	-0.68	1170.0	87.5 (C_{2+})	50	6.72		Sr[29]
CoPc2	1 M KOH	-0.92	178.4	~92.5 (CO)	16	7.18	10 h (-0.65 V)	Sr[30]
NiPc-OMe-MDE	1 M $KHCO_3$	-0.69	400.0	99.1 (CO)	20	6.90	40 h (-150 $mA{\cdot}cm^{-2}$)	Sr[31]
nBuLi-Bi	1 M $KHCO_3$	-1.05	500.0	96.0 ($HCOO^-$)	50	6.69	100 h (30 $mA{\cdot}cm^{-2}$)	Sr[32]
Fe^{3+}–N–C	0.5 M $KHCO_3$	-0.45	94.0	>90.0 (CO)	40	6.55	~25 h (-0.41 V)	Sr[14]
Au/C-P-0.5	1 M KOH	-0.48	108.4	70.9 (CO)	20	6.42	10 h (100 $mA{\cdot}cm^{-2}$, 1 M KOH)	Sr[33]
NPCA900	[Bmim]PF_6/ MeCN	-2.40 V *vs.* Ag/AgCl	144.9	99.1 (CO)	20	6.00	24 h (-2.40 V *vs.* Ag/AgCl)	Sr[34]
Bi/CeO_x	0.2 M Na_2SO_4	-1.30	148.9	92.0 ($HCOO^-$)	20	4.77	34 h (-1.20 V)	Sr[35]
CoPc	0.5 M $KHCO_3$	2.25 V cell voltage	150.0	>95.0 (CO)	100	3.90	100 h (-50 $mA{\cdot}cm^{-2}$)	Sr[36]
Cu-12	1 M $KHCO_3$	-0.83	322.0	72.0 (C_2H_4)	80	3.36	190 h (120 $mA{\cdot}cm^{-2}$, 0.1 M $KHCO_3$)	Sr[37]
CoTMAPc@CNT	1 M KOH	-0.70	250.0	95.6 (CO)	50	3.33	15 h (-0.40 V)	Sr[38]
Ni@NiNCM	1 M $KHCO_3$	-0.77	100.0	93.7 (CO)	20	3.26	10 h (-0.90 V)	Sr[39]
NCMSH	4 mol% Emim-BF_4	-0.70	34.3	92.7 (CO)	7	3.16	24 h (-0.70 V)	Sr[40]

Catalysts	Electrolyte	Potential (V *vs.* RHE)	j_{total} ($mA \cdot cm^{-2}$)	FE (%)	CO_2 flow rate ($mL \cdot min^{-1}$)	CO_2 conv. (%)	Stability	Notes
Ir_{AC}-1.7	0.5 M $KHCO_3$	-0.66	18.6	97.6 (CO)	2	3.16	24 h (-0.66 V)	Sr[41]
P-Cd|S	0.25 M $KHCO_3$	-0.80	102.0	88.0 (CO)	20	3.13	2 h (-0.80 V)	Sr[42]
Ni^{I}-NCNT@Ni_9Cu	0.5 M $KHCO_3$	-0.73	32.9	97.0 (CO)	7.5	2.96	20 h (-0.73 V)	Sr[43]
FeTPP[Cl]/Cu	1 M $KHCO_3$	-0.82	302.4	80.0 (C_2H_4, C_2H_5OH)	50	2.68	12 h (3.70 V cell voltage)	Sr[44]
Ag-NOLI	1 M $KHCO_3$	-0.84	500.0	84.0 (CO)	40	2.19	6 h (200 $mA \cdot cm^{-2}$)	Sr[45]
Ag-NOLI	1 M $KHCO_3$	-0.82	400.0	92.6 (CO)	40	1.93		Sr[45]
C/Ag/PTFE	1 M $KHCO_3$	-1.00	160.0	>90.0 (CO)	50	2.01	100 h (-1.00 V)	Sr[46]
C/Ag/PTFE	1 M KOH	-0.70	150.0	> 90.0 (CO)	50	1.88	100 h (-0.70 V)	Sr[46]
Co@Pc/C	0.5 M $KHCO_3$	-0.90	33.3	84.0 (CO)	10	1.95	20 h (-0.90 V)	Sr[47]
FNC-SnOF	1 M KOH	-0.60	186.0	~90.0 (CO)	50	1.17	27.8 h (-0.85 V)	Sr[48]
FNC-SnOF	1 M $KHCO_3$	-0.80	150	93.8 (CO)	50	0.98		Sr[48]
DNG-SAFe	0.1 M $KHCO_3$	-0.95	36.7	90.0 (CO)	30	0.77	20 h (-0.75 V)	Sr[49]
fcc-2H-fcc Au NRs	0.5 M $KHCO_3$	-0.90	25.0	~85.0 (CO)	20	0.74	24 h (-0.60 V)	Sr[50]
Sn/Cu-PVDF	0.1 M $KHCO_3$	-1.20	130.0	80.0 (CO)	20	0.71	135 h (-1.00 V)	Sr[51]
Pd_5@Au_{95}	0.1 M $KHCO_3$	-0.80	8.8	59.0 (CO)	20	0.64	24 h (-0.50 V)	Sr[52]
Ni_1-N-C	0.5 M $KHCO_3$	-0.80	27.9	96.8 (CO)	10	0.63	10 h (-0.80 V)	Sr[53]
Sn-OD-Cu	0.5 M $KHCO_3$	-0.70	8.5	94.6 (CO)	20	0.56	24 h (-0.80 V, 0.1 M $KHCO_3$)	Sr[54]
Ni/Fe-N-C	0.5 M $KHCO_3$	-1.00	23.7	80.0 (CO)	10	0.53	30 h (-0.70 V)	Sr[55]
Au nanoneedles	0.5 M $KHCO_3$	-0.40	38.0	95.0 (CO)	15	0.50	1 h (-0.40 V)	Sr[56]
Fe-CNPs	1 M $KHCO_3$	-0.68	10.0	97.0 (CO)	20	0.34	8 h (-0.68 V)	Sr[57]
[$Au_{11}(PPh_3)_7(NHC^{Me})Cl_2$]Cl	0.1 M $KHCO_3$	-1.00	8.6	~80.0 (CO)	15	0.32	/	Sr[58]
Pd_2-DAC	0.5 M $KHCO_3$	-0.85	6.9	98.2 (CO)	15	0.31	12 h (-0.80 V)	Sr[59]
SANi-GO	0.5 M $KHCO_3$	-0.63	8.6	96.6 (CO)	20	0.29	50 h (-0.63 V)	Sr[60]
Co(II) CPY/CNT	0.25 M $KHCO_3$	-0.70	15.2	94.0 (CO)	20	0.25	10 h (-0.55 V)	Sr[61]
CoPc/CNT	0.1 M $KHCO_3$	-0.77	12.6	95.0 (CO)	20	0.21	12 h (-1.00 V)	Sr[62]
Ag_1-MnO_2	0.5 M $KHCO_3$	-0.90	~5.0	~92.0 (CO)	15	0.21	9 h (-0.90 V)	Sr[63]
$Cu_{0.5}$NC	0.1 M $CsHCO_3$	-0.70	3.5	~75.0 (CO)	10	0.18	/	Sr[64]
PcCu-O_8-Zn/CNT	0.1 M $KHCO_3$	-1.00	14.0	55.0 (CO)	30	0.18	12 h (-0.70 V)	Sr[65]
Cu–APC	0.2 M $NaHCO_3$	-0.78	9.4	92.0 (CO)	20	0.15	3 h (-0.78 V)	Sr[66]
Bi SAs/NC	0.1 M $NaHCO_3$	-0.50	4.0	97.0 (CO)	20	0.14	4 h (-0.50 V)	Sr[67]

Catalysts	Electrolyte	Potential (V *vs.* RHE)	j_{total} ($mA \cdot cm^{-2}$)	FE (%)	CO_2 flow rate ($mL \cdot min^{-1}$)	CO_2 conv. (%)	Stability	Notes
Fe-CON_{400}-400	0.1 M $NaHCO_3$	-0.83	10.2	99.0 (CO)	27.4	0.13	12 h (-0.83 V)	Sr[68]
Au-2D	0.1 M $KHCO_3$	-0.90	16.3	66.6 (CO)	20	0.09	/	Sr[69]
DPC-NH_3-950	0.1 M $KHCO_3$	-0.60	2.8	95.2 (CO)	20	0.09	24 h (-0.60 V)	Sr[70]
$Au_{47}Cd_2(TBBT)_{31}$	0.5 M $KHCO_3$	-0.57	3.3	96.0 (CO)	10	0.06	20 h (-0.57 V)	Sr[71]

Thermocatalysis

Catalysts	Temperature, Pressure	Yield of product ($mmol \cdot g_{cat}^{-1} \cdot h^{-1}$)	Selectivity (%)	Reactant space velocity ($mL \cdot g_{cat}^{-1} \cdot h^{-1}$)	CO_2 conv. (%)	Notes
FeK/SWNTs	340 °C, 2.0 MPa	25.50 (C_{5+}), 14.28 (C_2-C_4), 6.20 (CH_4), 4.88 (CO)	50.1 (C_{5+}), 28.1 (C_2-C_4), 12.2 (CH_4), 9.6 (CO)	9000 (H_2:CO_2:N_2=72:24:4)	52.7	Sr[72]
FeK/MWNTs	340 °C, 2.0 MPa	11.10 (C_{5+}), 12.40 (C_2-C_4), 8.74 (CH_4), 9.84 (CO)	26.4 (C_{5+}), 29.5 (C_2-C_4), 20.8 (CH_4), 23.4 (CO)	9000 (H_2:CO_2:N_2=72:24:4)	43.6	Sr[72]
In–Zr/SAPO-34	300 °C, 3.0 MPa	3.94 (C_2-$C_4^{=}$), 0.86 (C_2-C_4^{o}), 29.10 (CO)	11.5 (C_2-$C_4^{=}$), 2.5 (C_2-C_4^{o}), 85.0 (CO)	9000 (H_2:CO_2:N_2=73:24:3)	35.5	Sr[73]
In–Zr/SAPO-34	300 °C, 3.0 MPa	8.96 (C_2-$C_4^{=}$), 0.98 (C_2-C_4^{o}), 38.27 (CO)	18.3 (C_2-$C_4^{=}$), 2.0 (C_2-C_4^{o}), 78.2 (CO)	15750 (H_2:CO_2:N_2=73:24:3)	29.0	Sr[73]
$ZnFeO_x$-Na/C-HZSM-5-a	320 °C, 3.0 MPa	8.28 (Aromatics), 2.13 (C_{5+}), 1.83 (C_2-C_4^{o}), 1.71 (CO)	42.0 (Aromatics), 22.1 (C_{5+}), 12.8 (C_2-C_4^{o}), 11.2 (CO)	4000 (H_2:CO_2:N_2=73:24:3)	36.5	Sr[74]
$ZnFeO_x$-Na/S-HZSM-5	320 °C, 3.0 MPa	6.57 (Aromatics), 3.46 (C_{5+}), 2.00 (C_2-C_4^{o}), 1.75 (CO)	53.4 (Aromatics), 13.7 (C_{5+}), 11.8 (C_2-C_4^{o}), 11.0 (CO)	4000 (H_2:CO_2:N_2=73:24:3)	36.2	Sr[74]
Na−$CoFe_2O_4$/CNT	340 °C, 1.0 MPa	12.35 (C_{5+}), 11.72 (C_2-$C_4^{=}$), 4.45 (CH_4), 6.90 (CO)	33.3 (C_{5+}), 31.6 (C_2-$C_4^{=}$), 12.0 (CH_4), 18.6 (CO)	3600 (H_2:CO_2:N_2=9:3:1)	34.4	Sr[75]
Co/CNT	340 °C, 1.0 MPa	36.83 (CH_4)	99.3 (CH_4)	3600 (H_2:CO_2:N_2=9:3:1)	36.1	Sr[75]
Cs-Cu-Fe-Zn	330 °C, 5.0 MPa	3.49 (C_{2+}OH)	19.8 (C_{2+}OH)	4500 (H_2:CO_2:N_2=72:24:4)	36.6	Sr[76]
Cr_2O_3/HZSM-5	350 °C, 3.0 MPa	1.79 (Aromatics), 0.19 (C_{5+}), 0.40 (C_2-C_4^{o}), 1.77 (CO)	41.5 (Aromatics), 4.5 (C_{5+}), 9.3 (C_2-C_4^{o}), 41.2 (CO)	1200 (H_2:CO_2:CO:Ar= 67.5:23.9:5.42:3.18)	33.6	Sr[77]
Na-Fe_3O_4/HZSM-5	320 °C, 3.0 MPa	5.98 (C_{5+}), 1.25 (C_2-C_4^{o}), 0.30 (CH_4), 1.90 (CO)	63.4 (C_{5+}), 13.3 (C_2-C_4^{o}), 3.2 C, 20.1 (CO)	4000 (H_2:CO_2:N_2=72:24:4)	22.0	Sr[78]
NP-MoC_{1-x}/C	300 °C, 2.0 MPa	1.44 (CH_4), 0.74 (C_{2+}), 6.09 (CO)	17.4 (CH_4), 8.9 (C_{2+}), 73.6 (CO)	3600 (H_2:CO_2:Ar=70:26:4)	19.8	Sr[79]
Fe/γ-Al_2O_3	300 °C, 2.0 MPa	3.53 (CH_4), 3.64 (C_{2+}), 1.30 (CO)	41.4 (CH_4), 42.7 (C_{2+}), 15.2 (CO)	3600 (H_2:CO_2:Ar=72:24:4)	22.1	Sr[79]
Cu/Co_3O_4-0h	250 °C, 3.0 MPa	61.01 (CH_4), 8.04 (C_{2+}HC), 3.31 (CO)	81.2 (CH_4), 10.7 (C_{2+}HC), 4.4 (CO)	36000 (H_2:CO_2=3:1)	18.7	Sr[80]
Cu/Co_3O_4-2h	250 °C, 3.0 MPa	21.17 (CH_4), 11.11 (C_{2+}HC), 8.49 (EtOH), 3.63 (CO)	37.9 (CH_4), 19.9 (C_{2+}HC), 15.2 (EtOH), 6.5 (CO)	36000 (H_2:CO_2=3:1)	13.9	Sr[80]
$CuFeO_2$−24	300 °C, 1.0 MPa	1.49 (C_{5+}), 1.05 (CO)	44.5 (C_{5+}), 31.4 (CO)	1800 (H_2:CO_2=3:1)	16.7	Sr[81]
RhFeLi/TiO_2	250 °C, 3.0 MPa	3.29 (C_{5+}), 1.31 (CO)	31.3 (C_{5+}), 12.5 (CO)	6000 (H_2:CO_2=3:1)	15.7	Sr[82]
$ZnAl_2O_4$/SAPO-34	370 °C, 3.0 MPa	4.01 (C_2-$C_4^{=}$), 0.46 (C_2-C_4^{o}), 3.84 (CO)	44.4 (C_2-$C_4^{=}$), 5.1 (C_2-C_4^{o}), 49 (CO)	5400 (H_2:CO_2=3:1)	15.0	Sr[83]
$ZnGa_2O_4$/SAPO-34	370 °C, 3.0 MPa	3.64 (C_2-$C_4^{=}$), 0.46 (C_2-C_4^{o}), 3.60 (CO)	46.4 (C_2-$C_4^{=}$), 5.9 (C_2-C_4^{o}), 46.0 (CO)	5400 (H_2:CO_2=3:1)	13.0	Sr[83]
In_2O_3/HZSM-5	340 °C, 3.0 MPa	5.48 (C_{5+}), 1.42 (C_2-C_4^{o}), 5.66 (CO)	43.4 (C_{5+}), 11.3 (C_2-C_4^{o}), 44.8 (CO)	9000 (H_2:CO_2:N_2=73:24:3)	13.1	Sr[84]
Zn-Zr/HZSM-5	340 °C, 3.0 MPa	1.25 (C_{5+}), 1.02 (C_2-C_4^{o}), 1.83 (CO)	30.0 (C_{5+}), 24.6 (C_2-C_4^{o}), 44.1 (CO)	9000 (H_2:CO_2:N_2=73:24:3)	4.3	Sr[84]
Fe/ZrO_2 (12.9)	320 °C, 3.0 MPa	7.39 (CH_4), 3.11 (C_2-C_4^{o}), 10.69 (CO)	34.2 (CH_4), 14.4 (C_2-C_4^{o}), 49.5 (CO)	18000 (H_2:CO_2:N_2=63:21:16)	12.8	Sr[85]
Fe/ZrO_2 (9.8)	320 °C, 3.0 MPa	6.13 (CH_4), 2.44 (C_2-C_4^{o}), 12.56 (CO)	28.6 (CH_4), 11.4 (C_2-C_4^{o}), 58.6 (CO)	18000 (H_2:CO_2:N_2=63:21:16)	12.7	Sr[85]
$ZnZrO_2$/SAPO-34	380 °C, 2.0 MPa	2.06 (C_{5+}), 2.28 (CO)	42.4 (C_{5+}), 47.0 (CO)	3600 (H_2:CO_2:Ar=72:24:4)	12.6	Sr[86]

Catalysts	Temperature, Pressure	Yield of product ($mmol \cdot g_{cat}^{-1} \cdot h^{-1}$)	Selectivity (%)	Reactant space velocity ($mL \cdot g_{cat}^{-1} \cdot h^{-1}$)	CO_2 conv. (%)	Notes
$ZnAlO_x$&HZSM-5	320 °C, 3.0 MPa	0.66 (C_{5+}), 1.11 (CO)	34.2 (C_{5+}), 57.4 (CO)	2000 (H_2:CO_2:Ar=3:1:0.2)	9.1	Sr[87]
Co@$Si_{0.95}$	320 °C, 2.0 MPa	3.25 (EtOH)	70.5 (EtOH)	6000 (H_2:CO_2:Ar=3:1:1)	8.6	Sr[88]
Cu@Na-Beta	300 °C, 1.3 MPa	6.77 (EtOH)	69.5 (EtOH)	12000 (H_2:CO_2:N_2=69:23:8)	7.9	Sr[89]
Cu/SiO_2	320 °C, 3.0 MPa	8.13 (MeOH)	21.4 (MeOH)	16000 (H_2:CO_2=4:1)	28.2	Sr[90]
CuO-ZnO-ZrO_2	280 °C, 5.0 MPa	15.31 (MeOH)	34.0 (MeOH)	~28600 (H_2:CO_2=4:1)	21.0	Sr[91]
CuO-ZnO-ZrO_2	260 °C, 5.0 MPa	13.44 (MeOH)	42.0 (MeOH)	~28600 (H_2:CO_2=4:1)	15.1	Sr[91]
Pd/In_2O_3	300 °C, 5.0 MPa	27.81 (MeOH)	72.1 (MeOH)	21000 (H_2:CO_2=4:1)	20.5	Sr[92]
Pd-ZnO	270 °C, 5.0 MPa	10.63 (MeOH)	35.7 (MeOH)	15000 (H_2:CO_2=3:1)	19.6	Sr[93]
Cu/Zn/Ga	270 °C, 4.5 MPa	18.44 (MeOH)	47.5 (MeOH)	18000 (H_2:CO_2=3:1)	18.6	Sr[94]
Cu/Zn/Ga	250 °C, 4.5 MPa	16.25 (MeOH)	51.0 (MeOH)	18000 (H_2:CO_2=3:1)	15.4	Sr[94]
Cu–ZnO–ZrO_2	220 °C, 3.0 MPa	9.77 (MeOH)	80.2 (MeOH)	6000 (H_2:CO_2=3:1)	18.2	Sr[95]
Cu/ZnO/Al_2O_3-2	260 °C, 5.0 MPa	12.81 (MeOH)	41.1 (MeOH)	15000 (H_2:CO_2=3:1)	18.2	Sr[96]
FL-MoS_2	280 °C, 5.0 MPa	31.25 (MeOH)	61.3 (MeOH)	36000 (H_2:CO_2=3:1)	12.5	Sr[96]
PdZn	290 °C, 4.5 MPa	20.00 (MeOH)	46.4 (MeOH)	21600 (H_2:CO_2=3:1)	17.9	Sr[97]
PdZn	270 °C, 4.5 MPa	20.31 (MeOH)	56.0 (MeOH)	21600 (H_2:CO_2=3:1)	14.9	Sr[97]
CuNi/CeO_2	260 °C, 3.0 MPa	18.13 (MeOH)	75.7 (MeOH)	~15000 (H_2:CO_2=3:1)	17.8	Sr[98]
Cu/AlCeO	260 °C, 3.0 MPa	11.88 (MeOH)	43.8 (MeOH)	14400 (H_2:CO_2=3:1)	16.9	Sr[99]
h–In_2O_3-R	360 °C, 5.0 MPa	10.90 (MeOH)	73.4 (MeOH)	9000 (H_2:CO_2:N_2=73:24:3)	15.4	Sr[100]
Pd/ZnO-3.93Al	250 °C, 3.0 MPa	4.51 (MeOH)	51.6 (MeOH)	6000 (H_2:CO_2:N_2=69:23:8)	14.2	Sr[101]
$Au^{\delta+}$–In_2O_{3-x}	300 °C, 5.0 MPa	14.13 (MeOH)	67.8 (MeOH)	21000 (H_2:CO_2:N_2=76:19:5)	11.7	Sr[102]
$Au^{\delta+}$–In_2O_{3-x}	275 °C, 5.0 MPa	10.70 (MeOH)	78.0 (MeOH)	21000 (H_2:CO_2:N_2=76:19:5)	7.7	Sr[102]
Pd@Zn	250 °C, 4.5 MPa	17.50 (MeOH)	77.6 (MeOH)	18000 (H_2:CO_2=3:1)	11.3	Sr[103]
Cu/ZnO/Al_2O_3	250 °C, 4.0 MPa	12.22 (MeOH)	54.8 (MeOH)	18000 (H_2:CO_2=3:1)	11.1	Sr[104]
CuZn@UiO-bpy	250 °C, 4.0 MPa	6.63 (MeOH)	100.0 (MeOH)	18000 (H_2:CO_2=3:1)	3.3	Sr[104]
ZnO-ZrO_2	320 °C, 5.0 MPa	21.88 (MeOH)	86.0 (MeOH)	18000 (H_2:CO_2=3:1)	10.0	Sr[105]
Pd-Cu/Zn	270 °C, 4.5 MPa	6.56 (MeOH)	64.0 (MeOH)	10800 (H_2:CO_2 =3:1)	8.3	Sr[106]
2Pd/CeO_2-R	240 °C, 3.0 MPa	0.71 (MeOH)	47.7 (MeOH)	2000 (H_2:CO_2=3:1)	5.9	Sr[107]
Cu/$La_2O_2CO_3$	240 °C, 3.0 MPa	6.56 (MeOH)	92.5 (MeOH)	12000 (H_2:CO_2=3:1)	5.6	Sr[108]
In_2O_3/ZrO_2	300 °C, 5.0 MPa	9.38 (MeOH)	99.8 (MeOH)	20000 (H_2/ CO_2=4/1)	5.2	Sr[109]
5In_2O_3/m-ZrO_2	280 °C, 5.0 MPa	8.64 (MeOH)	84.0 (MeOH)	24000 (H_2:CO_2=4/1)	4.8	Sr[110]

**Catalysts**	**Temperature, Pressure**	**Yield of product** **($mmol \cdot g_{cat}^{-1} \cdot h^{-1}$)**	**Selectivity** **(%)**	**Reactant space velocity** **($mL \cdot g_{cat}^{-1} \cdot h^{-1}$)**	**CO_2 conv.** **(%)**	**Notes**
$5In_2O_3$/m-ZrO_2	280 °C, 5.0 MPa	10.47 (MeOH)	94.0 (MeOH)	48000 (H_2:CO_2=4/1)	2.6	Sr[110]
ZrO_2/Cu	220 °C, 3.0 MPa	16.35 (MeOH)	70.0 (MeOH)	48000 (H_2:CO_2=3/1)	4.4	Sr[111]
Ni_5Ga_3	200 °C, 3.0 MPa	0.12 (MeOH)	100.0 (MeOH)	373.3 (H_2:CO_2=3/1)	3.0	Sr[112]
UiO-67-Pt	190 °C, 3.0 MPa	0.28 (MeOH)	42.0 (MeOH)	6000 (H_2:CO_2:N_2=21:7:1)	2.5	Sr[113]
UiO-67-Pt	170 °C, 0.8 MPa	0.06 (MeOH)	20.0 (MeOH)	6000 (H_2:CO_2:N_2=21:7:1)	1.2	Sr[114]
Cu/UiO-66	250 °C, 3.2 MPa	1.03 (MeOH)	30.0 (MeOH)	11667 (H_2:CO_2:N_2=21:7:1)	0.2	Sr[115]

Supplementary Table 2 | The *pH* and solution resistance (*R_s*) values. All CO_2-saturated $KHCO_3$ aqueous solutions with different $KHCO_3$ concentrations, and their R_s was obtained by the EIS measurements.

**Catalysts**	**$KHCO_3$ Concentration (M)**	***pH***	**R_s ($\Omega\cdot cm^2$)**
activated Ag HF	0.1	6.8	6.4
	0.5	7.2	1.8
	1.0	8.0	1.3
	1.5	8.2	0.9
	2.0	8.3	0.8
Ag HF	1.5	8.2	0.9
activated Ag foil	1.5	8.2	0.9
Ag foil	1.5	8.2	0.9

Supplementary References

1 Yasuda, H. & Tsai, J. T. Pore size of microporous polymer membranes. *J. Appl. Polym. Sci*. **18**, 805–819 (1974).

2 Nago, S. & Mizutani, Y. Microporous polypropylene hollow fibers with double layers. *J. Membr. Sci*. **116**, 1–7 (1996).

3 Bard, A. J. & Faulkner, L. R. *Electrochemical Methods: Fundamentals and Applications*. (Wiley & Sons, New York, 2001).

4 Yan, C. et al. Coordinatively unsaturated nickel nitrogen sites towards selective and high-rate CO_2 electroreduction. *Energy Environ. Sci*. **11** , 1204-1210 (2018).

5 Ma, W. et al. Promoting electrocatalytic CO_2 reduction to formate via sulfur-boosting water activation on indium surfaces. *Nat. Commun.* **10**, 892 (2019).

6 Rudolph, W. W., Irmer, G. & Königsberger, E. Speciation studies in aqueous HCO_3^-–CO_3^{2-} solutions. A combined Raman spectroscopic and thermodynamic study. *Dalton Trans.* **7**, 900–908 (2008).

7 Chen, X. Y. et al. Electrochemical CO_2-to-ethylene conversion on polyamine-incorporated Cu electrodes. *Nat. Catal.* **4**, 20–27 (2021).

8 Martina, I., Wiesinger, R., Jembrih-Simburger, D. & Schreiner, M. Micro-Raman characterisation of silver corrosion products. *Preservation Science* **9**, 1-8 (2012).

9 Yu, C., Li, G., Kumar, S., Yang, K. & Jin, R. Phase transformation synthesis of novel Ag_2O/Ag_2CO_3 heterostructures with high visible light efficiency in photocatalytic degradation of pollutants. *Adv. Mater.* **26**, 892-898 (2014).

10 Lu, Q. et al. A selective and efficient electrocatalyst for carbon dioxide reduction. *Nat. Commun.* **5**, 3242 (2014).

11 Ma, M., Trześniewski, B. J., Xie, J. & Smith, W. A. Selective and efficient reduction of carbon dioxide to carbon monoxide on oxide-derived nanostructured silver electrocatalysts. *Angew. Chem. Int. Ed.* **55**, 9748–9752 (2016).

12 Dunwell, M., Luc, W., Yan, Y., Jiao, F. & Xu, B. Understanding surface-mediated electrochemical reactions: CO_2 reduction and beyond. *ACS Catal.* **8**, 8121−8129 (2018).

13 Rosen, J. et al. Mechanistic insights into the electrochemical reduction of CO_2 to CO on nanostructured Ag surfaces. *ACS Catal*. **5**, 4293–4299 (2015).

14 Gu, J., Hsu, C. S., Bai, L., Chen, H. M. & Hu, X. Atomically dispersed Fe($^{3+}$) sites catalyze efficient CO_2 electroreduction to CO. *Science* **364**, 1091–1094 (2019).

15 Hsieh, Y-C., Senanayake, S. D., Zhang, Y., Xu, W. & Polyansky, D. E. Effect of chloride anions on the synthesis and enhanced catalytic activity of silver nanocoral electrodes for CO_2 electroreduction. *ACS Catal*. **5**, 5349–5356 (2015).

16 Zhang, Y. et al. Iodide-derived nanostructured silver promotes selective and efficient carbon dioxide conversion into carbon monoxide. *Chem. Commun*. **54**, 2666−2669 (2018).

17 Hsieh, Y-C. et al. Modification of CO_2 reduction activity of nanostructured silver electrocatalysts by surface halide anions. *ACS Appl. Energy Mater*. **2**, 102−109 (2019).

18 Shan, W. Y. et al. In situ surface-enhanced Raman spectroscopic evidence on the origin of selectivity in CO_2 electrocatalytic reduction. *ACS Nano* **14**, 11363–11372 (2020).

19 Yan, X. P. et al. Efficient electroreduction of CO_2 to C_{2+} products on CeO_2 modified CuO. *Chem. Sci.*, **12**, 6638–6645 (2021).

20 Iwasita, T., Rodes, A. & Pastor, E. Vibrational spectroscopy of carbonate adsorbed on Pt (111) and Pt (110) single-crystal electrodes. *J. Electroanal. Chem.* **383**, 181−189 (1995).

21 Chernyshova, I. V., Somasundaran, P. & Ponnurangam, S. On the origin of the elusive first intermediate of CO_2 electroreduction. *Proc. Nat. Acad. Sci. USA* **115**, E9261–E9270 (2018).
22 Moradzaman, M. & Mul, G. In situ Raman study of potential-dependent surface adsorbed carbonate, CO, OH, and C species on Cu electrodes during electrochemical reduction of CO_2. *ChemElectroChem* **8**, 1478−1485 (2021).
23 Ma, W. et al. Electrocatalytic reduction of CO_2 to ethylene and ethanol through hydrogen-assisted C–C coupling over fluorine-modified copper. *Nat. Catal.* **3**, 478–487 (2020).
24 Ye, K. et al. In situ reconstruction of a hierarchical Sn-Cu/SnO_x core/shell catalyst for high-performance CO_2 electroreduction. *Angew. Chem. Int. Ed.* **59**, 4814–4821 (2020).
25 Grigioni, I. et al. CO_2 electroreduction to formate at a partial current density of 930 mA cm^{-2} with InP colloidal quantum dot derived catalysts. *ACS Energy Lett.* **6**, 79–84 (2020).
26 Chen, Z. et al. Amination strategy to boost the CO_2 electroreduction current density of M–N/C single-atom catalysts to the industrial application level. *Energy Environ. Sci.* **14**, 2349–2356 (2021).
27 Gao, F. Y. et al. High-curvature transition-metal chalcogenide nanostructures with a pronounced proximity effect enable fast and selective CO_2 electroreduction. *Angew. Chem. Int. Ed.* **59**, 8706–8712 (2020).
28 Yang, D. et al. Electrosynthesis of a defective indium selenide with 3D structure on a substrate for tunable CO_2 electroreduction to syngas. *Angew. Chem. Int. Ed.* **59**, 2354–2359 (2020).
29 García de Arquer, F. P. et al. CO_2 electrolysis to multicarbon products at activities greater than 1 A cm^{-2}. *Science* **367**, 661–666 (2020).
30 Wang, M. et al. CO_2 electrochemical catalytic reduction with a highly active cobalt phthalocyanine. *Nat. Commun.* **10**, 3602 (2019).
31 Zhang, X. et al. Molecular engineering of dispersed nickel phthalocyanines on carbon nanotubes for selective CO_2 reduction. *Nat. Energy* **5**, 684–692 (2020).
32 Fan, L., Xia, C., Zhu, P., Lu, Y. & Wang, H. Electrochemical CO_2 reduction to high-concentration pure formic acid solutions in an all-solid-state reactor. *Nat. Commun.* **11**, 3633 (2020).
33 Shi, R. et al. Efficient wettability-controlled electroreduction of CO_2 to CO at Au/C interfaces. *Nat. Commun.* **11**, 3028 (2020).
34 Chen, C. et al. Boosting CO_2 electroreduction on N,P-Co-doped carbon aerogels. *Angew. Chem. Int. Ed.* **59**, 11123–11129 (2020).
35 Duan, Y. X. et al. Boosting production of HCOOH from CO_2 electroreduction via Bi/CeO_x. *Angew. Chem. Int. Ed.* **60**, 8798–8802 (2021).
36 Ren, S. et al. Molecular electrocatalysts can mediate fast, selective CO_2 reduction in a flow cell. *Science* **365**, 367–369 (2019).
37 Li, F. et al. Molecular tuning of CO_2-to-ethylene conversion. *Nature* **577**, 509–513 (2020).
38 Su, J. et al. Building a stable cationic molecule/electrode interface for highly efficient and durable CO_2 reduction at an industrially relevant current. *Energy Environ. Sci.* **14**, 483–492 (2021).
39 Wang, X. et al. Proton capture strategy for enhancing electrochemical CO_2 reduction on atomically dispersed metal-nitrogen active sites. *Angew. Chem. Int. Ed.* **60**, 11959–11965 (2021).

40 Li, H. Y. et al. Edge-exposed molybdenum disulfide with N-doped carbon hybridization: A hierarchical hollow electrocatalyst for carbon dioxide reduction. *Adv. Energy Mater.* **9**, 1900072 (2019).
41 Sun, X. et al. Aqueous CO_2 reduction with high efficiency using alpha-$Co(OH)_2$ -supported atomic Ir electrocatalysts. *Angew. Chem. Int. Ed.* **58**, 4669–4673 (2019).
42 Wu, Y. Z. et al. Beyond d orbits: Steering the selectivity of electrochemical CO_2 reduction via hybridized sp band of sulfur-incorporated porous Cd architectures with dual collaborative sites. *Adv. Energy Mater.* **10**, 2002499 (2020).
43 Zhang, T. et al. Atomically dispersed nickel(I) on an alloy-encapsulated nitrogen-doped carbon nanotube array for high-performance electrochemical CO_2 reduction reaction. *Angew. Chem. Int. Ed.* **59**, 12055–12061 (2020).
44 Li, F. et al. Cooperative CO_2-to-ethanol conversion via enriched intermediates at molecule–metal catalyst interfaces. *Nat. Catal.* **3**, 75–82 (2019).
45 Kim, D. et al. Selective CO_2 electrocatalysis at the pseudocapacitive nanoparticle/ordered-ligand interlayer. *Nat. Energy* **5**, 1032–1042 (2020).
46 Dinh, C.-T., García de Arquer, F. P., Sinton, D. & Sargent, E. H. High rate, selective, and stable electroreduction of CO_2 to CO in basic and neutral media. *ACS Energy Lett.* **3**, 2835–2840 (2018).
47 He, C. et al. Molecular evidence for metallic cobalt boosting CO_2 electroreduction on pyridinic nitrogen. *Angew. Chem. Int. Ed.* **59**, 4914–4919 (2020).
48 Ni, W. et al. Nonnitrogen coordination environment steering electrochemical CO_2-to-CO conversion over single-atom tin catalysts in a wide potential window. *ACS Catal.* **11**, 5212–5221 (2021).
49 Ni, W. et al. Electroreduction of carbon dioxide driven by the intrinsic defects in the carbon plane of a single Fe-N_4 site. *Adv. Mater.* **33**, e2003238 (2021).
50 Fan, Z. et al. Heterophase fcc-2H-fcc gold nanorods. *Nat. Commun.* **11**, 3293 (2020).
51 Ju, W. B. et al. Electrocatalytic reduction of gaseous CO_2 to CO on Sn/Cu-nanofiber-based gas diffusion electrodes. *Adv. Energy Mater.* **9**, 190154 (2019).
52 Wang, Y. X. et al. Ensemble effect in bimetallic electrocatalysts for CO_2 reduction. *J. Am. Chem. Soc.* **141**, 16635–16642 (2019).
53 Jiao, L. et al. Single-atom electrocatalysts from multivariate metal-organic frameworks for highly selective reduction of CO_2 at low pressures. *Angew. Chem. Int. Ed.* **59**, 20589–20595 (2020).
54 Li, T. F., Yang, C., Luo, J. L. & Zheng, G. F. Electrolyte driven highly selective CO_2 electroreduction at low overpotentials. *ACS Catal.* **9**, 10440–10447 (2019).
55 Ren, W. Isolated diatomic Ni-Fe metal-nitrogen sites for synergistic electroreduction of CO_2. *Angew. Chem. Int. Ed.* **58**, 6972–6976 (2019).
56 Saberi Safaei, T. et al. High-density nanosharp microstructures enable efficient CO_2 electroreduction. *Nano Lett.* **16**, 7224–7228 (2016).
57 Hu, C. et al. Porosity-induced high selectivity for CO_2 electroreduction to CO on Fe-doped ZIF-derived carbon catalysts. *ACS Catal.* **9**, 11579–11588 (2019).
58 Narouz, M. R. et al. N-heterocyclic carbene-functionalized magic-number gold nanoclusters. *Nat. Chem.* **11**, 419–425 (2019).
59 Zhang, N. et al. A supported Pd_2 dual-atom site catalyst for efficient electrochemical CO_2 reduction. *Angew. Chem. Int. Ed.* **60**, 13388–13393 (2021).

60 Zhao, S. et al. A universal seeding strategy to synthesize single atom catalysts on 2D materials for electrocatalytic applications. *Adv. Funct. Mater.* **30**, 1906157 (2019).
61 Sun, L. et al. A planar, conjugated N4-macrocyclic cobalt complex for heterogeneous electrocatalytic CO_2 reduction with high activity. *Angew. Chem. Int. Ed.* **59**, 17104–17109 (2020).
62 Wu, Y., Jiang, Z., Lu, X. Liang, Y. & Wang, H. Domino electroreduction of CO_2 to methanol on a molecular catalyst. *Nature* **575**, 639–642 (2019).
63 Zhang, N. et al. Silver single-atom catalyst for efficient electrochemical CO_2 reduction synthesized from thermal transformation and surface reconstruction. *Angew. Chem. Int. Ed.* **60**, 6170–6176 (2021).
64 Karapinar, D. et al. Electroreduction of CO_2 on single-site copper-nitrogen-doped carbon material: Selective formation of ethanol and reversible restructuration of the metal sites. *Angew. Chem. Int. Ed.* **58**, 15098–15103 (2019).
65 Zhong, H. et al. Synergistic electroreduction of carbon dioxide to carbon monoxide on bimetallic layered conjugated metal-organic frameworks. *Nat. Commun.* **11**, 1409 (2020).
66 Jiao, J. et al. Copper atom-pair catalyst anchored on alloy nanowires for selective and efficient electrochemical reduction of CO_2. *Nat. Chem.* **11**, 222–228 (2019).
67 Zhang, E. H. et al. Bismuth single atoms resulting from transformation of metal-organic frameworks and their use as electrocatalysts for CO_2 reduction. *J. Am. Chem. Soc.* **141**, 16569–16573 (2019).
68 Chen, Z. et al. Fe_1N_4–O_1 site with axial Fe–O coordination for highly selective CO_2 reduction over a wide potential range. *Energy Environ. Sci.* **14**, 3430-3437 (2021).
69 Kwok, K. S. et al. Nano-folded gold catalysts for electroreduction of carbon dioxide. *Nano Lett.* **19**, 9154–9159 (2019).
70 Dong, Y. et al. Ammonia thermal treatment toward topological defects in porous carbon for enhanced carbon dioxide electroreduction. *Adv. Mater.* **32**, e2001300 (2020).
71 Zhuang, S. et al. Hard-sphere random close-packed $Au_{47}Cd_2(TBBT)_{31}$ nanoclusters with a faradaic efficiency of up to 96 % for electrocatalytic CO_2 reduction to CO. *Angew. Chem. Int. Ed.* **59**, 3073-3077 (2020).
72 Wang, S. W. et al. Iron-potassium on single-walled carbon nanotubes as efficient catalyst for CO_2 hydrogenation to heavy olefins. *ACS Catal.* **10**, 6389–6401 (2020).
73 Gao, P. et al. Direct production of lower olefins from CO_2 conversion via bifunctional catalysis. *ACS Catal.* **8**, 571–578 (2018).
74 Cui, X. et al. Selective production of aromatics directly from carbon dioxide hydrogenation. *ACS Catal.* **9**, 3866–3876 (2019).
75 Kim, K. Y. et al. Cobalt ferrite nanoparticles to form a catalytic Co-Fe alloy carbide phase for selective CO_2 hydrogenation to light olefins. *ACS Catal.* **10**, 8660–8671 (2020).
76 Xu, D., Ding, M. Y., Hong, X. L., Liu, G. L. & Tsang, S. C. E. Selective C_{2+} alcohol synthesis from direct CO_2 hydrogenation over a Cs-promoted Cu-Fe-Zn catalyst. *ACS Catal.* **10**, 5250–5260 (2020).
77 Wang, Y. et al. Rationally designing bifunctional catalysts as an efficient strategy to boost CO_2 hydrogenation producing value-added aromatics. *ACS Catal.* **9**, 895–901 (2019).
78 Wei, J. et al. Directly converting CO_2 into a gasoline fuel. *Nat. Commun.* **8**, 15174 (2017).
79 Baddour, F. G. et al. An exceptionally mild and scalable solution-phase synthesis of molybdenum carbide nanoparticles for thermocatalytic CO_2 hydrogenation. *J. Am. Chem. Soc.* **142**, 1010–1019 (2020).

80 Yang, C. et al. The interplay between structure and product selectivity of CO_2 hydrogenation. *Angew. Chem. Int. Ed.* **58**, 11242–11247 (2019).
81 Choi, Y. H. et al. Carbon dioxide Fischer-Tropsch synthesis: A new path to carbon-neutral fuels. *Appl. Catal. B–Environ.* **202**, 605–610 (2017).
82 Yang, C. et al. Hydroxyl-mediated ethanol selectivity of CO_2 hydrogenation. *Chem. Sci*. **10**, 3161–3167 (2019).
83 Liu, X. L. et al. Tandem catalysis for hydrogenation of CO and CO_2 to lower olefins with bifunctional catalysts composed of spinel oxide and SAPO-34. *ACS Catal.* **10**, 8303–8314 (2020).
84 Gao, P. et al. Direct conversion of CO_2 into liquid fuels with high selectivity over a bifunctional catalyst. *Nat. Chem.* **9**, 1019–1024 (2017).
85 Zhu, J. et al. Deconvolution of the particle size effect on CO_2 hydrogenation over iron-based catalysts. *ACS Catal.* **10**, 7424–7433 (2020).
86 Li, Z. et al. Highly selective conversion of carbon dioxide to lower olefins. *ACS Catal.* **7**, 8544–8548 (2017).
87 Ni, Y. et al. Selective conversion of CO_2 and H_2 into aromatics. *Nat. Commun.* **9**, 3457 (2018).
88 Wang, L. X. et al. Silica accelerates the selective hydrogenation of CO_2 to methanol on cobalt catalysts. *Nat. Commun.* **11**, 1033 (2020).
89 Ding, L. P. et al. CO_2 hydrogenation to ethanol over Cu@Na-Beta. *Chem.* **6**, 2673-2689 (2020).
90 Wang, Z.-Q. et al. High-performance and long-lived Cu/SiO_2 nanocatalyst for CO_2 hydrogenation. *ACS. Catal.* **5**, 4255–4259 (2015).
91 Angelo, L. et al. Catalyst synthesis by continuous coprecipitation under micro-fluidic conditions: Application to the preparation of catalysts for methanol synthesis from CO_2/H_2. *Catal. Today* **270**, 59–67 (2016).
92 Rui, N. et al. CO_2 hydrogenation to methanol over Pd/In_2O_3: Effects of Pd and oxygen vacancy. *Appl. Catal. B–Environ.* **218**, 488–497 (2017).
93 Liang, X.-L., Xie, J.-R. & Liu, Z.-M. A novel Pd-decorated carbon nanotubes-promoted Pd-ZnO catalyst for CO_2 hydrogenation to methanol. *Catal. Lett.* **145**, 1138–1147 (2015).
94 Li, M. M. J. et al. CO_2 hydrogenation to methanol over catalysts derived from single cationic layer CuZnGa LDH precursors. *ACS Catal.* **8**, 4390–4401 (2018).
95 Wang, Y. H. et al. Exploring the ternary interactions in Cu-ZnO-ZrO_2 catalysts for efficient CO_2 hydrogenation to methanol. *Nat. Commun.* **10**, 1166 (2019).
96 Hu, J. et al. Sulfur vacancy-rich MoS_2 as a catalyst for the hydrogenation of CO_2 to methanol. *Nat. Catal.* **4**, 242–250 (2021).
97 Yin, Y. et al. Pd@zeolitic imidazolate framework-8 derived PdZn alloy catalysts for efficient hydrogenation of CO_2 to methanol. *Appl. Catal. B–Environ.* **234**, 143–152 (2018).
98 Tan, Q., Shi, Z. & Wu, D. CO_2 hydrogenation to methanol over a highly active Cu-Ni/CeO_2-nanotube catalyst. *Ind. Eng. Chem. Res.* **57**, 10148–10158 (2018).
99 Li, S. Guo, L. & Ishihara, T. Hydrogenation of CO_2 to methanol over Cu/AlCeO catalyst. *Catal. Today*. **339**, 352-361 (2020).
100 Dang, S. S. et al. Rationally designed indium oxide catalysts for CO_2 hydrogenation to methanol with high activity and selectivity. *Sci. Adv.* **6**, eaaz2060 (2020).
101 Song, J. M. et al. The role of Al doping in Pd/ZnO catalyst for CO_2 hydrogenation to methanol. *Appl. Catal. B-Environ.* **263**, 118367 (2020).

102 Rui, N. et al. Hydrogenation of CO_2 to methanol on a $Au^{\delta+}$-In_2O_{3-x} catalyst. *ACS Catal.* **10**, 11307–11317 (2020).
103 Liao, F. et al. A promising low pressure methanol synthesis route from CO_2 hydrogenation over Pd@Zn core-shell catalysts. *Green Chem.* **19**, 270–280 (2017).
104 An, B. et al. Confinement of ultrasmall Cu/ZnOx nanoparticles in metal-organic frameworks for selective methanol synthesis from catalytic hydrogenation of CO_2. *J. Am. Chem. Soc.* **139**, 3834–3840 (2017).
105 Wang, J. et al. A highly selective and stable ZnO-ZrO_2 solid solution catalyst for CO_2 hydrogenation to methanol. *Sci. Adv.* **3**, e1701290 (2017).
106 Hu, B. et al. Hydrogen spillover enabled active Cu sites for methanol synthesis from CO_2 hydrogenation over Pd doped CuZn catalysts. *J. Catal.* **359**, 17–26 (2018).
107 Jiang, F. et al. Insights into the Influence of CeO_2 Crystal Facet on CO_2 Hydrogenation to methanol over Pd/CeO_2 catalysts. *ACS Catal.* **10**, 11493–11509 (2020).
108 Chen, K., Duan, X., Fang, H., Liang, X. & Yuan, Y. Selective hydrogenation of CO_2 to methanol catalyzed by Cu supported on rod-like $La_2O_2CO_3$. *Catal. Sci. Technol.* **8**, 1062–1069 (2018).
109 Martin, O. et al. Indium oxide as a superior catalyst for methanol synthesis by CO_2 hydrogenation. *Angew. Chem. Int. Ed.* **55**, 6261–6265 (2016).
110 Frei, M. S. et al. Role of zirconia in indium oxide-catalyzed CO_2 hydrogenation to methanol. *ACS Catal.* **10**, 1133–1145 (2020).
111 Wu, C. Y. et al. Inverse ZrO_2/Cu as a highly efficient methanol synthesis catalyst from CO_2 hydrogenation. *Nat. Commun.* **11**, 5767 (2020).
112 Men, Y. H. et al. Synthesis of Ni_5Ga_3 catalyst by hydrotalcite-like compound (HTlc) precursors for CO_2 hydrogenation to methanol. *Appl. Catal. B-Environ.* **275**, 119067 (2020).
113 Gutterod, E. S. et al. Influence of defects and H_2O on the hydrogenation of CO_2 to methanol over Pt nanoparticles in UiO-67 metal-organic framework. *J. Am. Chem. Soc.* **142**, 17105–17118 (2020).
114 Gutterod, E. S. et al. Hydrogenation of CO_2 to methanol by Pt nanoparticles encapsulated in UiO-67: deciphering the role of the metal-organic framework. *J. Am. Chem. Soc.* **142**, 999–1009 (2020).
115 Zhu, Y. F. et al. Copper-zirconia interfaces in UiO-66 enable selective catalytic hydrogenation of CO_2 to methanol. *Nat. Commun.* **11**, 5849 (2020).
